# Supplementary material for: The Good, the Bad, or Both? Unveiling the Molecular Functions of LINC01133 in Tumors
Source: Noncoding RNA. 2025 Jul 30;11(4):58. doi: 10.3390/ncrna11040058 (PMC12389352; doi:10.3390/ncrna11040058)

Article

The Good, The Bad, or Both? Unveiling the molecular functions of LINC01133 in tumors

Leandro Teodoro Júnior [1,2,*] and Mari Cleide Sogayar [1,2]

[1] Cell and Molecular Therapy NUCEL Group, School of Medicine, University of Sao Paulo, Sao Paulo 01246-903, SP, Brazil; mcsoga@iq.usp.br
[2] Biochemistry Department, Chemistry Institute, University of Sao Paulo, Sao Paulo 05508-900, SP, Brazil
* Correspondence: teoolt.bio@gmail.com

Supplementary Material SM1

Conformational predicted structure of LINC01133 isoforms

>ENST00000772280.1 lncRNA

```
GGUUGCAGUGAAAAGCUGGUCCUGCUGUGGUGGAGAGAAUGGAGGAAAGAUAAU
AAAAGGCCAAACCUUUGCUCCAACUUUCUCCUUAGCUUCCCUUUGGAUCUGGAA
AGCUGGGGACCCACACGGCAGAGCCAUGGUACUGGAGGAGCCAUUAACAACGAG
GCUCCUUCUGUAAGGUUGCCUGGAUACUGCAUCUGGCUGACAGAGUCCCCAGAC
AGAACUUGAAUAGGCACUGCCAGGGUGUGUACUUCUAUGACUCUCCUUUUGCAA
UGGGCCCACACCACCCACCUCAUUUUUCUAAUAAGCCCCUGCCAUUCACACUGUG
CAAAGGAGCCUGUCAAUCACUGACUGAUUUUAAGCAGGAAAGUUUCAAAGAAAG
CCCCCAGUACUUACCAAUGUUAAAGAAAAACAGGCCAGUGCUGAAUAAAUGCUU
GUGCUAUUGUGACGCUUCUGACCCAGGCAGGAGACAUGUGCUUCCAUCUCCAGA
ACAGCAACCCCUGAAUGACCCCUCGAUACAAUGGAGAAAAGAAGUGCUGUGUAA
UUUAGGGGCAUAAACCAAACCUCCACUGCUUAGAGUCAGCUUAAUGUCUGACAG
AAAUAUACAAAACUGUAAAGCCUCAACCAGACAUCUGAAUAAUCUGUCCAUACA
UUUUUCACAUAUCAGUAUUUAUUAUCUUUUCAAAGGAACAGGUCAAGAUGAGA
AUAAAGUUUAUGAUC
```

The free energy of the thermodynamic ensemble is **–210.26** kcal/mol.

The frequency of the MFE structure in the ensemble is **0.00** %.

The ensemble diversity is **100.95**.

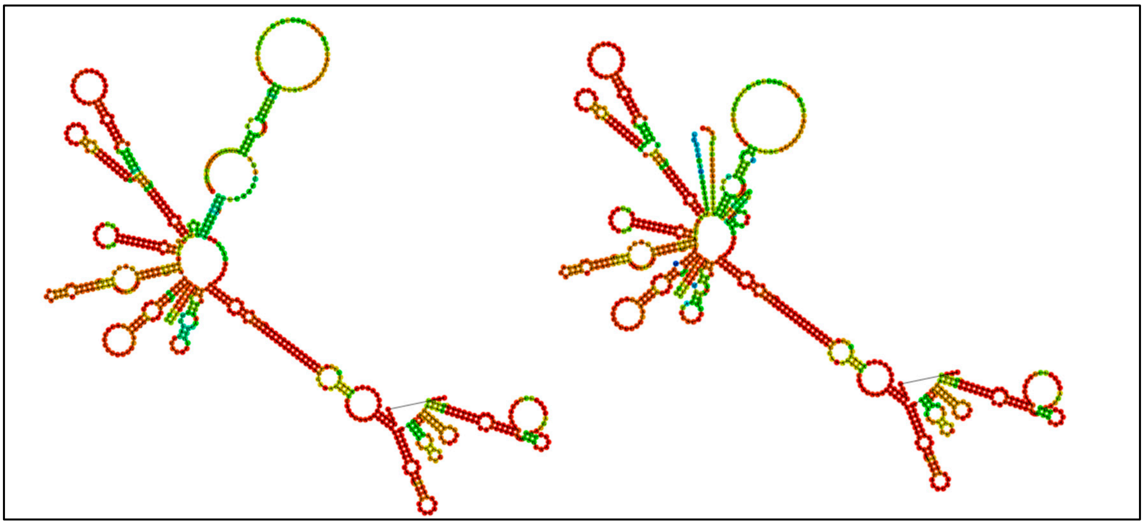

MFE secondary structure and Centroid secondary structure, respectively.

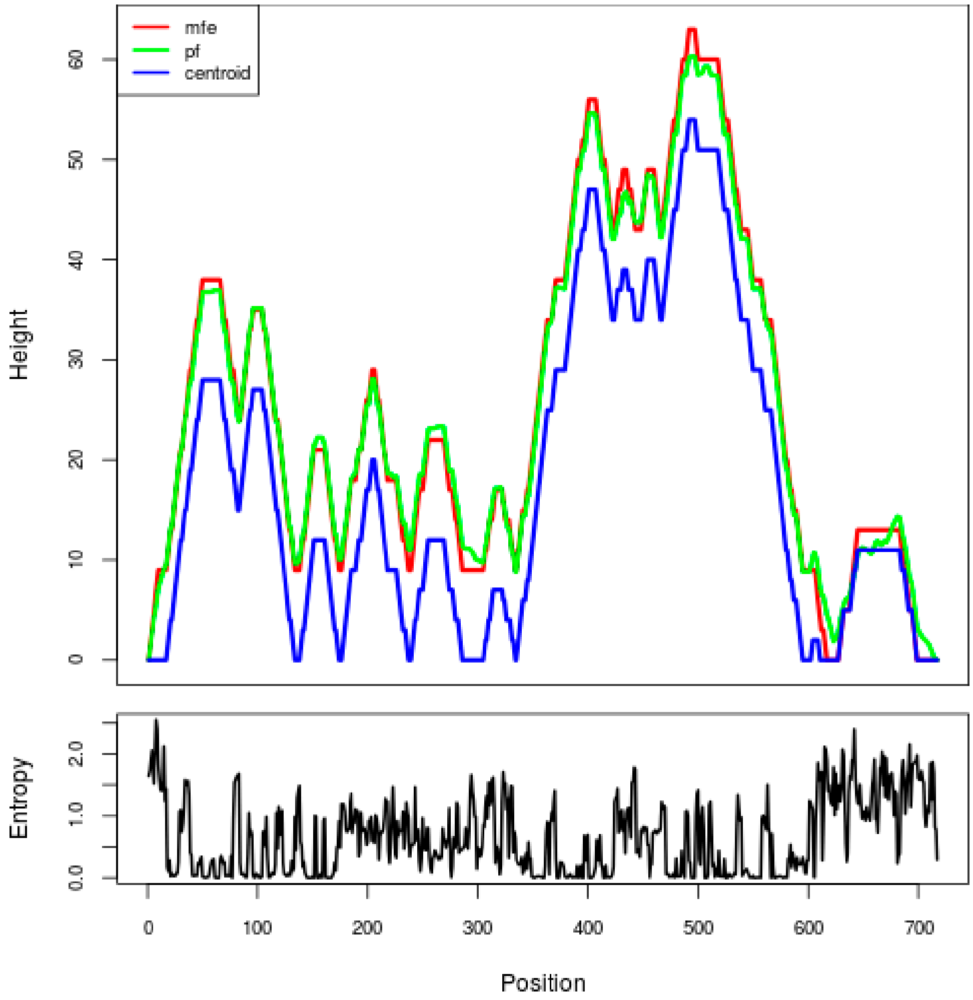

>ENST00000635112.2 lncRNA

```
GACUUAGCAGGGAGCUCUGGCACCGACUGCCCGUUAGAGAAGGCCUGUGGUGGG
CAGGCAUGUCCAGGCACUGGGACUCUAGCUGUGCAAGACUGUCCUGGUGCCAGG
ACUCUUGCUGUGCACAGCCAUGGGCUGGGGCUGCCGGGAACAGCGUAGAUCCCG
AAGGUGCUGCAGCUAGAGCCUGUCGGUUAACUGCACUUCUUGCAGCCAAAAAUG
UGUUUACUCUUGAAGGGGGAGCCGAGGCUGCUACAGAGCAAUUAAGCCAAUGAG
AAAUCAGACCCACACCCCAAUUCUGAUGUAACAGCCUUGGGAAAGAGGUUGCAG
UGAAAAGCUGGUCCUGCUGUGGUGGAGAGAAUGGAGGAAAGAUAAUAAAAGGCC
AAACCUUUGCUCCAACUUUCUCCUUAGCUUCCCUUUGGAUCUGGAAAGCUGGGG
ACCCACACGGCAGAGCCAUGGUACUGGAGGAGCCAUUAACAAAUGGAGUUUCAA
UCUUGUUGCCCAGGCUGGAGUGCAAUGGUGCGAUCUCAGUUCACCGCAACCUCC
GCCUCCCAGGAGAAUCGCUUGAACCCGGGAGGCAGAGGUUGCAGUGAGCUGAGA
UUGCACCACUGUACUCCAGCCUGGUGACAGAGUGAGACUCCGUCUCAAAAAAAU
AAAAAAAGAAAAAAAGAAACAUAUCAACAUUAGAGUUUCUAGCUUAUAUCCCAA
UAUCUUGGCCAUUCAUUAGAACCCUGGUUAGGCUCCUCUCUUAUCAACUUACCC
UCAUGACCAGCUUCUGCCUCCUGUUCCCAUCAGAGGAGGUUUAAGGCCAAUUAG
CAACAUGUGUCUUUCAUCCUUUUCUUUUCUUCUACUGUGGAGGUUCAUCACAGC
ACUUCUAUCAGUUCACCAGAUGAAAGAGGCAUCCAUCUGUGCCUCUUCAUCUGU
UGGUUGUAAUGCAGCACAUAUGAUAAGUGGGUAUUGAGUGGACCUGGACUGGAG
UCACAAUCACUGAUUAUGUGCUGGAAGUCAGAGGGGGACAGGGAAAGUCCCCCU
UUCCUAGGAAAAGGGUCCUCAAGUUGCCAAGAAAAGCAAGAGAUCAAAUUCCCC
CGAGGAGUGGAUUUCUGACCACUCCUUGGUUUGUUUUUUGUUUUUGUUUUGUUU
UGUUUGUUUUUUGCUUGAGUUAAUUUAAGUUAGUUUCUAUUACUUGCAACUAA
GAAGUUUUAAUGAAUUCACUUGAAAUACUAUUGUUAGGAAGGAAACAACUUGC
UUGAGAGGUCAAGCUUAGGCUGAAAGUCAACAUUGGCCAAGGCACUUAGAGAGC
AAUUAAUCCUCGUGGCAAGGCAGGGAUGGGGUGGAGGGGAUAGAUGAUUCCUAA
GGUCCUUUCUAAGAGGAUAAGAGUGCUUCCCCUGGUCAGUGGUUCUGGCUAGUC
UCCUCCCUCCCUUCAAAAUGCGAAGCAAAAGGCAGGGAGAAGGUCUCCUUGCAG
AUCUUCAGCUCUCUCAGGGAAAGAGACCCACGUCACUCCCAGCAGAGUCCUGCAU
GGCAGCAAGGGGCCAGGGCGGAAGAAGAACAAAAGACACCUGGAGAAGAAAGGC
AGUGCCUUUCCCUGCAGCAGCCAGGGCUGCAGAUAUGAUGGAGCAGGGGAGAUG
GUCUCUUCUAGAGCAGCAUGAGCCGAGUUACACAGAUGAUGUAAGGAAGAGUAG
AGAAACUGCAGUCUCAGCUCAGAGUUUUCCUUACAUGCUUGUGAAAAUAAAGUC
ACCCUCUGUUGCAGGCUGAAUAAUGGCCCCCCAGGAUGUCCACAUUCUAAUCCCU
GGAACCUGUCCAUGUUUUAUUAUAUGGCAAAAGAGACUUUGUAGAUGUGAUUA
AAUUAAGGCUCUUGAGAUUGGGAGAUUAUCCUGGAUUAUCAGAGUAGGCUUGAU
GUAAUCACAAGAGUCCUUCUAAGAGAAAAGCAGGAUGCAGCCCCCUGAUGACAG
AAGCAGAGAGGAAAGCAGAGUUGGAGACAGAAGACGCUAUGCUAUUGGCUUCCU
AAAGACAGAGGAAAGGGCCACAAACCAAAGAAUACACUUGUAUUCUUCAGUAAA
UAGCAGCUGCCUUGGAGUGUUGCUUGAUUCAAUACAUGUAAUAGUAAUCGUUCA
AUAAAUGCUUGCUAUUAUACCCUUGGAUGUCUUAGAGCUGCACUGUCCAAUAUG
GUAGCCACAAGCCACAUGUGACCAUUCGACACAGGUGUGGAUUUUCCGAAUUGA
GGUAUGCUGUAUGUGUAAAAUAACACACCAAAAUUCAGAUACUGAGUACAAAAC
AAAGAAUGUAAAAUAGCUCAGUAAUCUUAAUAUUAAUUAUUUGUUGAAAUAAC
AUUUUUAACACAUCCAAUUAAAUAAAAUAUAUUAAAAUUAAA
```

The free energy of the thermodynamic ensemble is **–889.31** kcal/mol.

The frequency of the MFE structure in the ensemble is **0.00** %.

The ensemble diversity is **596.39**.

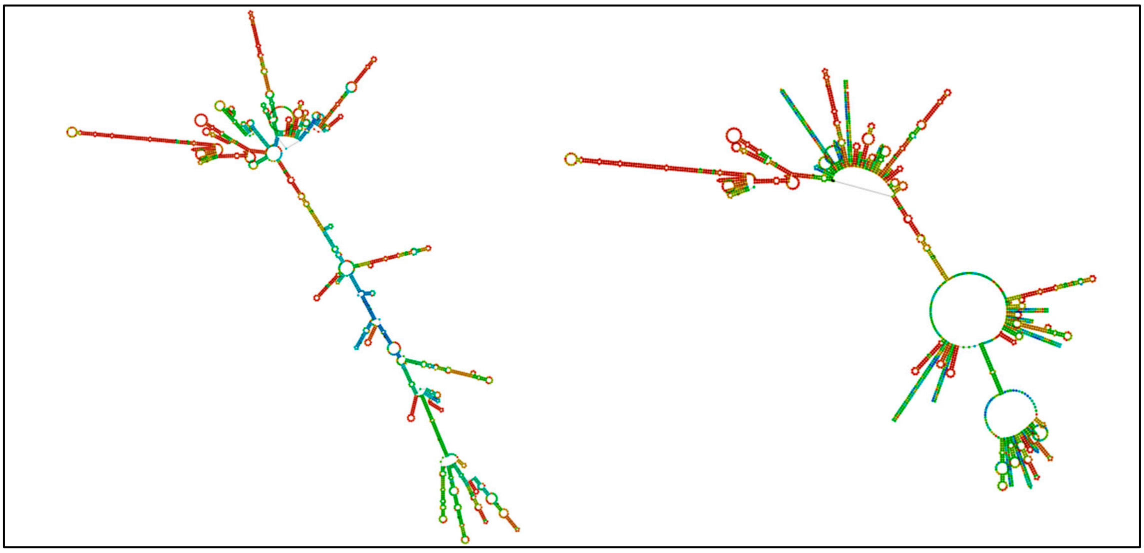

MFE secondary structure and Centroid secondary structure, respectively.

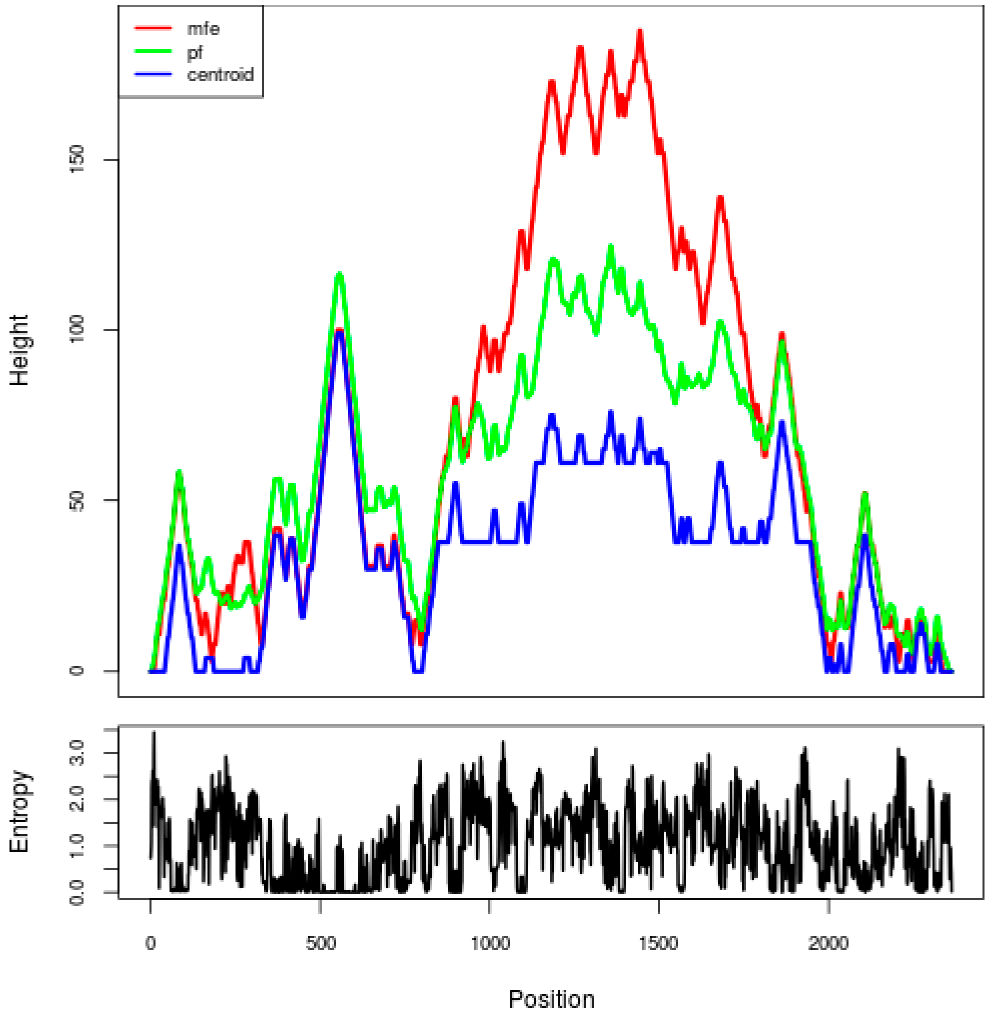

>ENST00000423943.4 lncRNA

```
AAGGGUGUGCUUUGGAGAGAGACCAAACUAUGACAUUAUUAUAUAUAAUAAAG
UAGUUGCACCAGUAAUGUUUAGACUUUCUCUCUCUUCUUCCUGGAUCUUUAGGA
GCAAUUAAGCCAAUGAGAAAUCAGACCCACACCCCAAUUCUGAUGUAACAGCCU
UGGGAAAGAGGUUGCAGUGAAAAGCUGGUCCUGCUGUGGUGGAGAGAAUGGAGG
AAAGAUAAUAAAAGGCCAAACCUUUGCUCCAACUUUCUCCUUAGCUUCCCUUUG
GAUCUGGAAAGCUGGGGACCCACACGGCAGAGCCAUGGUACUGGAGGAGCCAUU
AACAAAGCUUUCAAUAAACCUCUCUUUCUUGAAGUUACCUGAGAAUGGAUCCAU
UCCCUGCAACUGAAGAUUCUAAGGAACUGGGUUUCUCAGUAUACAAUGGGAAUG
GUUGGGAGGAGGUAAAGAGUAGAAGACAGUAUCAAGAAUCCAGAGCCCAGCACC
UGUAGUCCUAACUAUUCAGAUUCCUUGAGCCCAGGAGUUUGAGUCCAGCCUGGA
CAACAUAUUGAGACCCCCAUCUCUCUAAAAAAAAAGAGAAAGAAAGAAGGAAAG
AAAAAAAGAAAGAAAGAAAGAAAGAAAGAAAGAAAGAAAGAAAGAAAGAAAGA
GAAAGAAAGAAGGAAAGAAGGAAAGAAGGAAAGAAAGAAAGAAAGAGAAAGAA
AGAAAAGAAGAUUGUAGCUAGGGGGAGAGUAGGUGAAAAGAUGAACAACAUGA
CCGGGAAGAUUUCCUAAUCUCACCACAGCCUGGCUCUACCUUAAGUCUUUAAUA
AAAGCUUGACUGAAGGUACCAAGGUGUGCUGAAGUGGAAGCAAAGUUCUCCAAA
GUCCAGCAUGGUAGACAUCAGUGGUGGUAACCAAGGACAGACCCCAAGGCAAGG
UGAACCUCAAAAAUGGAACCUCAAGUCUAUGCAGUCCAGCUGCCCUCCCCACCAG
AAAGUCCUUGUUCCAGCCCAACAUCAGUGCCUCUGAGUUUGUUUACUAGAAACA
AAGGAAGAAUUUCCUUGUAAAAAUAUAGACAGAGUAGUCCCUGGCUUUCUCCUC
UUGCAGGAAGGAUGGAUUCUCCCAUUCCAUACCAUCUUUCCCCCACACUGGCCCC
AGAAAUACUUAAUUCAACUAUGUGAAAAUAAAGAUUGUUUUUGGUUUGAGGGC
AUAGGGAUCCAUUUAUCCUUAUUCUUUAUGAGGCACUAAAUUAGCUUUGUAUGU
UAUUAAAUGUGUCUCGUCAAUGCUGUUGGCAUUGUUUCAUUUUAACUUCUAUUU
GGUAAUGUUCAAUUUUCUGAAUGUGUGGAUCACUUAGCAUGAGCAGAAAACCCU
GCAAAAGUGGUGGAGGUAAGGAAAGGUUGUAAUAAUGGUUACAAAGCAGGAGC
AGCAUAAGGCUACCUAGGUGGUACGGUUUGAGUUGUUUUGGGAAAGCCCUUCUU
AAUCACCUCUCUCUAGGACACCAC
```

The free energy of the thermodynamic ensemble is **–426.75** kcal/mol.

The frequency of the MFE structure in the ensemble is **0.00** %.

The ensemble diversity is **374.63**.

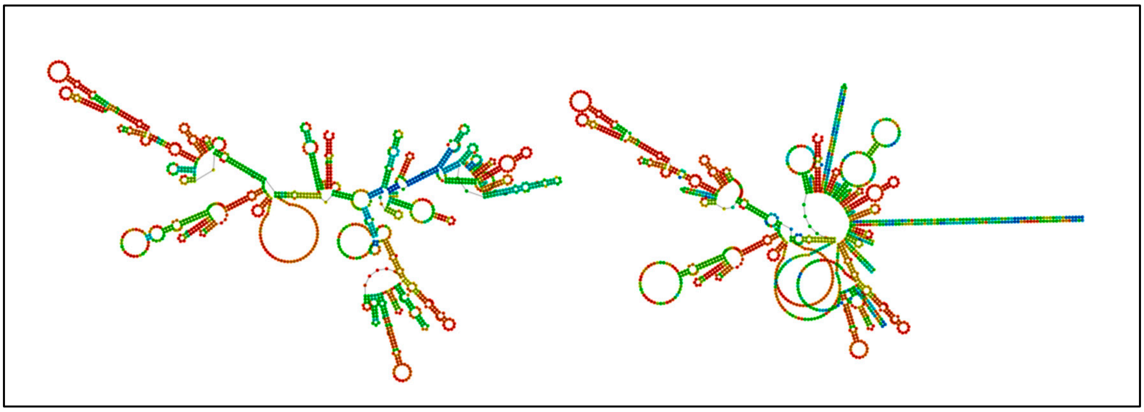

MFE secondary structure and Centroid secondary structure, respectively.

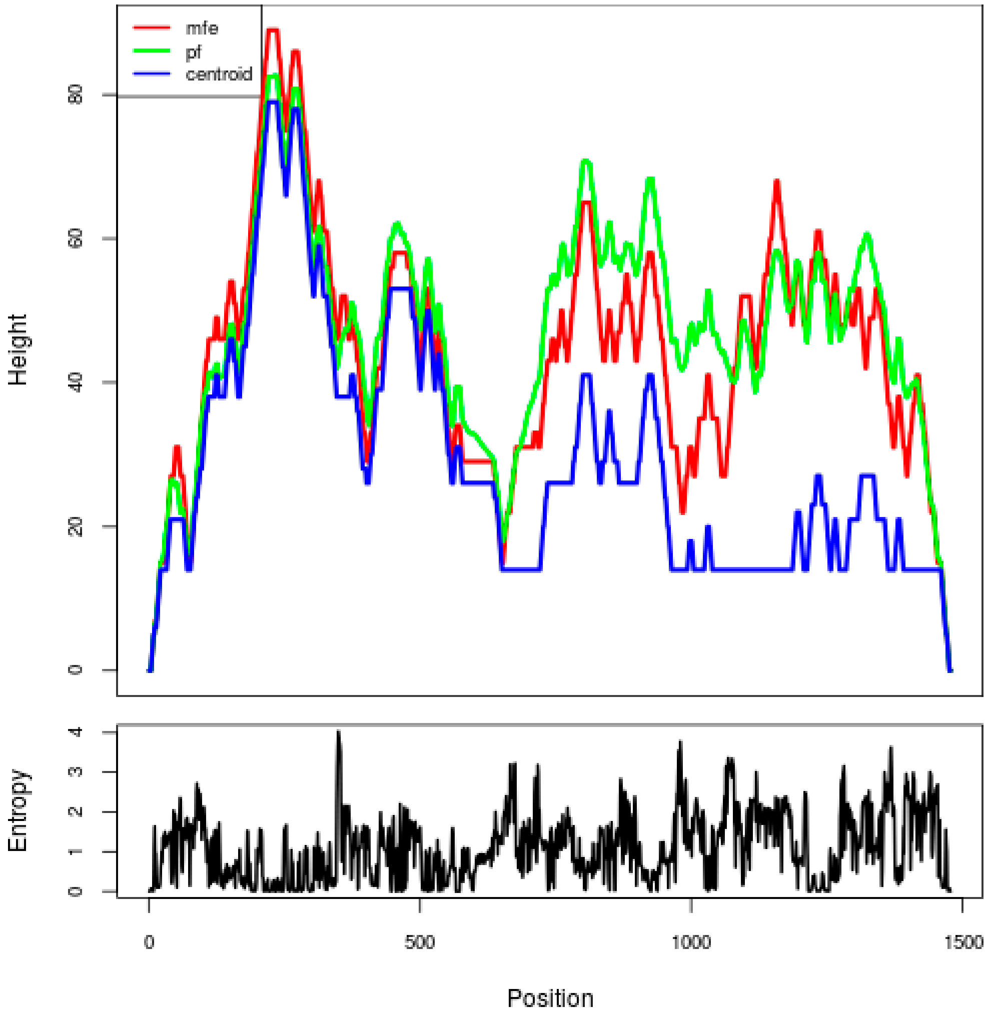

>ENST00000657602.1 lncRNA

```
CUGGCACCGACUGCCCGUUAGAGAAGGCCUGUGGUGGGCAGGCAUGUCCAGGCAC
UGGGACUCUAGCUGUGCAAGACUGUCCUGGUGCCAGGACUCUUGCUGUGCACAG
CCAUGGGCUGGGGCUGCCGGGAACAGCGUAGAUCCCGAAGGUGCUGCAGCUAGA
GCCUGUCGGUUAACUGCACUUCUUGCAGCCAAAAAUGUGUUUACUCUUGAAGGG
GGAGCCGAGGCUGCUACAGAGCAAUUAAGCCAAUGAGAAAUCAGACCCACACCC
CAAUUCUGAUGUAACAGCCUUGGGAAAGAGGUUGCAGUGAAAAGCUGGUCCUGC
UGUGGUGGAGAGAAUGGAGGAAAGAUAAUAAAAGGCCAAACCUUUGCUCCAACU
UUCUCCUUAGCUUCCCUUUGGAUCUGGAAAGCUGGGGACCCACACGGCAGAGCC
AUGGUACUGGAGGAGCCAUUAACAAAGCUUUCAAUAAACCUCUCUUUCUUGAAG
UUACCUGAGAAUGGAUCCAUUCCCUGCAACUGAAGAUUCUAAGGAACUGGGUUU
CUCAGUAUACAAUGGGAAUGGUUGGGAGGAGGUAAAGAGUAGAAGACAGUAUC
AAGAAUCCAGAGCCCAGCACCUGUAGUCCUAACUAUUCAGAUUCCUUGAGCCCA
GGAGUUUGAGUCCAGCCUGGACAACAUAUUGAGACCCCCAUCUCUCUAAAAAAA
AAGAGAAAGAAAGAAGGAAAGAAAAAAAGAAAGAAAGAAAGAAAGAAAGAAAG
AAAGAAAGAAAGAAAGAAAGAGAAAGAAAGAAGGAAAGAAGGAAAGAAGGAAA
GAAAGAAAGAAAGAGAAAGAAAGAAAAGAAGAUUGUAGCUAGGGGGAGAGUAG
GUGAAAAGAUGAACAACAUGACCGGGAAGAUUUCCUAAUCUCACCACAGCCUGG
CUCUACCUUAAGUCUUUAAUAAAAGCUUGACUGAAGGUACCAAGGUGUGCUGAA
GUGGAAGCAAAGUUCUCCAAAGUCCAGCAUGGUAGACAUCAGUGGUGGUAACCA
AGGACAGACCCCAAGGCAAGGUGAACCUCAAAAAUGGAACCUCAAGUCUAUGCA
GUCCAGCUGCCCUCCCCACCAGAAAGUCCUUGUUCCAGCCCAACAUCAGUGCCUC
UGAGUUUGUUUACUAGAAACAAAGGAAGAAUUUCCUUGUAAAAAUAUAGACAG
AGUAGUCCCUGGCUUUCUCCUCUUGCAGGAAGGAUGGAUUCUCCCAUUCCAUAC
CAUCUUUCCCCCACACUGGCCCCAGAAAUACUUAAUUCAACUAUGUGAAAAUAA
AGAUUGUUUUUGGUUUGAGGGCAUAGGGAUCCAUUUAUCCUUAUUCUUUAUGA
GGCACUAAAUUAGCUUUGUAUGUUAUUAAAUGUGUCUCGUCAAUGCUGUUGGCA
UUGUUUCAUUUUAACUUC
```

The free energy of the thermodynamic ensemble is **–440.60** kcal/mol.

The frequency of the MFE structure in the ensemble is **0.00** %.

The ensemble diversity is **342.26**.

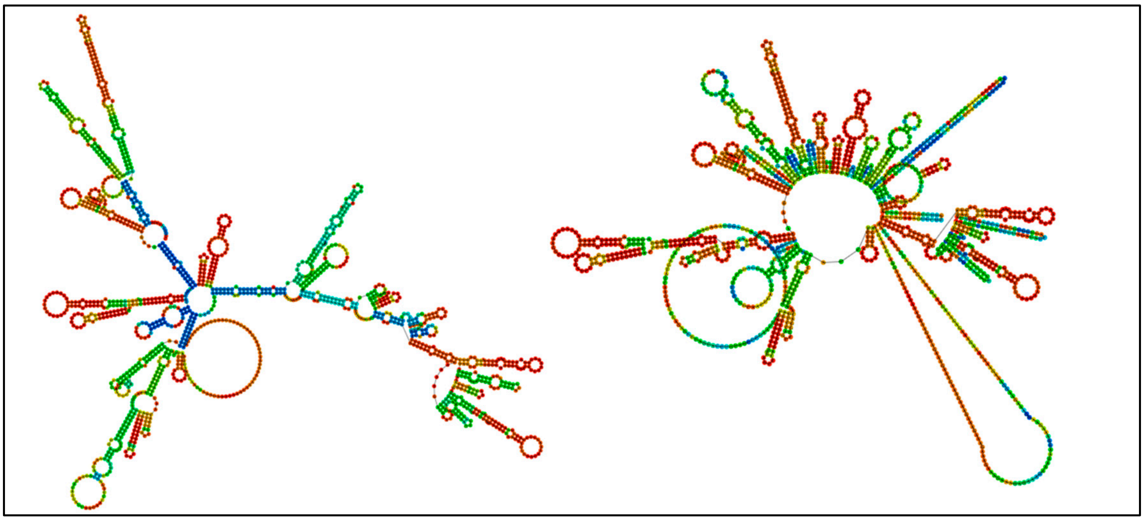

MFE secondary structure and Centroid secondary structure, respectively.

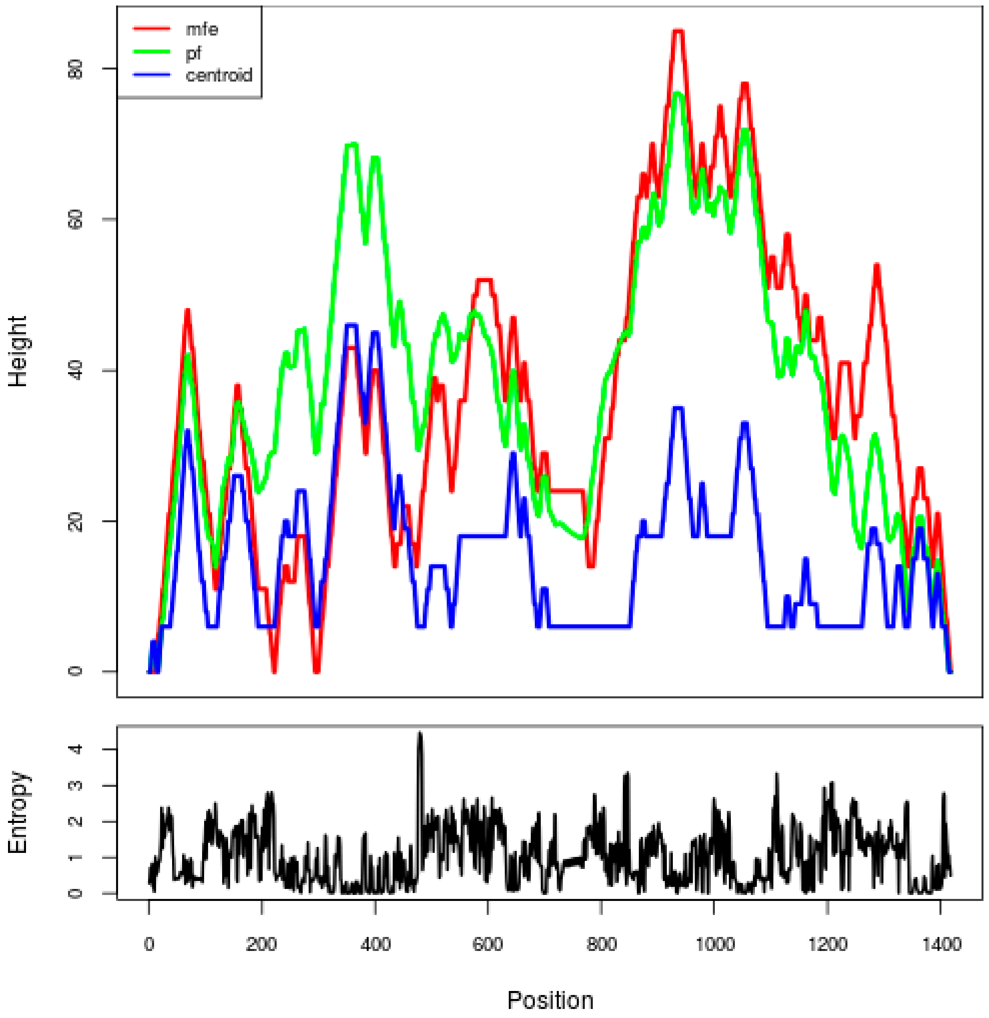

>ENST00000443364.8 lncRNA

```
AGACUUUCUCUCUCUUCUUCCUGGAUCUUUAGGAGCAAUUAAGCCAAUGAGAAA
UCAGACCCACACCCCAAUUCUGAUGUAACAGCCUUGGGAAAGAGGUUGCAGUGA
AAAGCUGGUCCUGCUGUGGUGGAGAGAAUGGAGGAAAGAUAAUAAAAGGCCAAA
CCUUUGCUCCAACUUUCUCCUUAGCUUCCCUUUGGAUCUGGAAAGCUGGGGACCC
ACACGGCAGAGCCAUGGUACUGGAGGAGCCAUUAACAACGAGGCUCCUUCUGUA
AGGUUGCCUGGAUACUGCAUCUGGCUGACAGAGUCCCCAGACAGAACUUGAAUA
GGCACUGCCAGGGUGUGUACUUCUAUGACUCUCCUUUUGCAAUGGGCCCACACC
ACCCACCUCAUUUUUCUAAUAAGAGCUUUCAAUAAACCUCUCUUUCUUGAAGUU
ACCUGAGAAUGGAUCCAUUCCCUGCAACUGAAGAUUCUAAGGAACUGGGUUUCU
CAGUAUACAAUGGGAAUGGUUGGGAGGAGGUAAAGAGUAGAAGACAGUAUCAA
GAAUCCAGAGCCCAGCACCUGUAGUCCUAACUAUUCAGAUUCCUUGAGCCCAGG
AGUUUGAGUCCAGCCUGGACAACAUAUUGAGACCCCCAUCUCUCUAAAAAAAAA
GAGAAAGAAAGAAGGAAAGAAAAAAAGAAAGAAAGAAAGAAAGAAAGAAAGAA
AGAAAGAAAGAAAGAAAGAGAAAGAAAGAAGGAAAGAAGGAAAGAAGGAAAGA
AAGAAAGAAAGAGAAAGAAAGAAAAGAAGAUUGUAGCUAGGGGGAGAGUAGGU
GAAAAGAUGAACAACAUGACCGGGAAGAUUUCCUAAUCUCACCACAGCCUGGCU
CUACCUUAAGUCUUUAAUAAAAGCUUGACUGAAGGUACCAAGGUGUGCUGAAGU
GGAAGCAAAGUUCUCCAAAGUCCAGCAUGGUAGACAUCAGUGGUGGUAACCAAG
GACAGACCCCAAGGCAAGGUGAACCUCAAAAAUGGAACCUCAAGUCUAUGCAGU
CCAGCUGCCCUCCCCACCAGAAAGUCCUUGUUCCAGCCCAACAUCAGUGCCUCUG
AGUUUGUUUACUAGAAACAAAGGAAGAAUUUCCUUGUAAAAAUAUAGACAGAG
UAGUCCCUGGCUUUCUCCUCUUGCAGGAAGGAUGGAUUCUCCCAUUCCAUACCA
UCUUUCCCCCACACUGGCCCCAGAAAUACUUAAUUCAACUAUGUGAAAAUAAAG
AUUGUUUUUGGUUUGAGGGCAUAGGGAUCCAUUUAUCCUUAUUCUUUAUGAGGC
ACUAAAUUAGCUUUGUAUGUUAUUAAAUGUGUCUCGUCAAUGCUGUUGGCAUUG
UUUCAUUUUAA
```

The free energy of the thermodynamic ensemble is **–395.83** kcal/mol.

The frequency of the MFE structure in the ensemble is **0.00** %.

The ensemble diversity is **359.75**.

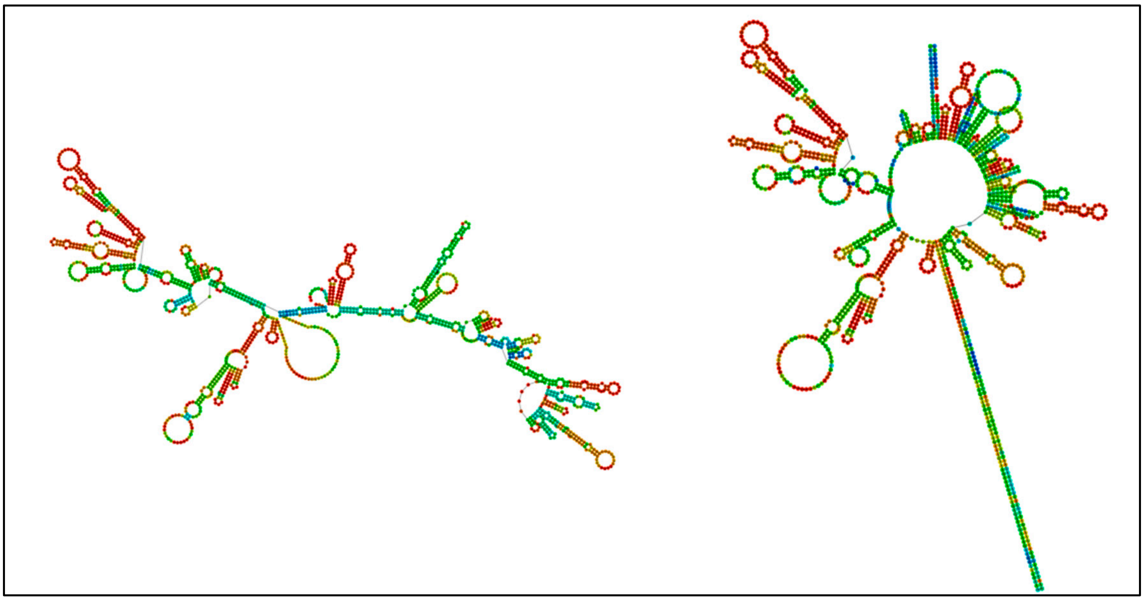

MFE secondary structure and Centroid secondary structure, respectively.

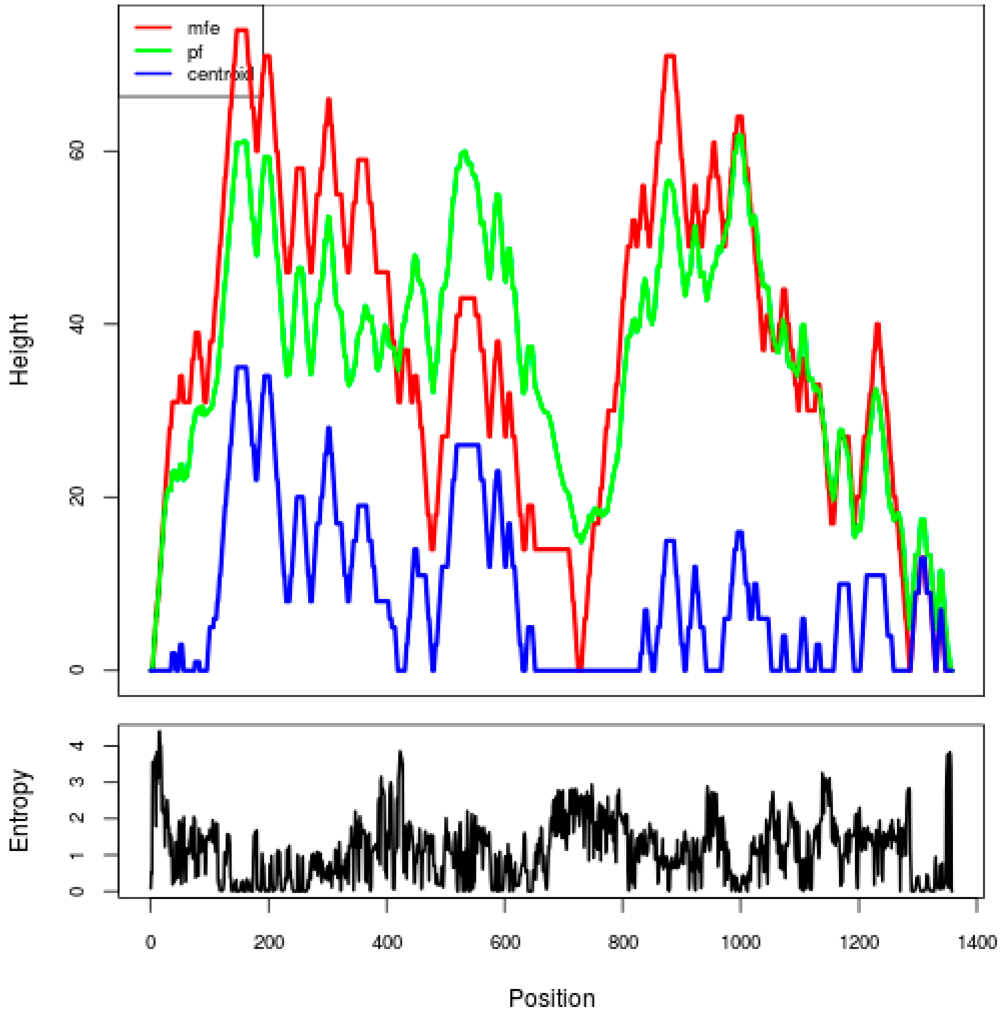

>ENST00000772304.1 lncRNA

```
CUACCUCCACCCAUUGCUUCUCUAGCAGGACUGGAAAUACCUGUCUGCAUUGGCG
AGCUCCCAAAUCUUGCCCAGGCGCCUCCCACCGUGUGCAGCUGAGGAAGGCUGUG
CUCUGAGCGUGGGGUUGCCAAGUCAGCUCCCAAGGCAGCUCUGGCACCAACGACC
CAGAGAAACAGGCCCAGAGGGGAGAAACGACAGAUAUCCUGACACGGGAGAUUG
AAAGGGAGGAAUGAAAUACAAGAUCACUUACUGGACCCAUGCAGGGUGCCAGAC
CCUGGACUAGAUGCUUUAUAACUCUUGUGUAACCUUCACAGAAACCCUGCGAAG
GGGAGGCUGAUGGACUCCUGAAGGACCGCAACCACUUCCUCGGAUCCACACCAUC
UCCAGAGUCUCCUUUGGCUGCCUAUGCAGGAAGAAAAGAGAAGCAAACAACCCA
GCUGCUGUCUCUGGGCUGCUGAACCUGGCAGUGGAGCUGCCAGAGUAGCGUAAU
CUCAGCUGGGCAGCUUCUAUGACUCUCACACUAAAUCCUGUUCUCCAGCUGCUGC
UUCUGACUCUAGAUUUCCCCUUUCCCUGAGUUAAGCACCUAAAAGGAGUUUGCU
UCCAUCUGUCCCUUCACCCUCCAAGUUUUACCUUCUCCUUUGCCAUCUUGGGAUU
GCUCUUUCUUCCAUAUCCCCAGGUGUCUCUCGGUCCUGCCCUGAAGCCACACACC
CUACUCCUUGUAACUCCAGAAGAGCUGACACAUUCAACCCCUAAGAAAGGGUCU
UACUGGCCCAGACCCAGUGGGGCAGGGGAACAGAGGUGUAGGGGACAUUCCUGU
GCACGAGCUGGCAGCUUCACUCACCCUCCUGGAGCAGCAGGAGAAGAAGCAGCCA
AGGAAAGGCACACAUGUCAGCAGCCCCCAGCCCCAGAGGUGUGAUUCAGUCAGU
CAGUCCCCAGGACUGUGCAGAAGACUGCAUUAGGAGGCUCCUAGACACAAAGAG
CCUGACUGAUGGUCCUGAGAAAAGGGAAAUAAUUUUCCCCUGACUACAGCCCUC
UGACGUCCUCUGUCCUCACUAAGCCCCUCCCUAUGUAUCAUGGCUCUCUUAGUUC
UCUUUCAGAUCCUGAUUGUCAUGACAGUUUACUCACUGACUCAUGAUGCCAUCC
ACCAAACUCAGCCCAAAUCCCACUUCCUAAAUGAAGUCUUUCAAGACUUCCUACC
CGCCCCAGGUCCCCGCACGUACUGCAUGCCCCGAACUCGUAGAACAUUCCCUAUC
CUGUUAUUUGGCUCCCAGGAUAUUCUUCCCUAGCUCUCUCUGGGUAAUUCCUAU
GUUGCUGUGAUCAUUUGAUCUUAUAAACAUCUAUAUCUCCCCAGACA
```

The free energy of the thermodynamic ensemble is **–448.96** kcal/mol.

The frequency of the MFE structure in the ensemble is **0.00** %.

The ensemble diversity is **298.78**.

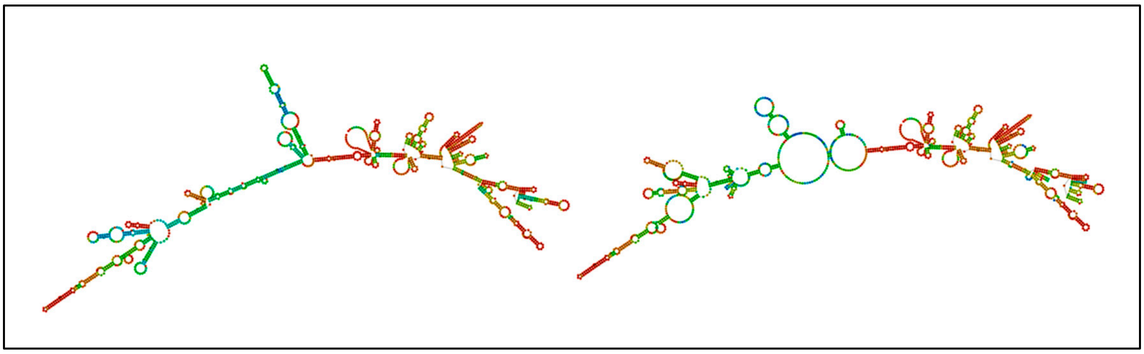

MFE secondary structure and Centroid secondary structure, respectively.

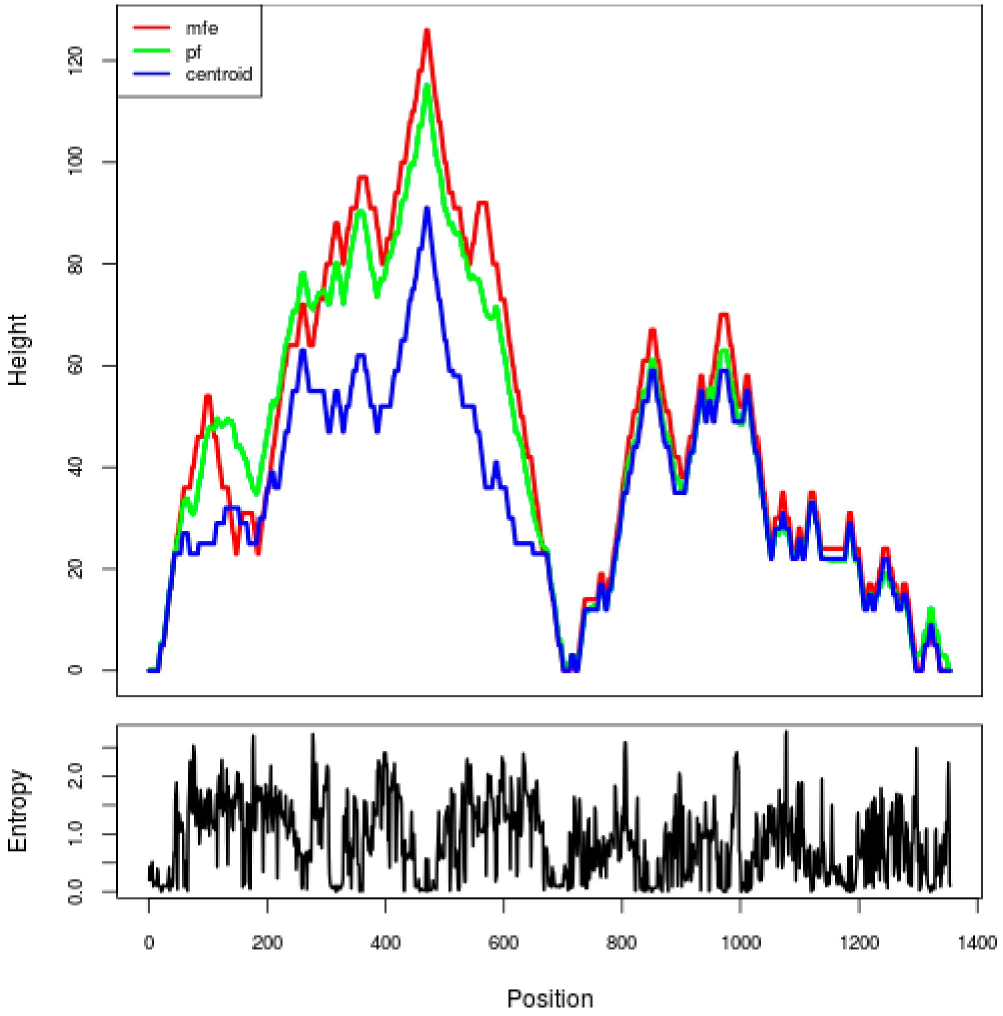

>ENST00000772287.1 lncRNA

```
GACUUAGCAGGGAGCUCUGGCACCGACUGCCCGUUAGAGAAGGCCUGUGGUGGG
CAGGCAUGUCCAGGCACUGGGACUCUAGCUGUGCAAGACUGUCCUGGUGCCAGG
ACUCUUGCUGUGCACAGCCAUGGGCUGGGGCUGCCGGGAACAGCGUAGAUCCCG
AAGGUGCUGCAGCUAGAGCCUGUCGGUUAACUGCACUUCUUGCAGCCAAAAAUG
UGUUUACUCUUGAAGGGGGAGCCGAGGCUGCUACACUUCCCUUUGGAUCUGGAA
AGCUGGGGACCCACACGGCAGAGCCAUGGUACUGGAGGAGCCAUUAACAACGAG
GCUCCUUCUGUAAGGUUGCCUGGAUACUGCAUCUGGCUGACAGAGUCCCCAGAC
AGAACUUGAAUAGGCACUGCCAGGGUGUGUACUUCUAUGACUCUCCUUUUGCAA
UGGGCCCACACCACCCACCUCAUUUUUCUAAUAAGAGCUUUCAAUAAACCUCUC
UUUCUUGAAGUUACCUGAGAAUGGAUCCAUUCCCUGCAACUGAAGAUUCUAAGG
AACUGGGUUUCUCAGUAUACAAUGGGAAUGGUUGGGAGGAGGUAAAGAGUAGA
AGACAGUAUCAAGAAUCCAGAGCCCAGCACCUGUAGUCCUAACUAUUCAGAUUC
CUUGAGCCCAGGAGUUUGAGUCCAGCCUGGACAACAUAUUGAGACCCCCAUCUC
UCUAAAAAAAAAGAGAAAGAAAGAAGGAAAGAAAAAAAGAAAGAAAGAAAGAA
AGAAAGAAAGAAAGAAAGAAAGAAAGAAAGAGAAAGAAAGAAGGAAAGAAGGA
AAGAAGGAAAGAAAGAAAGAAAGAGAAAGAAAGAAAAGAAGAUUGUAGCUAGG
GGGAGAGUAGGUGAAAAGAUGAACAACAUGACCGGGAAGAUUUCCUAAUCUCAC
CACAGCCUGGCUCUACCUUAAGUCUUUAAUAAAAGCUUGACUGAAGGUACCAAG
GUGUGCUGAAGUGGAAGCAAAGUUCUCCAAAGUCCAGCAUGGUAGACAUCAGUG
GUGGUAACCAAGGACAGACCCCAAGGCAAGGUGAACCUCAAAAAUGGAACCUCA
AGUCUAUGCAGUCCAGCUGCCCUCCCCACCAGAAAGUCCUUGUUCCAGCCCAACA
UCAGUGCCUCUGAGUUUGUUUACUAGAAACAAAGGAAGAAUUUCCUUGUAAAAA
UAUAGACAGAGUAGUCCCUGGCUUUCUCCUCUUGCAGGAAGGAUGGAUUCUCCC
AUUCCAUACCAUCUUUCCCCCACACUGGCCCCAGAAAUACUUAAUUCAACUAUG
UGAAAAUAAAGAUUGUUUUUGGUUUGAGGGCA
```

The free energy of the thermodynamic ensemble is **–416.48** kcal/mol.

The frequency of the MFE structure in the ensemble is **0.00** %.

The ensemble diversity is **321.42**.

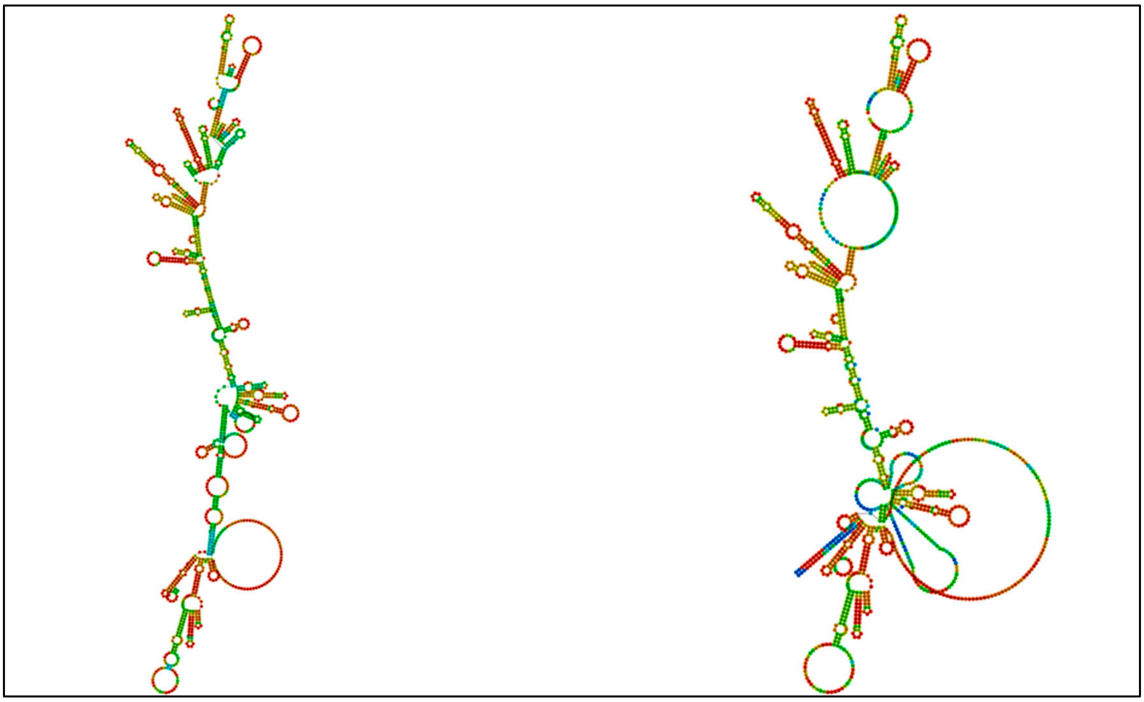

MFE secondary structure and Centroid secondary structure, respectively.

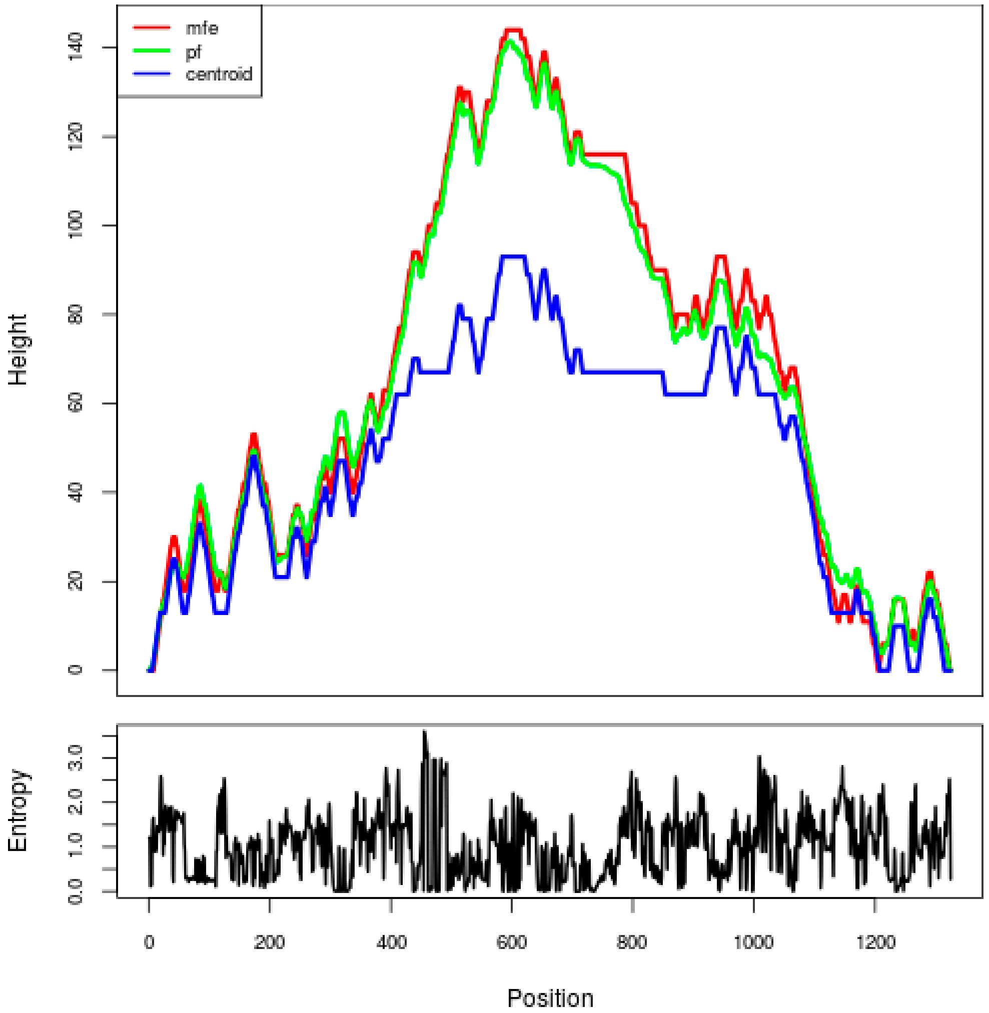

>ENST00000772286.1 lncRNA

```
GAUGCGUCUCACAGUCUCAGCUGACUUAGCAGGGAGCUCUGGCACCGACUGCCCG
UUAGAGAAGGCCUGUGGUGGGCAGGCAUGUCCAGGCACUGGGACUCUAGCUGUG
CAAGACUGUCCUGGUGCCAGGACUCUUGCUGUGCACAGCCAUGGGCUGGGGCUG
CCGGGAACAGCGUAGAUCCCGAAGGUGCUGCAGCUAGAGCCUGUCGGUUAACUG
CACUUCUUGCAGCCAAAAAUGUGUUUACUCUUGAAGGGGGAGCCGAGGCUGCUA
CACUUCCCUUUGGAUCUGGAAAGCUGGGGACCCACACGGCAGAGCCAUGGUACU
GGAGGAGCCAUUAACAAAGCUUUCAAUAAACCUCUCUUUCUUGAAGUUACCUGA
GAAUGGAUCCAUUCCCUGCAACUGAAGAUUCUAAGGAACUGGGUUUCUCAGUAU
ACAAUGGGAAUGGUUGGGAGGAGGUAAAGAGUAGAAGACAGUAUCAAGAAUCC
AGAGCCCAGCACCUGUAGUCCUAACUAUUCAGAUUCCUUGAGCCCAGGAGUUUG
AGUCCAGCCUGGACAACAUAUUGAGACCCCCAUCUCUCUAAAAAAAAAGAGAAA
GAAAGAAGGAAAGAAAAAAAGAAAGAAAGAAAGAAAGAAAGAAAGAAAGAAAG
AAAGAAAGAAAGAGAAAGAAAGAAGGAAAGAAGGAAAGAAGGAAAGAAAGAAA
GAAAGAGAAAGAAAGAAAAGAAGAUUGUAGCUAGGGGGAGAGUAGGUGAAAAG
AUGAACAACAUGACCGGGAAGAUUUCCUAAUCUCACCACAGCCUGGCUCUACCU
UAAGUCUUUAAUAAAAGCUUGACUGAAGGUACCAAGGUGUGCUGAAGUGGAAGC
AAAGUUCUCCAAAGUCCAGCAUGGUAGACAUCAGUGGUGGUAACCAAGGACAGA
CCCCAAGGCAAGGUGAACCUCAAAAAUGGAACCUCAAGUCUAUGCAGUCCAGCU
GCCCUCCCCACCAGAAAGUCCUUGUUCCAGCCCAACAUCAGUGCCUCUGAGUUUG
UUUACUAGAAACAAAGGAAGAAUUUCCUUGUAAAAAUAUAGACAGAGUAGUCCC
UGGCUUUCUCCUCUUGCAGGAAGGAUGGAUUCUCCCAUUCCAUACCAUCUUUCC
CCCACACUGGCCCCAGAAAUACUUAAUUCAACUAUGUGAAAAUAAAGAUUGUUU
UUGGUUUGAGGGCAUAGGGAUCCAUUUAUCCUUAUUCUUUAUGAGGCACUAAAU
UAGCUUUGUAUGUUAUUAAAUGUGUCUCGUCAA
```

The free energy of the thermodynamic ensemble is **–387.07** kcal/mol.

The frequency of the MFE structure in the ensemble is **0.00** %.

The ensemble diversity is **356.03**.

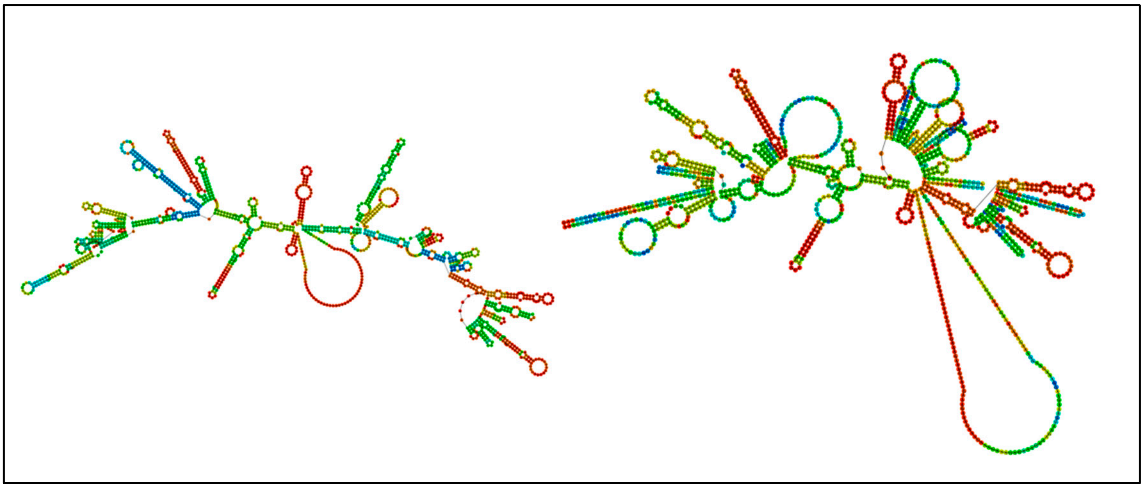

MFE secondary structure and Centroid secondary structure, respectively.

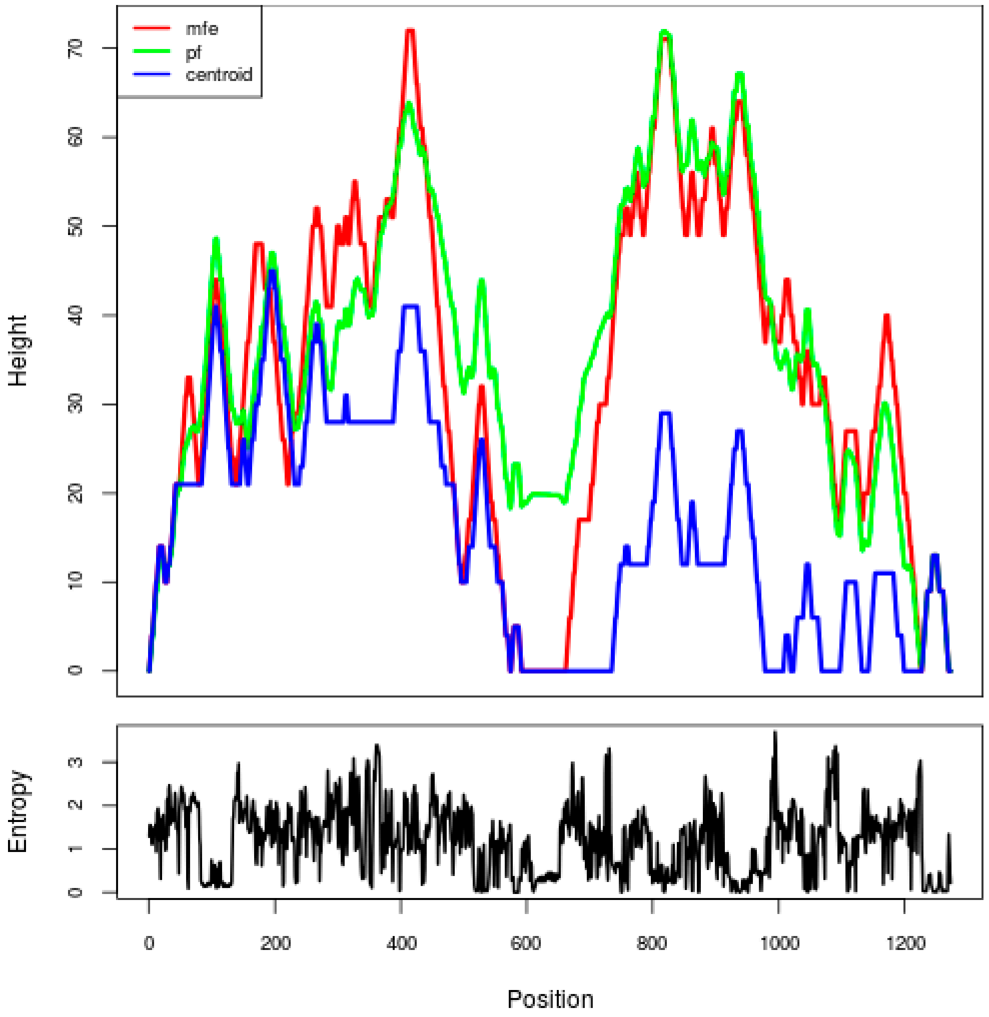

>ENST00000772299.1 lncRNA

```
AACAGCCUUGGGAAAGAGGUUGCAGUGAAAAGCUGGUCCUGCUGUGGUGGAGAG
AAUGGAGGAAAGAUAAUAAAAGGCCAAACCUUUGCUCCAACUUUCUCCUUAGCU
UCCCUUUGGAUCUGGAAAGCUGGGGACCCACACGGCAGAGCCAUGGUACUGGAG
GAGCCAUUAACAAAGCUUUCAAUAAACCUCUCUUUCUUGAAGUUACCUGAGAAU
GGAUCCAUUCCCUGCAACUGAAGAUUCUAAGGAACUGGGUUUCUCAGUAUACAA
UGGGAAUGGUUGGGAGGAGGUAAAGAGUAGAAGACAGUAUCAAGAAUCCAGAGC
CCAGCACCUGUAGUCCUAACUAUUCAGAUUCCUUGAGCCCAGGAGUUUGAGUCC
AGCCUGGACAACAUAUUGAGACCCCCAUCUCUCUAAAAAAAAAGAGAAAGAAAG
AAGGAAAGAAAAAAAGAAAGAAAGAAAGAAAGAAAGAAAGAAAGAAAGAAAGA
AAGAAAGAGAAAGAAAGAAGGAAAGAAGGAAAGAAGGAAAGAAAGAAAGAAAG
AGAAAGAAAGAAAAGAAGAUUGUAGCUAGGGGGAGAGUAGGUGAAAAGAUGAA
CAACAUGACCGGGAAGAUUUCCUAAUCUCACCACAGCCUGGCUCUACCUUAAGA
UUUGAAAACUGCCAGCCCCCACCACUGCCCCACCCCACCAGGUCAUAGUUCCUAU
UAAGUUCCAUAGGAACAUUUCUGCACUGCAUCCUGGCCAUCCUGGCUUCAGGGU
CUCCACAGAGUGAGCCCAUGUCAUACCCCUCCCCACUUCAGGAGGAACAAGACAA
AAGGGCCAGUCUUUAAUAAAAGCUUGACUGAAGGUACCAAGGUGUGCUGAAGUG
GAAGCAAAGUUCUCCAAAGUCCAGCAUGGUAGACAUCAGUGGUGGUAACCAAGG
ACAGACCCCAAGGCAAGGUGAACCUCAAAAAUGGAACCUCAAGUCUAUGCAGUC
CAGCUGCCCUCCCCACCAGAAAGUCCUUGUUCCAGCCCAACAUCAGUGCCUCUGA
GUUUGUUUACUAGAAACAAAGGAAGAAUUUCCUUGUAAAAAUAUAGACAGAGU
AGUCCCUGGCUUUCUCCUCUUGCAGGAAGGAUGGAUUCUCCCAUUCCAUACCAU
CUUUCCCCCACACUGGCCCCAGAAAUACUUAAUUCAACUAUGUGAAAAUAAAGA
UUGUUUUUGGUUUGAGGGCA
```

The free energy of the thermodynamic ensemble is **–345.83** kcal/mol.

The frequency of the MFE structure in the ensemble is **0.00** %.

The ensemble diversity is **254.85**.

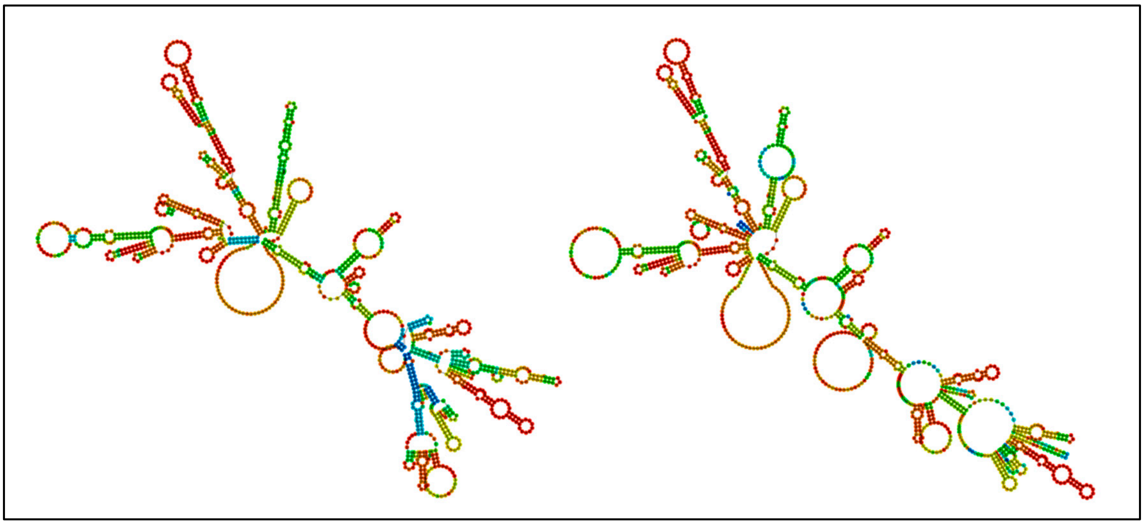

MFE secondary structure and Centroid secondary structure, respectively.

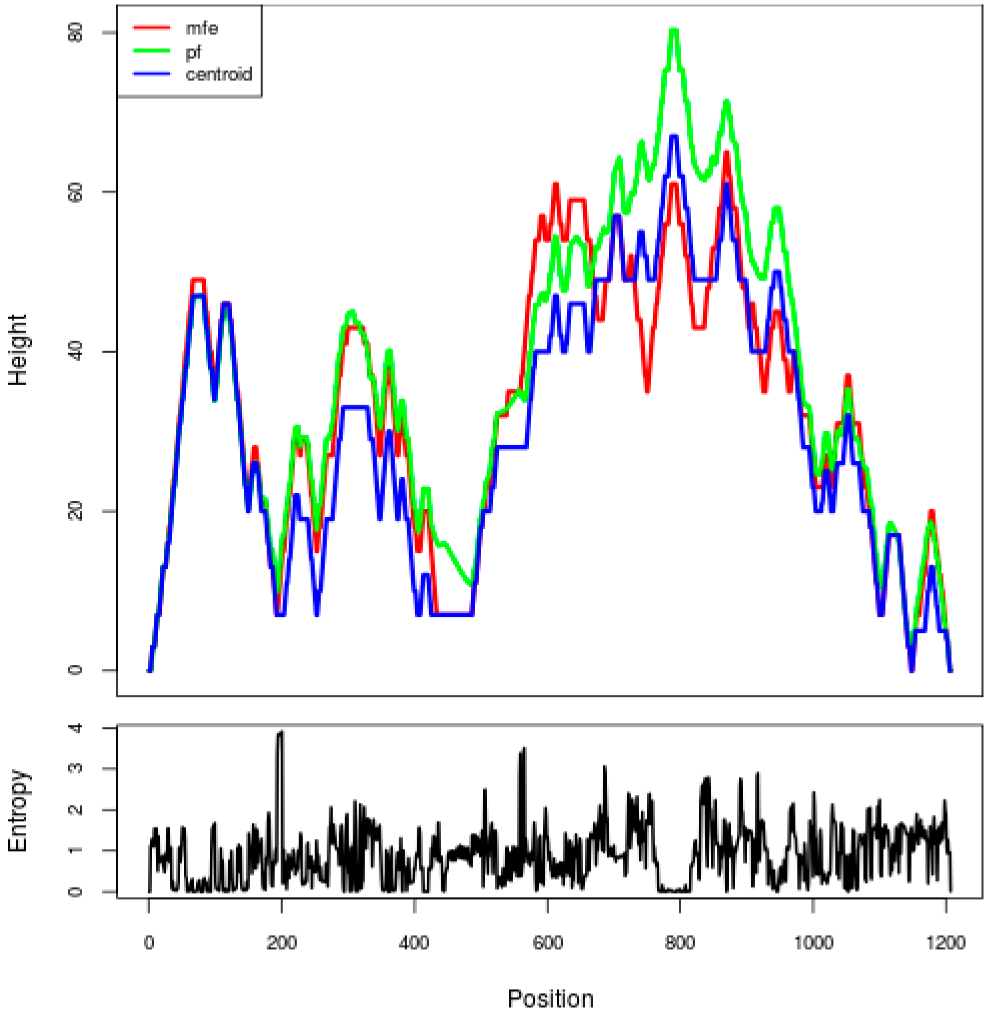

>ENST00000772284.1 lncRNA

```
GAUGUAACAGCCUUGGGAAAGAGGUUGCAGUGAAAAGCUGGUCCUGCUGUGGUG
GAGAGAAUGGAGGAAAGAUAAUAAAAGGCCAAACCUUUGCUCCAACUUUCUCCU
UAGCUUCCCUUUGGAUCUGGAAAGCUGGGGACCCACACGGCAGAGCCAUGGUAC
UGGAGGAGCCAUUAACAACGAGGCUCCUUCUGUAAGGUUGCCUGGAUACUGCAU
CUGGCUGACAGAGUCCCCAGACAGAACUUGAAUAGGCACUGCCAGGGUGUGUAC
UUCUAUGACUCUCCUUUUGCAAUGGGCCCACACCACCCACCUCAUUUUUCUAAU
AAGGUCUCUGUGACAGAGAUGAGGUGGGGCAUACACUAUAUUCCUUCUUUCCUG
GCCUUGAAUGUGGAGUCAUUUCAUUUAUGUGAAGAAAUGCUGACGUUGCAGACA
GCCCCAUCUUCCCAGUGUUCCCCCAAUCUUUCUGGGGAACUUCCAUUUGCUAGGG
AGAGCUGCAUGCCUUCCUAUCUGGGACACUGGACAUAGAUUUUCCUGGCUCUGG
GACUCUCUGGGCCAAGGUCUGAAUGAGGAUAAGGUGAAGGAGGCAUCACUUUUC
AAAAUUUAAGGGAGUGCUACAGUUUACUCUCUCUUCUUGGGCUGGGACAUCUAU
CUGCUGCUGCCUUUGGAAAUUGGAGCUCCUGGUCCUCAGGCCUUUAGACUCUAG
GACUUGACACCAGCAGUUCCCCACCCCAUCCCCAACCCCAAGCUCCAGUUCUCAG
ACUUUUGGUCUUGGACCGUUACACCAUCGGCUCUCCUGGUUCUCAGGCCUCUGG
ACUUGAACUGAAUUACACCACUGGCUUACCUGGUUCUCCAGCUUGCAAACAGCA
UAUCAUGGAAUUUUUUGGCCUCUAUAGUCACACGAGCUAAUUCUCAUAAUAAAU
CUCCUCUCAUGC
```

The free energy of the thermodynamic ensemble is **–319.10** kcal/mol.

The frequency of the MFE structure in the ensemble is **0.00** %.

The ensemble diversity is **149.78**.

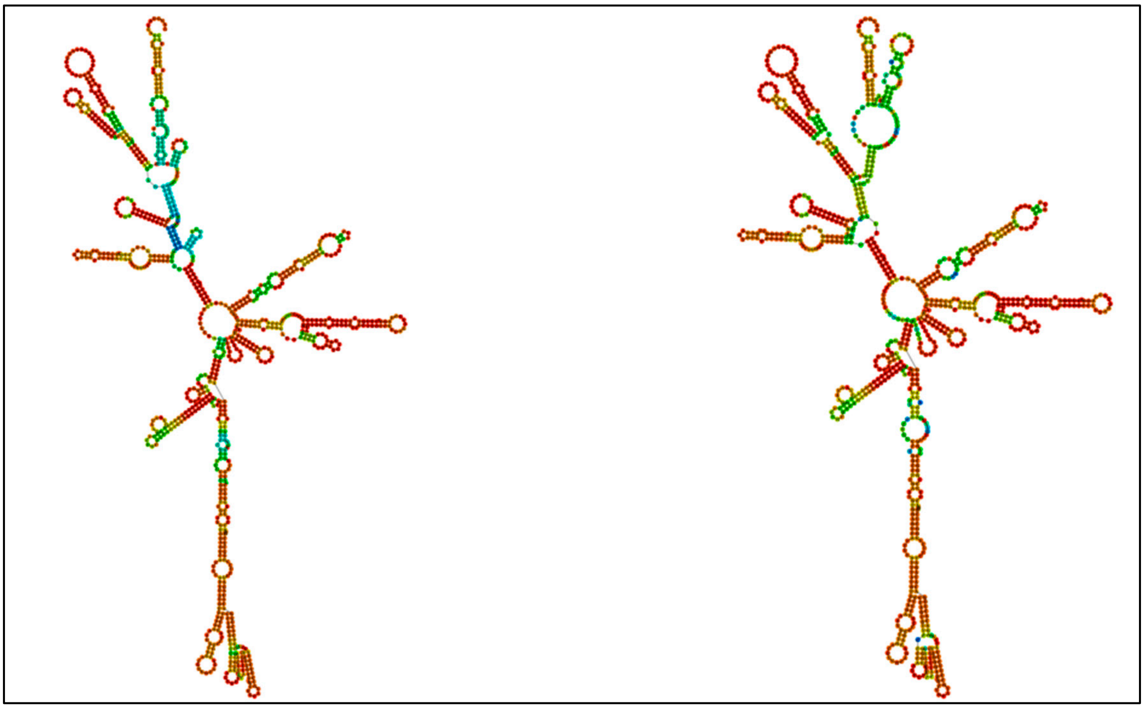

MFE secondary structure and Centroid secondary structure, respectively.

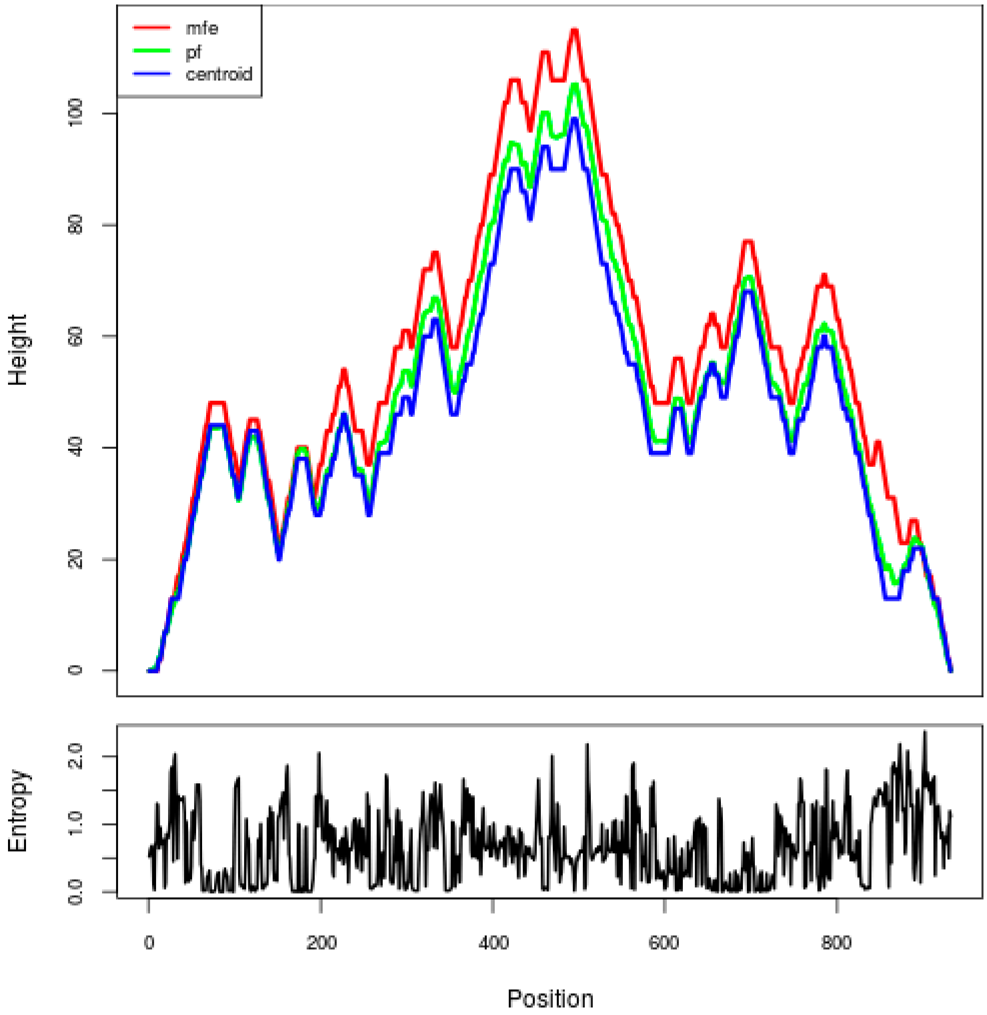

>ENST00000772305.1 lncRNA

```
AAUGGGCUCAGGGGCCUCCCUCAGGUCCCAACUCCCUCAGCCGGCUCUGGCCUGA
CCAAGAGCUCCAGACGGAGGACUGGAAAUACCUGUCUGCAUUGGCGAGCUCCCA
AAUCUUGCCCAGGCGCCUCCCACCGUGUGCAGCUGAGGAAGGCUGUGCUCUGAGC
GUGGGGUUGCCAAGUCAGCUCCCAAGGCAGCUCUGGCACCAACGACCCAGAGAAA
CAGGCCCAGAGGGGAGAAACGACAGAUAUCCUGACACGGGAGAUUGAAAGGGAG
GAAUGAAAUACAAGAUCACUUACUGGACCCAUGCAGGGUGCCAGACCCUGGA
```

The free energy of the thermodynamic ensemble is **–123.47** kcal/mol.

The frequency of the MFE structure in the ensemble is **0.01** %.

The ensemble diversity is **67.64**.

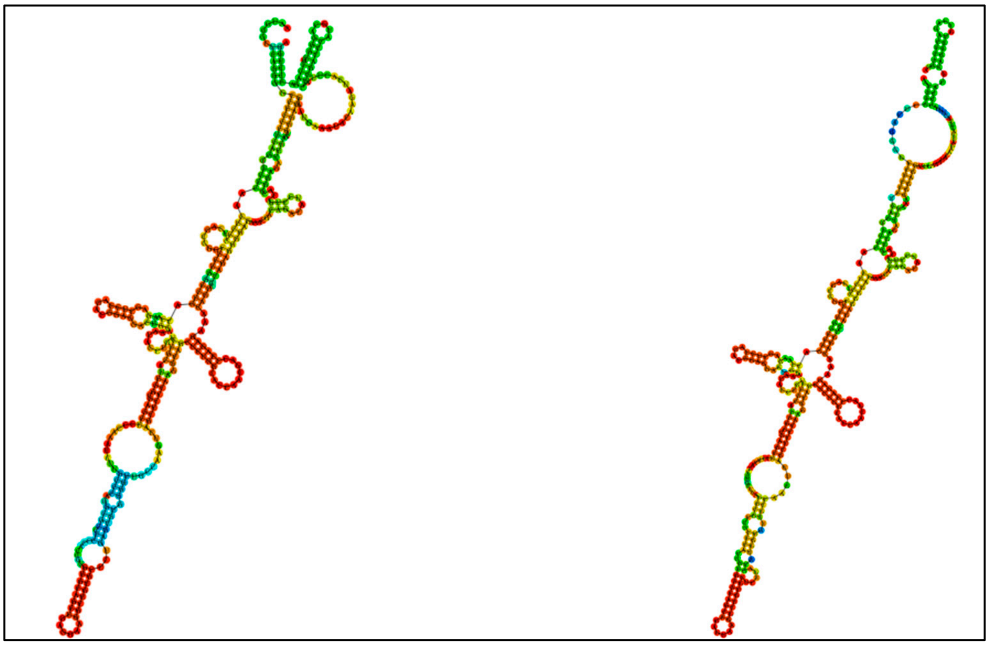

MFE secondary structure and Centroid secondary structure, respectively.

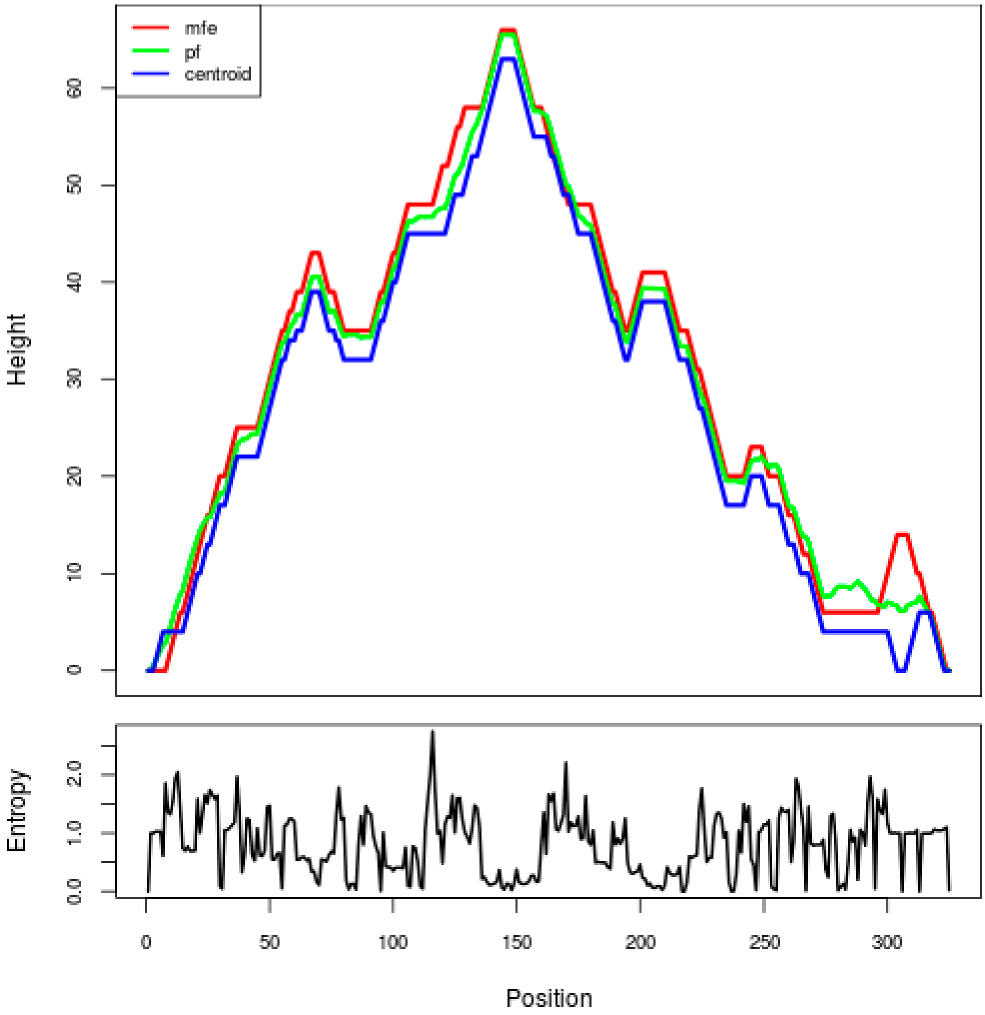

>ENST00000772307.1 lncRNA

GAGCUCUGGCACCGACUGCCCGUUAGAGAAGGCCUGUGGUGGGCAGGCAUGUCC<br>AGGCACUGGGACUCUAGCUGUGCAAGACUGUCCUGGUGCCAGGACUCUUGCUGU<br>GCACAGCCAUGGGCUGGGGCUGCCGGGAACAGCGUAGAUCCCGAAGGUGCUGCA<br>GCUAGAGCCUGUCGGUUAACUGCACUUCUUGCAGCCAAAAAUGUGUUUACUCUU<br>GAAGGGGGAGCCGAGGCUGCUACACUUCCCUUUGGAUCUGGAAAGCUGGGGACC<br>CACACGGCAGAGCCAUGGUACUGGAGGAGCCAUUAACAAGUAAGUUCCAAAUAA<br>AUCACCUCCCUCCCGAG

The free energy of the thermodynamic ensemble is **–130.93** kcal/mol.

The frequency of the MFE structure in the ensemble is **0.00** %.

The ensemble diversity is **116.66**.

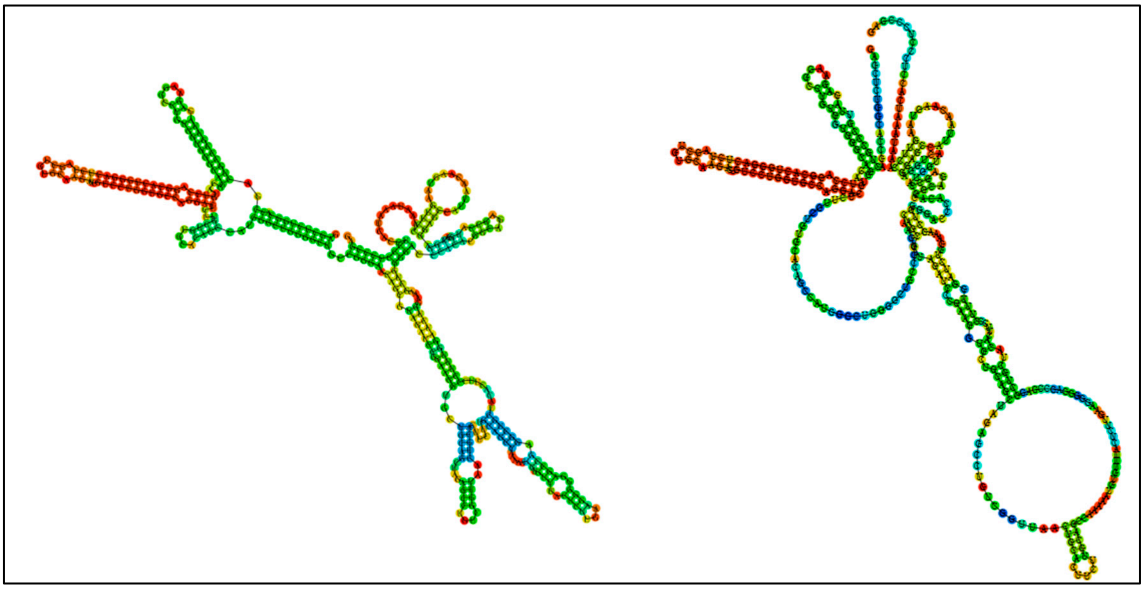

MFE secondary structure and Centroid secondary structure, respectively.

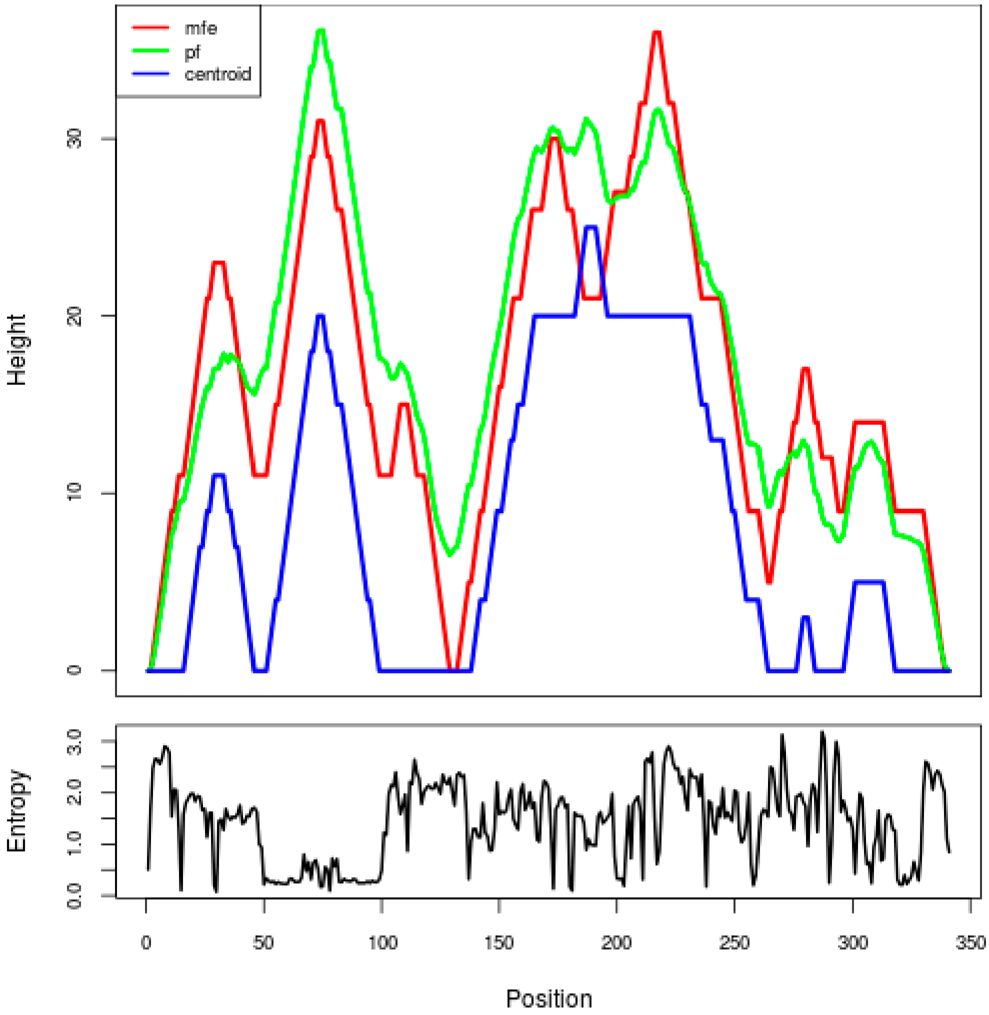

>ENST00000772288.1 lncRNA

```
AAUAAAGUAGUUGCACCAGUAAUGUUUAGACUUUCUCUCUCUUCUUCCUGGAUC
UUUAGGAGCAAUUAAGCCAAUGAGAAAUCAGACCCACACCCCAAUUCUGAUGUA
ACAGCCUUGGGAAAGAGGUUGCAGUGAAAAGCUGGUCCUGCUGUGGUGGAGAGA
AUGGAGGAAAGAUAAUAAAAGGCCAAACCUUUGCUCCAACUUUCUCCUUAGCUU
CCCUUUGGAUCUGGAAAGCUGGGGACCCACACGGCAGAGCCAUGGUACUGGAGG
AGCCAUUAACAAAUGGAGUUUCAAUCUUGUUGCCCAGGCUGGAGUGCAAUGGUG
CGAUCUCAGUUCACCGCAACCUCCGCCUCCCAG
```

The free energy of the thermodynamic ensemble is **–117.42** kcal/mol.

The frequency of the MFE structure in the ensemble is **0.00** %.

The ensemble diversity is **44.96**.

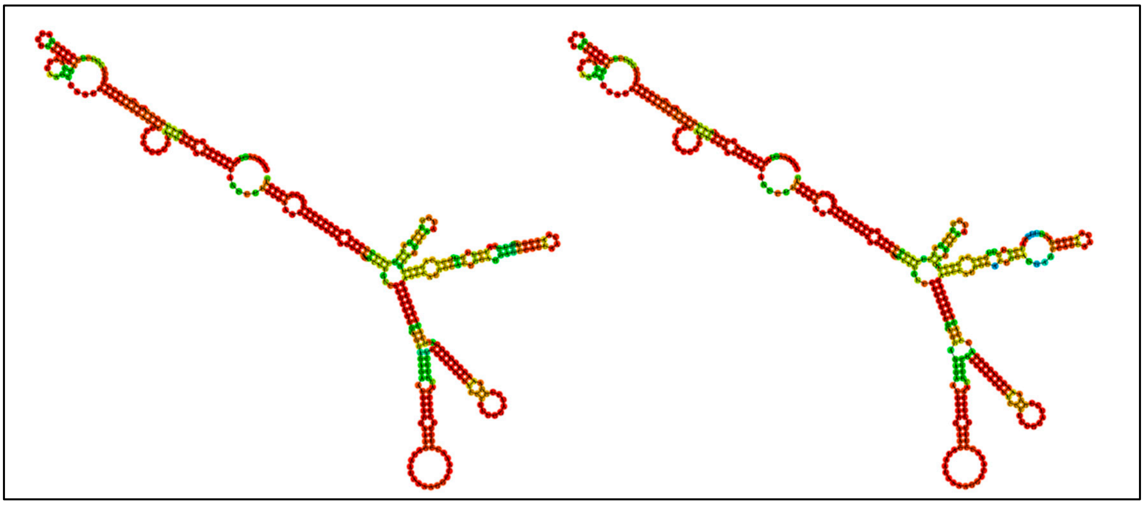

MFE secondary structure and Centroid secondary structure, respectively.

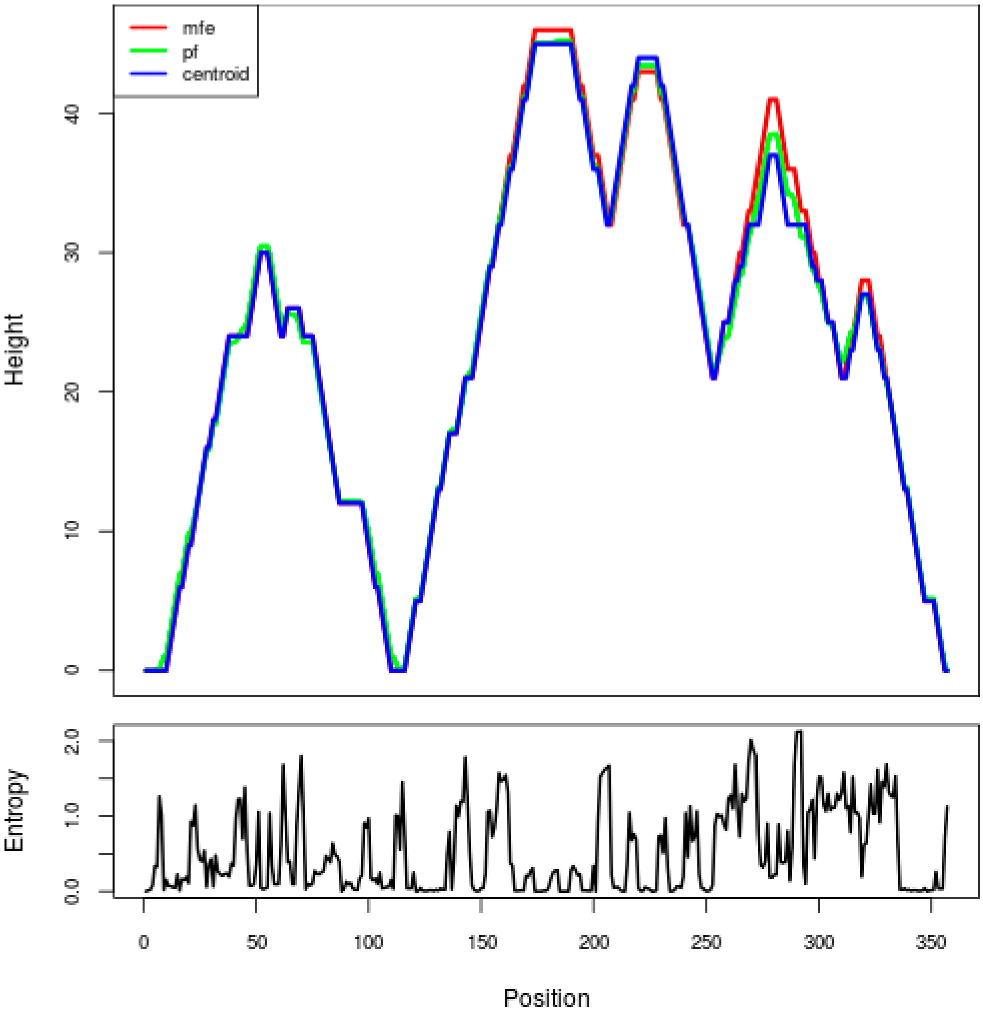

>ENST00000772292.1 lncRNA

AAUUCUGAUGUAACAGCCUUGGGAAAGAGGUUGCAGUGAAAAGCUGGUCCUGCU<br>GUGGUGGAGAGAAUGGAGGAAAGAUAAUAAAAGGCCAAACCUUUGCUCCAACUU<br>UCUCCUUAGCUUCCCUUUGGAUCUGGAAAGCUGGGGACCCACACGGCAGAGCCA<br>UGGUACUGGAGGAGCCAUUAACAACGAGGCUCCUUCUGUAAGGUUGCCUGGAUA<br>CUGCAUCUGGCUGACAGAGUCCCCAGACAGAACUUGAAUAGGCACUGCCAGGGU<br>GUGUACUUCUAUGACUCUCCUUUUGCAAUGGGCCCACACCACCCACCUCAUUUUU<br>CUAAUAAGAUGGAGUUUCAAUCUUGUUGCCCAGGCUGGAGUGCA

The free energy of the thermodynamic ensemble is **–132.32** kcal/mol.

The frequency of the MFE structure in the ensemble is **0.00** %.

The ensemble diversity is **53.89**.

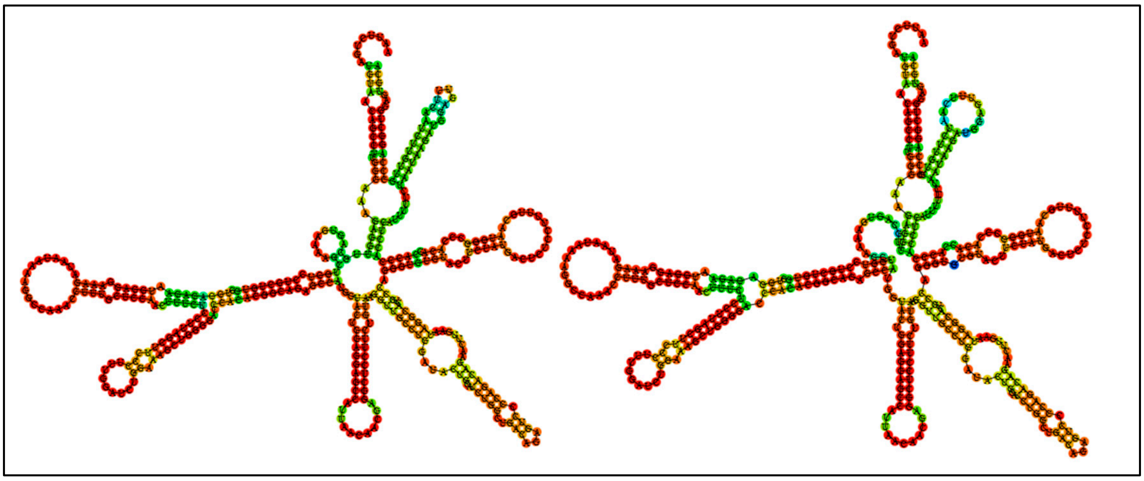

MFE secondary structure and Centroid secondary structure, respectively.

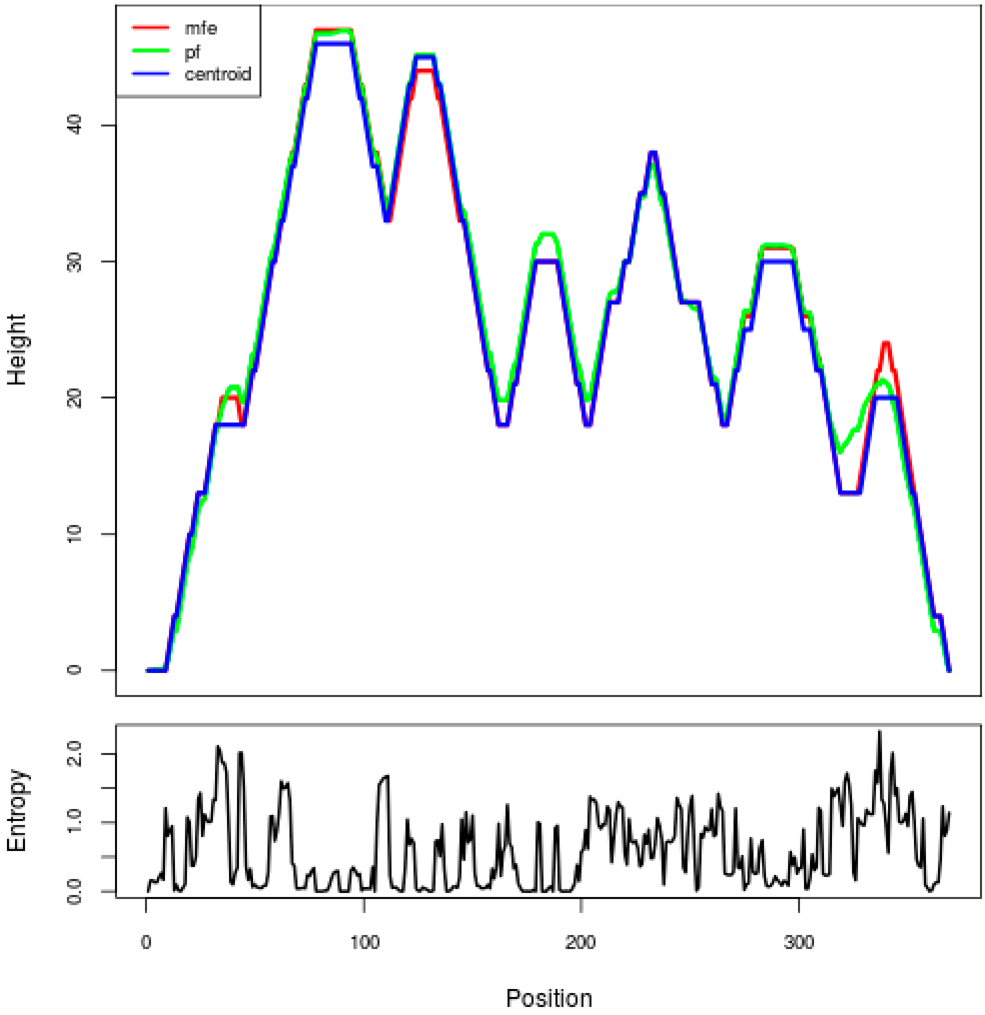

>ENST00000772289.1 lncRNA

```
GUAGUUGCACCAGUAAUGUUUAGACUUUCUCUCUCUUCUUCCUGGAUCUUUAGG
AGCAAUUAAGCCAAUGAGAAAUCAGACCCACACCCCAAUUCUGAUGUAACAGCC
UUGGGAAAGAGGUUGCAGUGAAAAGCUGGUCCUGCUGUGGUGGAGAGAAUGGAG
GAAAGAUAAUAAAAGGCCAAACCUUUGCUCCAACUUUCUCCUUAGCUUCCCUUU
GGAUCUGGAAAGCUGGGGACCCACACGGCAGAGCCAUGGUACUGGAGGAGCCAU
UAACAAUCUUUAAUAAAAGCUUGACUGAAGGUACCAAGGUGUGCUGAAGUGGAA
GCAAAGUUCUCCAAAGUCCAGCAUGGUAGACAUCAGUGGUGGUAACCAAGGACA
GACCCCAAGGCAAGAUGGAGUUUCAAUCUUGUUGC
```

The free energy of the thermodynamic ensemble is **–128.29** kcal/mol.

The frequency of the MFE structure in the ensemble is **0.00** %.

The ensemble diversity is **98.04**.

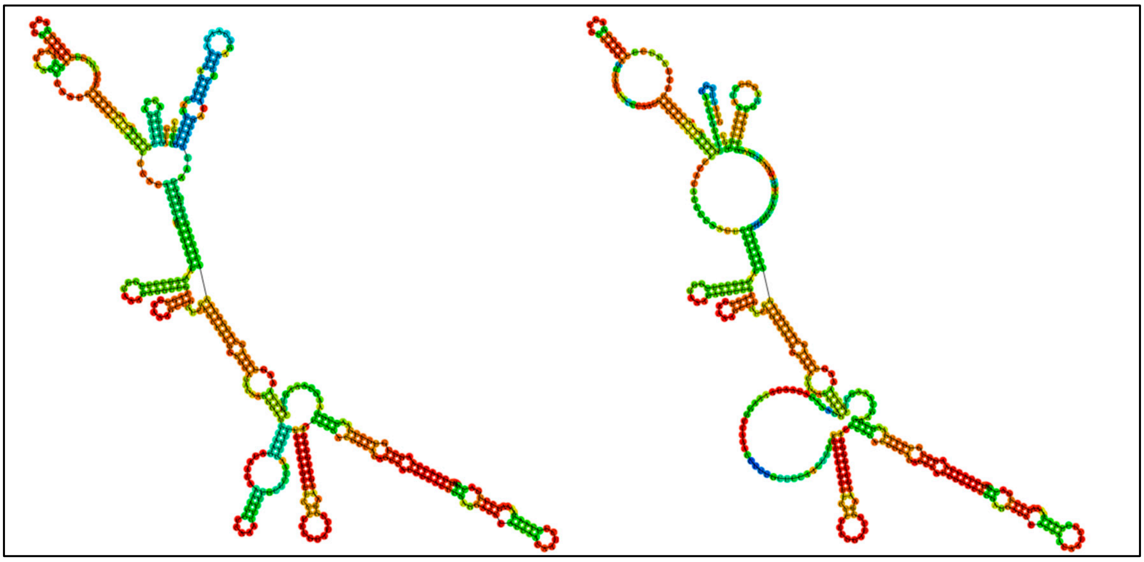

MFE secondary structure and Centroid secondary structure, respectively.

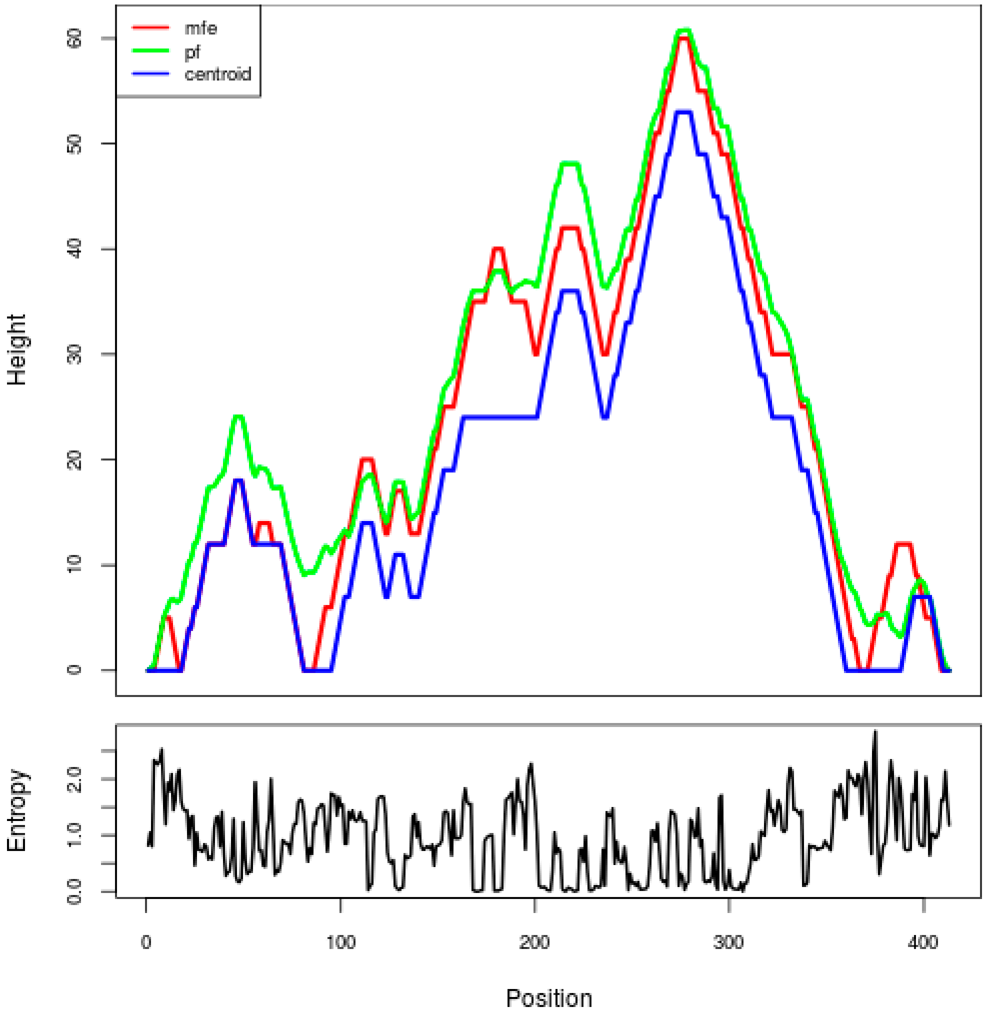

>ENST00000772309.1 lncRNA

```
AGAGGCACGGCGAGGCGGGCGGGACCCCUGAGCGGCCUGGGAGUUGUAGUUUCA
GGAGGGAAGAGCUCGGCUGCACGUGGGCUCAGGUCGCCCCCUCGUGGACCUCUGC
ACAGCCAAGCGUGGAAAGGCCGGUGGGCCGCUUCCCGCCGCCAGCAAGAGGGGCA
GCAGGGUCCGGCUCGCGCUCCAGGGCUGCGGGAGCUGGCCCCUUGCCAGGCUACU
GGACUGGAAAUACCUGUCUGCAUUGGCGAGCUCCCAAAUCUUGCCCAGGCGCCUC
CCACCGUGUGCAGCUGAGGAAGGCUGUGCUCUGAGCGUGGGGUUGCCAAGUCAG
CUCCCAAGGCAGCUCUGGCACCAACGACCCAGAGAAACAGGCCCAGAGGGGAGAA
ACGACAGAUAUCCUGACACGGGAGAUUGAAAGGGAGGAAUGAAAUACAAGAUCA
CUUACUGGACCCAUGCAGGGUGCCAGACCCUGGA
```

The free energy of the thermodynamic ensemble is **–205.82** kcal/mol.

The frequency of the MFE structure in the ensemble is **0.00** %.

The ensemble diversity is **93.08**.

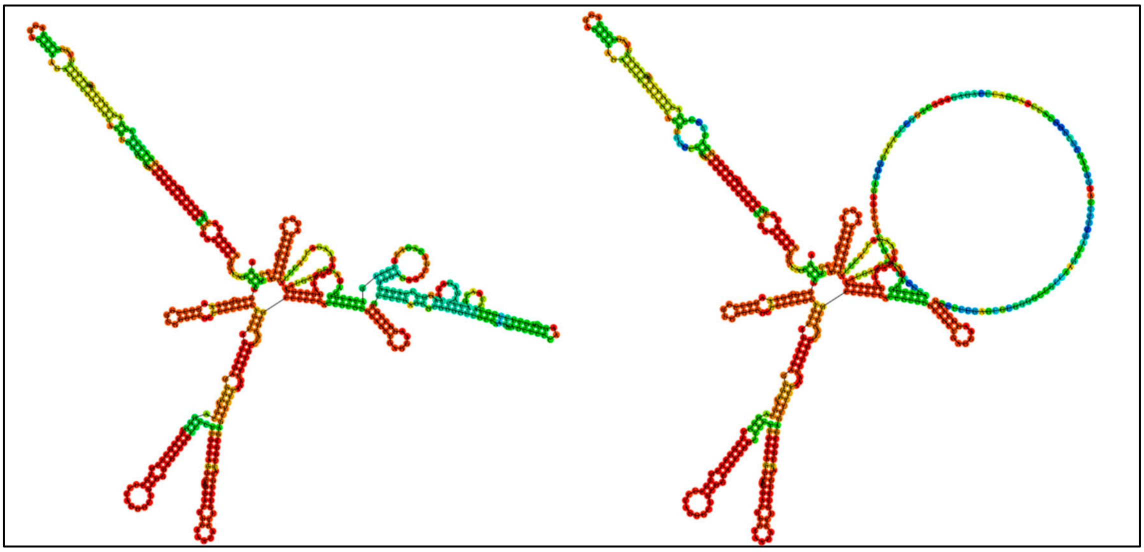

MFE secondary structure and Centroid secondary structure, respectively.

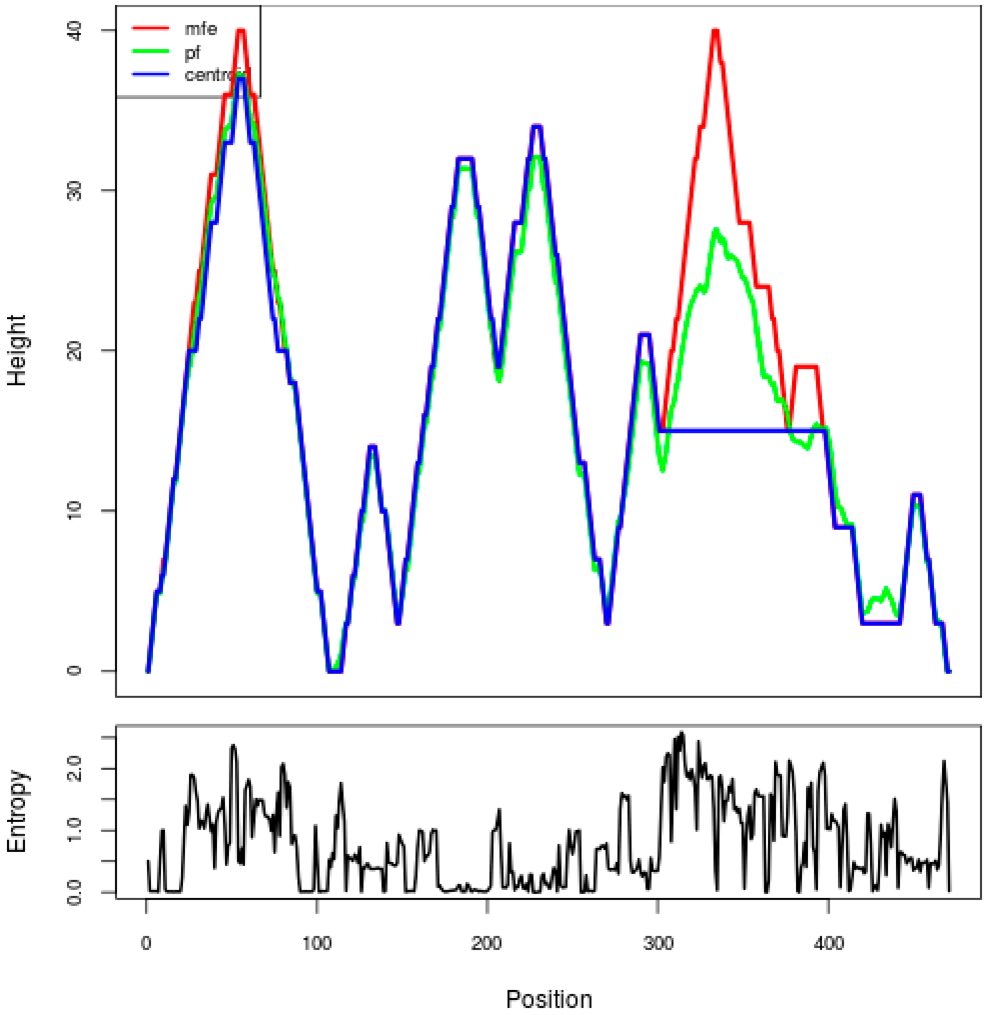

>ENST00000772303.1 lncRNA

```
CCAACUCCCUCAGCCGGCUCUGGCCUGACCAAGAGCUCCAGACGGAGCAGGACUG
GAAAUACCUGUCUGCAUUGGCGAGCUCCCAAAUCUUGCCCAGGCGCCUCCCACCG
UGUGCAGCUGAGGAAGGCUGUGCUCUGAGCGUGGGGUUGCCAAGUCAGCUCCCA
AGGCAGCUCUGGCACCAACGACCCAGAGAAACAGGCCCAGAGGGGAGAAACGAC
AGAUAUCCUGACACGGGAGAUUGAAAGGGAGGAAUGAAAUACAAGAUCACUUAC
UGGACCCAUGCAGGGUGCCAGACCCUGGACUAGAUGCUUUAUAACUCUUGUGUA
ACCUUCACAGAAACCCUGCGAAUGUGCACUUCUUGGAGAAAGAGGCUGUGGCUC
AAUGGAAGUUUGCUGAACACAGGGUCACAGGCCAGAGGCGAGUAGCAGAUCAAA
GCCCAGAGCUCCAAAAUGUCACCUAGGCUCCCAGCCAUACAUCACAGCCUUCACC
ACUUCCUGCAAUUCACCCACUGCGUACACCUGU
```

The free energy of the thermodynamic ensemble is **–168.77** kcal/mol.

The frequency of the MFE structure in the ensemble is **0.00** %.

The ensemble diversity is **118.57**.

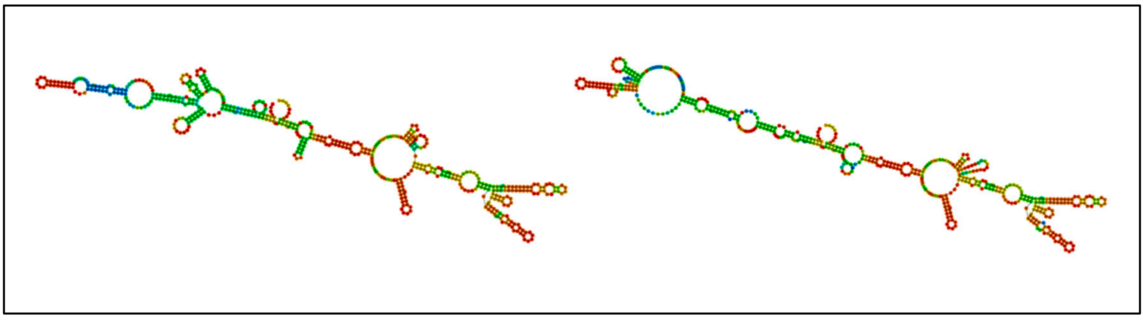

MFE secondary structure and Centroid secondary structure, respectively.

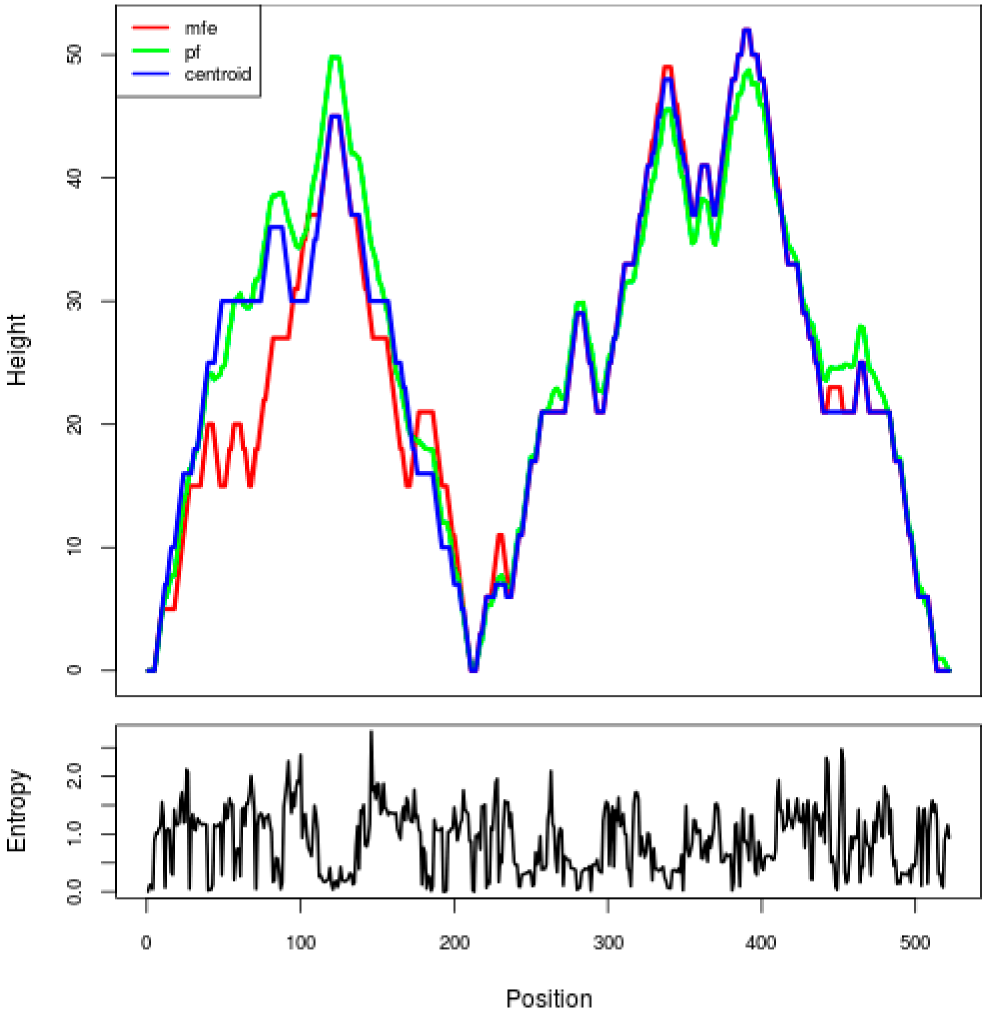

>ENST00000772308.1 lncRNA

```
AGUCUCAGCUGACUUAGCAGGGAGCUCUGGCACCGACUGCCCGUUAGAGAAGGC
CUGUGGUGGGCAGGCAUGUCCAGGCACUGGGACUCUAGCUGUGCAAGACUGUCC
UGGUGCCAGGACUCUUGCUGUGCACAGCCAUGGGCUGGGGCUGCCGGGAACAGC
GUAGAUCCCGAAGGUGCUGCAGCUAGAGCCUGUCGGUUAACUGCACUUCUUGCA
GCCAAAAAUGUGUUUACUCUUGAAGGGGGAGCCGAGGCUGCUACAAGACAGGGU
CUUGCUCUAUCACCCAGGCUGGAGUGCAGUGGUGCAAUCAUGGCUCACUGCAGC
CUCAACCUCCUGGGCUCAAGUGAUCUUCCCAUCUCAGCCUCCCAACUAGCUGGGA
CUACAGGAGCAAUUAAGCCAAUGAGAAAUCAGACCCACACCCCAAUUCUGAUGU
AACAGCCUUGGGAAAGAGGUUGCAGUGAAAAGCUGGUCCUGCUGUGGUGGAGAG
AAUGGAGGAAAGAUAAUAAAAGGCCAAACCUUUGCUCCAA
```

The free energy of the thermodynamic ensemble is **–211.34** kcal/mol.

The frequency of the MFE structure in the ensemble is **0.00** %.

The ensemble diversity is **153.16**.

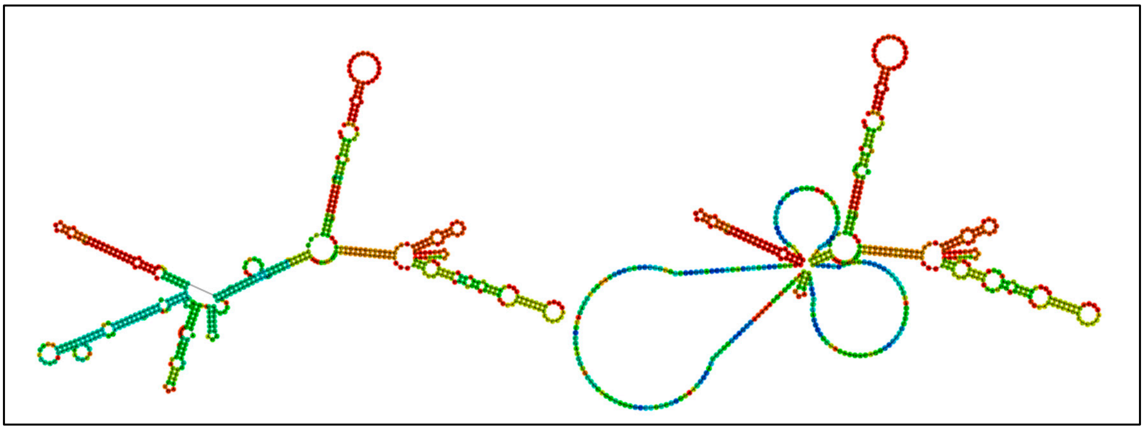

MFE secondary structure and Centroid secondary structure, respectively.

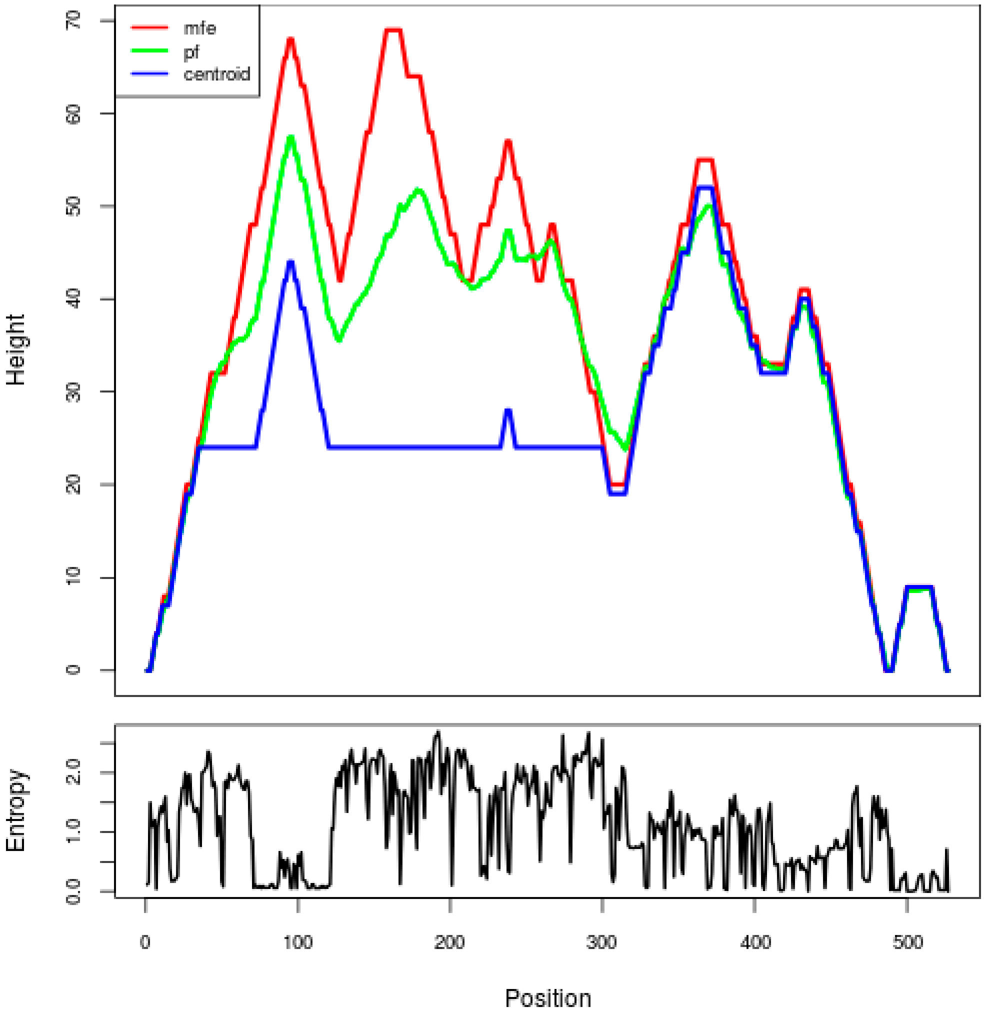

>ENST00000772290.1 lncRNA

```
AGACUUUCUCUCUCUUCUUCCUGGAUCUUUAGGAGCAAUUAAGCCAAUGAGAAA
UCAGACCCACACCCCAAUUCUGAUGUAACAGCCUUGGGAAAGAGGUUGCAGUGA
AAAGCUGGUCCUGCUGUGGUGGAGAGAAUGGAGGAAAGAUAAUAAAAGGCCAAA
CCUUUGCUCCAACUUUCUCCUUAGCUUCCCUUUGGAUCUGGAAAGCUGGGGACCC
ACACGGCAGAGCCAUGGUACUGGAGGAGCCAUUAACAACGAGGCUCCUUCUGUA
AGGUUGCCUGGAUACUGCAUCUGGCUGACAGAGUCCCCAGACAGAACUUGAAUA
GGCACUGCCAGGGUGUGUACUUCUAUGACUCUCCUUUUGCAAUGGGCCCACACC
ACCCACCUCAUUUUUCUAAUAAGUCUUUAAUAAAAGCUUGACUGAAGGUACCAA
GGUGUGCUGAAGUGGAAGCAAAGUUCUCCAAAGUCCAGCAUGGUAGACAUCAGU
GGUGGUAACCAAGGACAGACCCCAAGGCAAGAUGGAGUUUCAAUCUUGUUGC
```

The free energy of the thermodynamic ensemble is **–173.21** kcal/mol.

The frequency of the MFE structure in the ensemble is **0.00** %.

The ensemble diversity is **126.21**.

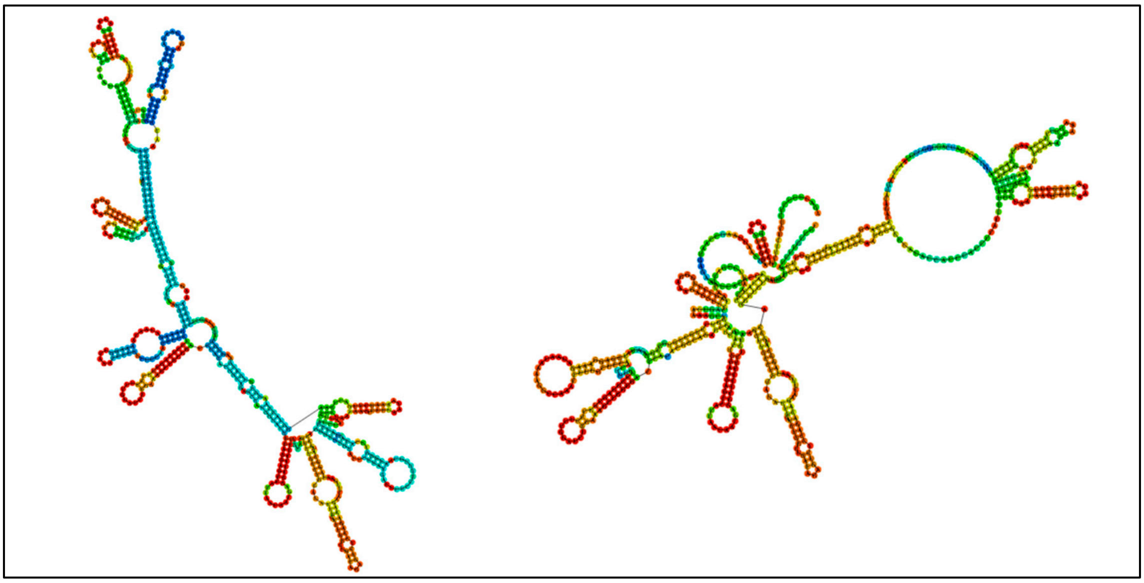

MFE secondary structure and Centroid secondary structure, respectively.

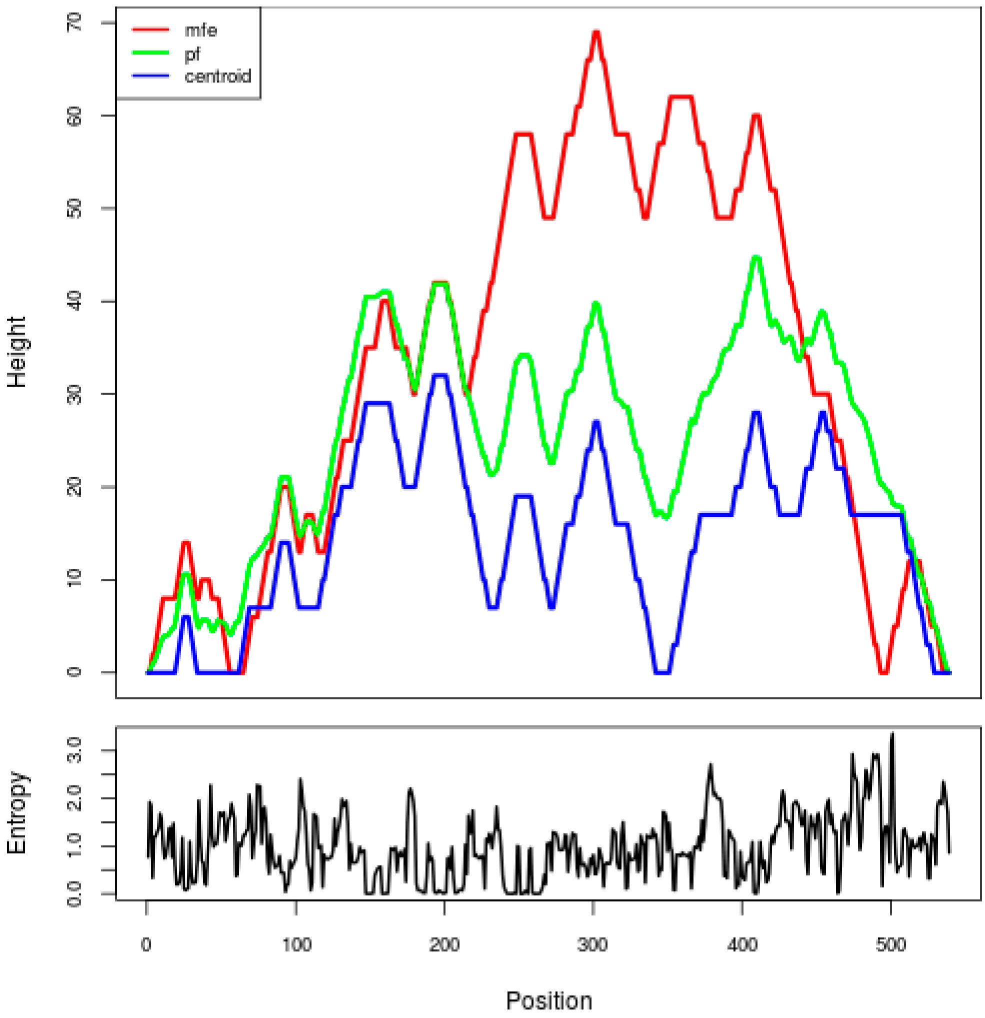

>ENST00000772306.1 lncRNA

```
AGAAGGGCCUCAGACCUUGAUGCGUCUCACAGUCUCAGCUGACUUAGCAGGGAG
CUCUGGCACCGACUGCCCGUUAGAGAAGGCCUGUGGUGGGCAGGCAUGUCCAGGC
ACUGGGACUCUAGCUGUGCAAGACUGUCCUGGUGCCAGGACUCUUGCUGUGCAC
AGCCAUGGGCUGGGGCUGCCGGGAACAGCGUAGAUCCCGAAGGUGCUGCAGCUA
GAGCCUGUCGGUUAACUGCACUUCUUGCAGCCAAAAAUGUGUUUACUCUUGAAG
GGGGAGCCGAGGCUGCUACAGAGCAAUUAAGCCAAUGAGAAAUCAGACCCACAC
CCCAAUUCUGAUGUAACAGCCUUGGGAAAGAGGUUGCAGUGAAAAGCUGGUCCU
GCUGUGGUGGAGAGAAUGGAGGAAAGAUAAUAAAAGGCCAAACCUUUGCUCCAA
CUUUCUCCUUAGCUUCCCUUUGGAUCUGGAAAGCUGGGGACCCACACGGCAGAG
CCAUGGUACUGGAGGAGCCAUUAACAAGUAAGUUCCAAAUAAAUCACCUCCCUC
CCGAGGCAAUGUGUGUUGAGA
```

The free energy of the thermodynamic ensemble is **–218.22** kcal/mol.

The frequency of the MFE structure in the ensemble is **0.00** %.

The ensemble diversity is **98.93**.

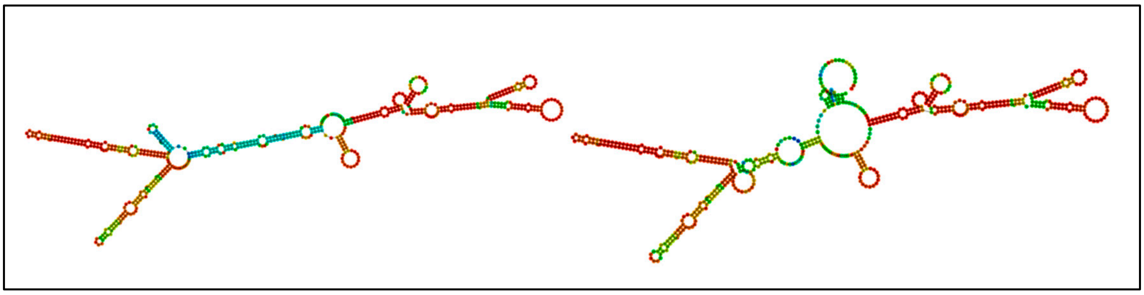

MFE secondary structure and Centroid secondary structure, respectively.

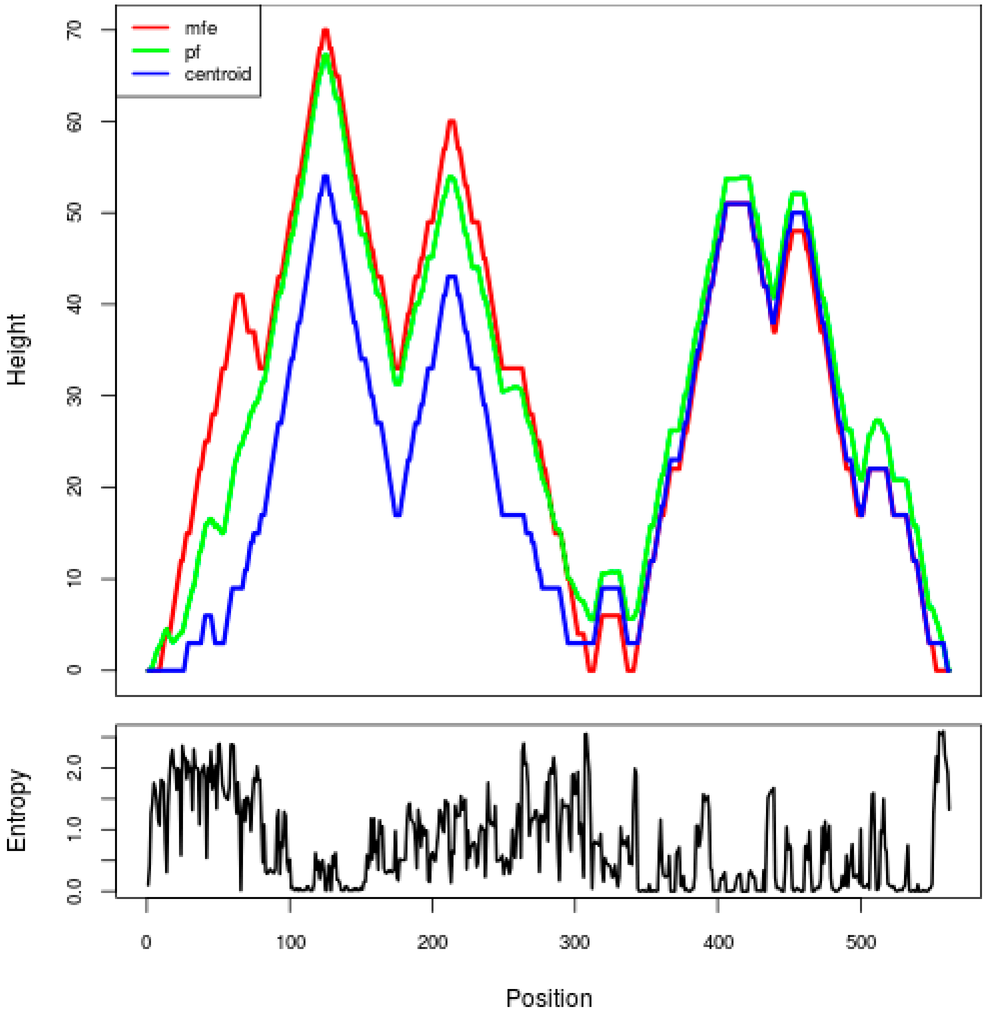

>ENST00000772302.1 lncRNA

```
AGGUCCCAACUCCCUCAGCCGGCUCUGGCCUGACCAAGAGCUCCAGACGGAGCAG
GACUGGAAAUACCUGUCUGCAUUGGCGAGCUCCCAAAUCUUGCCCAGGCGCCUCC
CACCGUGUGCAGCUGAGGAAGGCUGUGCUCUGAGCGUGGGGUUGCCAAGUCAGC
UCCCAAGGCAGCUCUGGCACCAACGACCCAGAGAAACAGGCCCAGAGGGGAGAAA
CGACAGAUAUCCUGACACGGGAGAUUGAAAGGGAGGAAUGAAAUACAAGAUCAC
UUACUGGACCCAUGCAGGGUGCCAGACCCUGGACUAGAUGCUUUAUAACUCUUG
UGUAACCUUCACAGAAACCCUGCGAAGGGGAGGCUGAUGGACUCCUGAAGGACC
GCAACCACUUCCUCGGAUCCACACCAUCUCCAGAGUCUCCUUUGGCUGCCUAUGC
AGGAAGAAAAGAGAAGCAAACAACCCAGCUGCUGUCUCUGGGCUGCUGAACCUG
GCAGUGGAGCUGCCAGAGUAGCGUAAUCUCAGCUGGGCAGCUUCUAUGACUCUC
ACACUAAAUCCUGUUCUCCA
```

The free energy of the thermodynamic ensemble is **–204.82** kcal/mol.

The frequency of the MFE structure in the ensemble is **0.00** %.

The ensemble diversity is **141.91**.

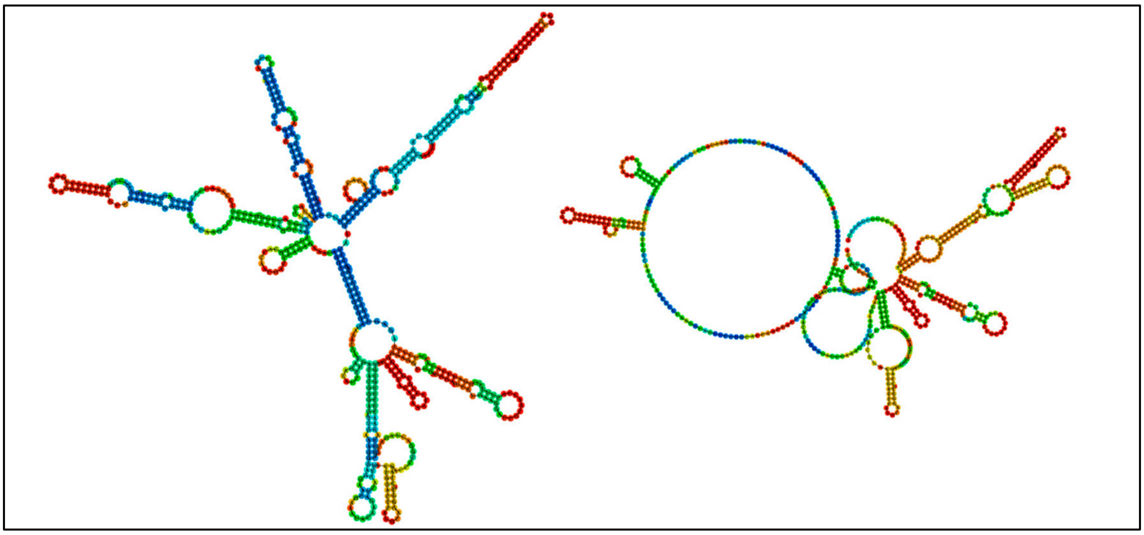

MFE secondary structure and Centroid secondary structure, respectively.

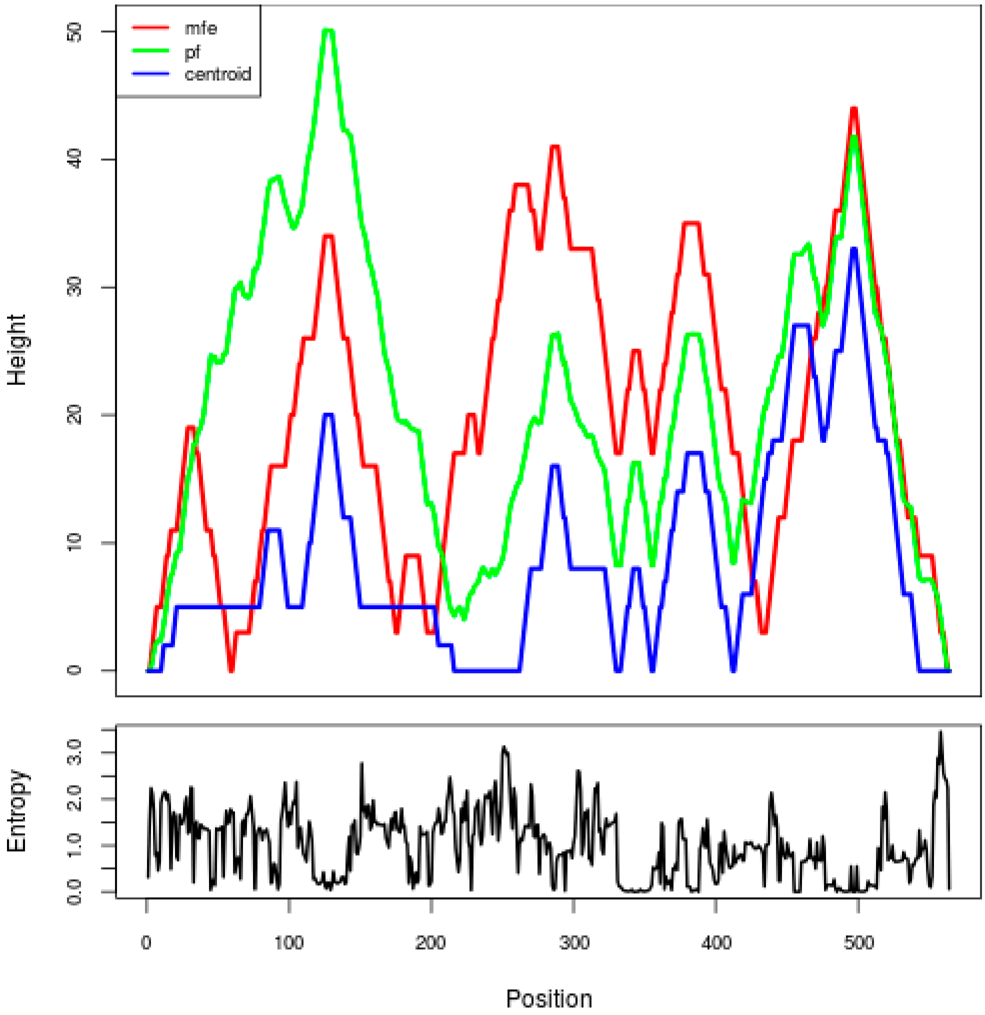

>ENST00000772281.1 lncRNA

```
GAAAAGCUGGUCCUGCUGUGGUGGAGAGAAUGGAGGAAAGAUAAUAAAAGGCCA
AACCUUUGCUCCAACUUUCUCCUUAGCUUCCCUUUGGAUCUGGAAAGCUGGGGA
CCCACACGGCAGAGCCAUGGUACUGGAGGAGCCAUUAACAACCCCUGCCAUUCAC
ACUGUGCAAAGGAGCCUGUCAAUCACUGACUGAUUUUAAGCAGGAAAGUUUCAA
AGAAAGCCCCCAGUACUUACCAAUGUUAAAGAAAAACAGGCCAGUGCUGAAUAA
AUGCUUGUGCUAUUGUGACGCUUCUGACCCAGGCAGGAGACAUGUGCUUCCAUC
UCCAGAACAGCAACCCCUGAAUGACCCCUCGAUACAAUGGAGAAAAGAAGUGCU
GUGUAAUUUAGGGGCAUAAACCAAACCUCCACUGCUUAGAGUCAGCUUAAUGUC
UGACAGAAAUAUACAAAACUGUAAAGCCUCAACCAGACAUCUGAAUAAUCUGUC
CAUACAUUUUUCACAUAUCAGUAUUUAUUAUCUUUUCAAAGGAACAGGUCAAGA
UGAGAAUAAAGUUUAUGAUCAAAGGUA
```

The free energy of the thermodynamic ensemble is **–157.69** kcal/mol.

The frequency of the MFE structure in the ensemble is **0.00** %.

The ensemble diversity is **93.17**.

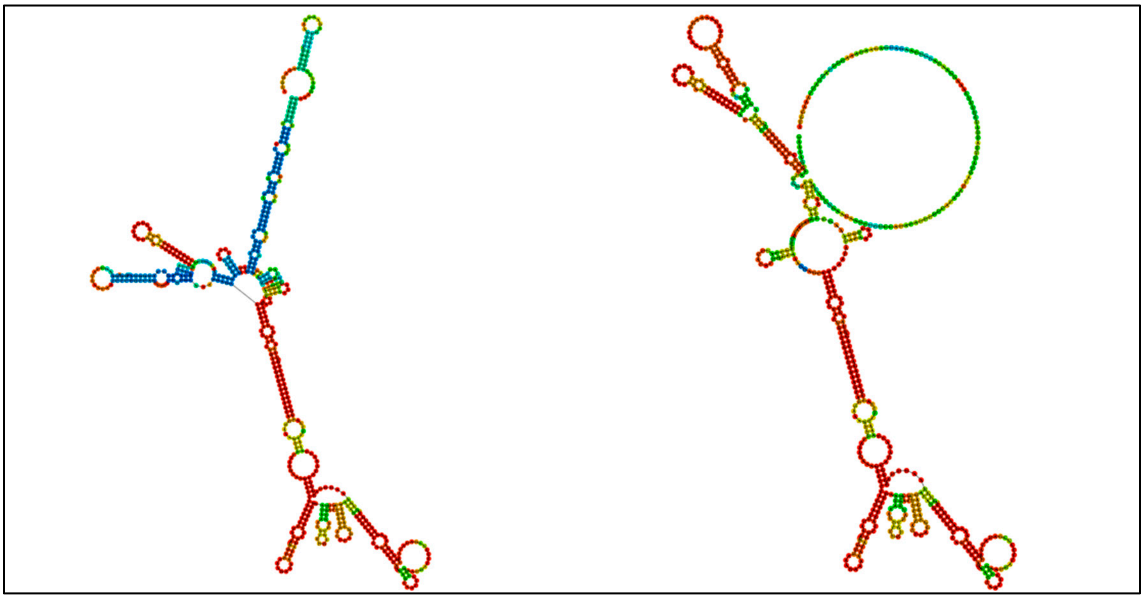

MFE secondary structure and Centroid secondary structure, respectively.

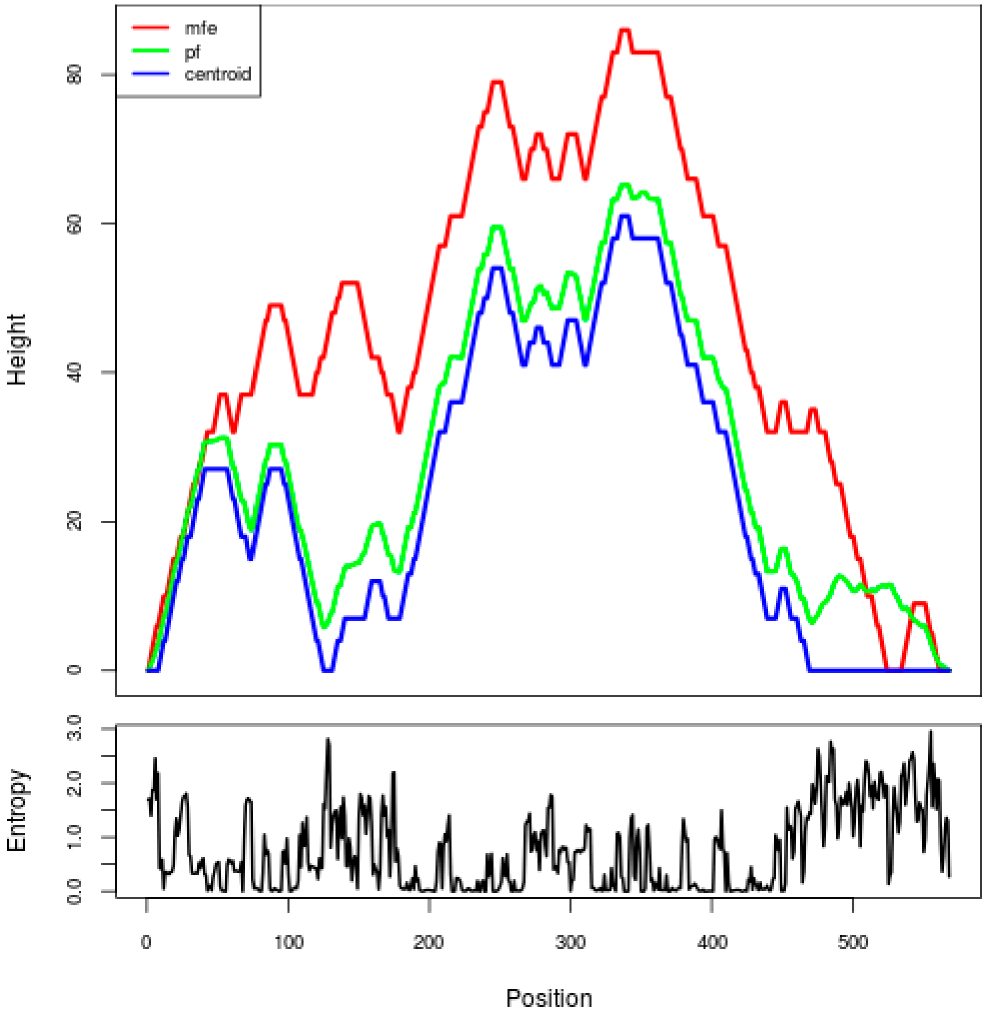

>ENST00000772282.1 lncRNA

```
GUGGUGGAGAGAAUGGAGGAAAGAUAAUAAAAGGCCAAACCUUUGCUCCAACUU
UCUCCUUAGCUUCCCUUUGGAUCUGGAAAGCUGGGGACCCACACGGCAGAGCCA
UGGUACUGGAGGAGCCAUUAACAACCAUCCAGACUGAGGAAAUGUAGUCACCAC
AGGACCCAGAAAUUUUGGAGCCAGCCCCUGCCAUUCACACUGUGCAAAGGAGCC
UGUCAAUCACUGACUGAUUUUAAGCAGGAAAGUUUCAAAGAAAGCCCCCAGUAC
UUACCAAUGUUAAAGAAAAACAGGCCAGUGCUGAAUAAAUGCUUGUGCUAUUGU
GACGCUUCUGACCCAGGCAGGAGACAUGUGCUUCCAUCUCCAGAACAGCAACCCC
UGAAUGACCCCUCGAUACAAUGGAGAAAAGAAGUGCUGUGUAAUUUAGGGGCAU
AAACCAAACCUCCACUGCUUAGAGUCAGCUUAAUGUCUGACAGAAAUAUACAAA
ACUGUAAAGCCUCAACCAGACAUCUGAAUAAUCUGUCCAUACAUUUUUCACAUA
UCAGUAUUUAUUAUCUUUUCAAAGGAACAGGUCAAGAUGAGAAUAAAGUUUAU
GAUCAAA
```

The free energy of the thermodynamic ensemble is **–164.69** kcal/mol.

The frequency of the MFE structure in the ensemble is **0.00** %.

The ensemble diversity is **89.09**.

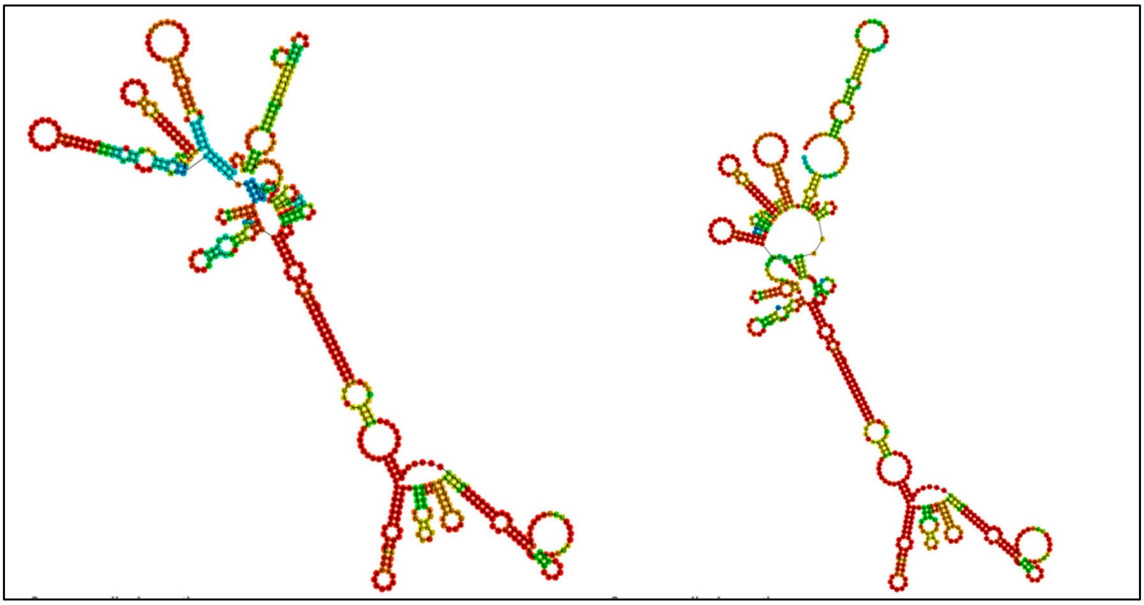

MFE secondary structure and Centroid secondary structure, respectively.

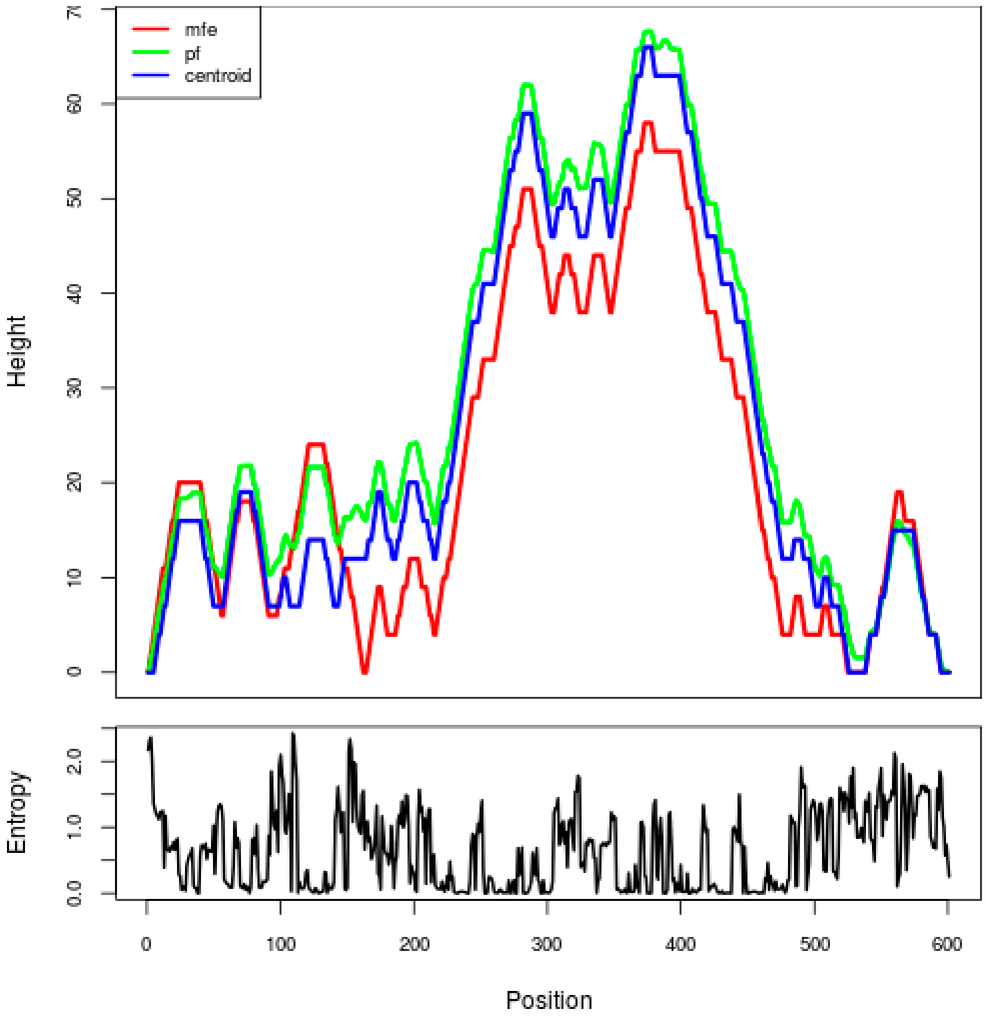

>ENST00000772296.1 lncRNA

```
UAGACUUUCUCUCUCUUCUUCCUGGAUCUUUAGGAGCAAUUAAGCCAAUGAGAA
AUCAGACCCACACCCCAAUUCUGAUGUAACAGCCUUGGGAAAGAGGUUGCAGUG
AAAAGCUGGUCCUGCUGUGGUGGAGAGAAUGGAGGAAAGAUAAUAAAAGGCCAA
ACCUUUGCUCCAACUUUCUCCUUAGCUUCCCUUUGGAUCUGGAAAGCUGGGGAC
CCACACGGCAGAGCCAUGGUACUGGAGGAGCCAUUAACAAUCUUUAAUAAAAGC
UUGACUGAAGGUACCAAGGUGUGCUGAAGUGGAAGCAAAGUUCUCCAAAGUCCA
GCAUGGUAGACAUCAGUGGUGGUAACCAAGGACAGACCCCAAGGCAAGGUGAAC
CUCAAAAAUGGAACCUCAAGUCUAUGCAGUCCAGCUGCCCUCCCCACCAGAAAGU
CCUUGUUCCAGCCCAACAUCAGUGCCUCUGAGUUUGUUUACUAGAAACAAAGGA
AGAAUUUCCUUGUAAAAAUAUAGACAGAGUAGUCCCUGGCUUUCUCCUCUUGCA
GGAAGGAUGGAUUCUCCCAUUCCAUACCAUCUUUCCCCCACACUGGCCCCAGAAA
UACUUAAUUCAACUAUGUGAAAAUAAAGAUUGUUUUUGGUUUGAGGGCAUAGG
GAUCCAUUUAUCCUUAUUCUUUAUGAGGCACUAAAUUAGCUUUGUAUGUUAUUA
AAUGUGUCUCGUCAAUGCUG
```

The free energy of the thermodynamic ensemble is **–215.05** kcal/mol.

The frequency of the MFE structure in the ensemble is **0.00** %.

The ensemble diversity is **168.46**.

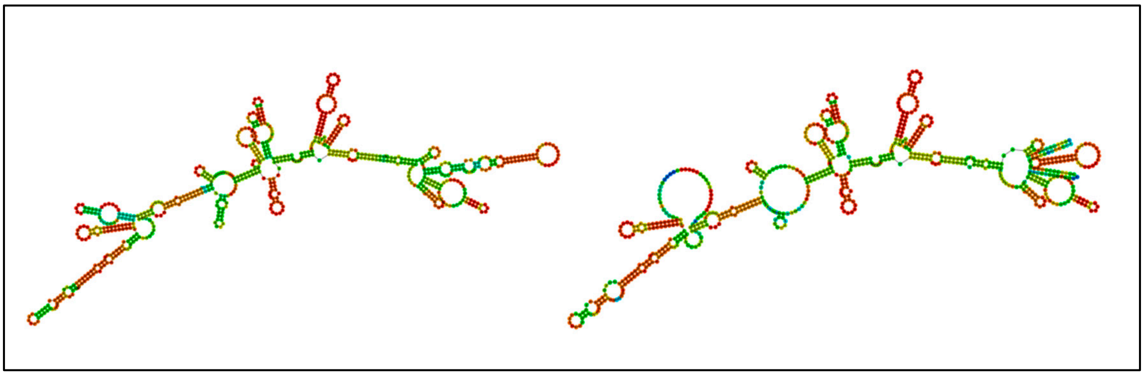

MFE secondary structure and Centroid secondary structure, respectively.

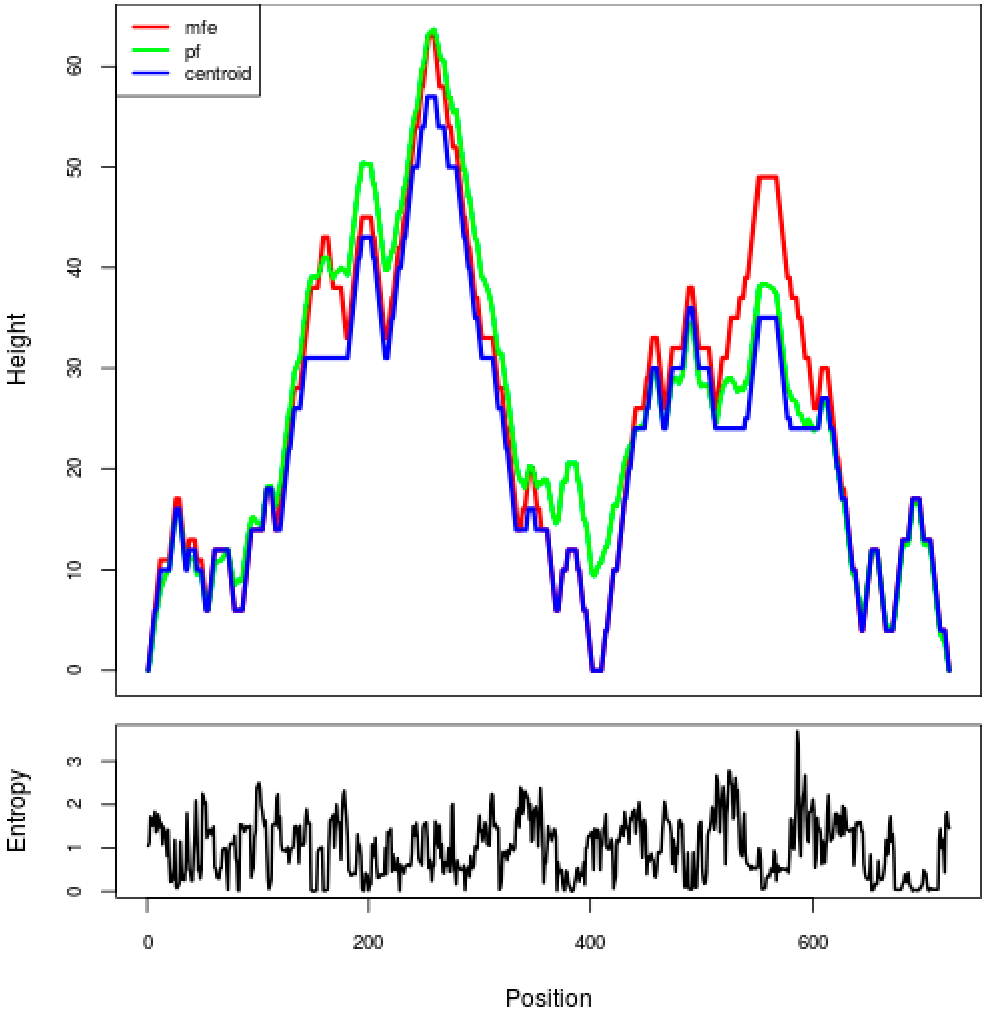

>ENST00000772297.1 lncRNA

```
GGUUGCAGUGAAAAGCUGGUCCUGCUGUGGUGGAGAGAAUGGAGGAAAGAUAAU
AAAAGGCCAAACCUUUGCUCCAACUUUCUCCUUAGCUUCCCUUUGGAUCUGGAA
AGCUGGGGACCCACACGGCAGAGCCAUGGUACUGGAGGAGCCAUUAACAAAGCU
UUCAAUAAACCUCUCUUUCUUGAAGUUACCUGAGAAUGGAUCCAUUCCCUGCAA
CUGAAGAUUCUAAGGAACUGGGUUUCUCAGUAUACAAUGGGAAUGGUUGGGAGG
AGUCUUUAAUAAAAGCUUGACUGAAGGUACCAAGGUGUGCUGAAGUGGAAGCAA
AGUUCUCCAAAGUCCAGCAUGGUAGACAUCAGUGGUGGUAACCAAGGACAGACC
CCAAGGCAAGGUGAACCUCAAAAAUGGAACCUCAAGUCUAUGCAGUCCAGCUGC
CCUCCCCACCAGAAAGUCCUUGUUCCAGCCCAACAUCAGUGCCUCUGAGUUUGUU
UACUAGAAACAAAGGAAGAAUUUCCUUGUAAAAAUAUAGACAGAGUAGUCCCUG
GCUUUCUCCUCUUGCAGGAAGGAUGGAUUCUCCCAUUCCAUACCAUCUUUCCCCC
ACACUGGCCCCAGAAAUACUUAAUUCAACUAUGUGAAAAUAAAGAUUGUUUUG
GUUUGAGGGCAUAGGGAUCCAUUUAUCCUUAUUCUUUAUGAGGCACUAAAUUAG
CUUUGUAUGUUAUUAAAUGUGUCUCGUCAA
```

The free energy of the thermodynamic ensemble is **–226.07** kcal/mol.

The frequency of the MFE structure in the ensemble is **0.00** %.

The ensemble diversity is **163.11**.

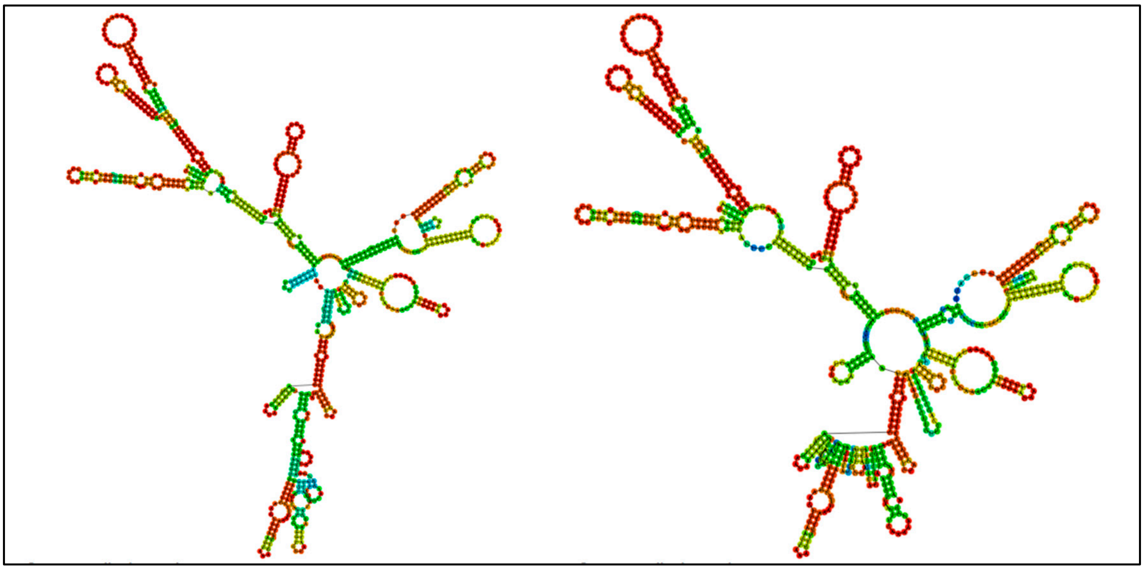

MFE secondary structure and Centroid secondary structure, respectively.

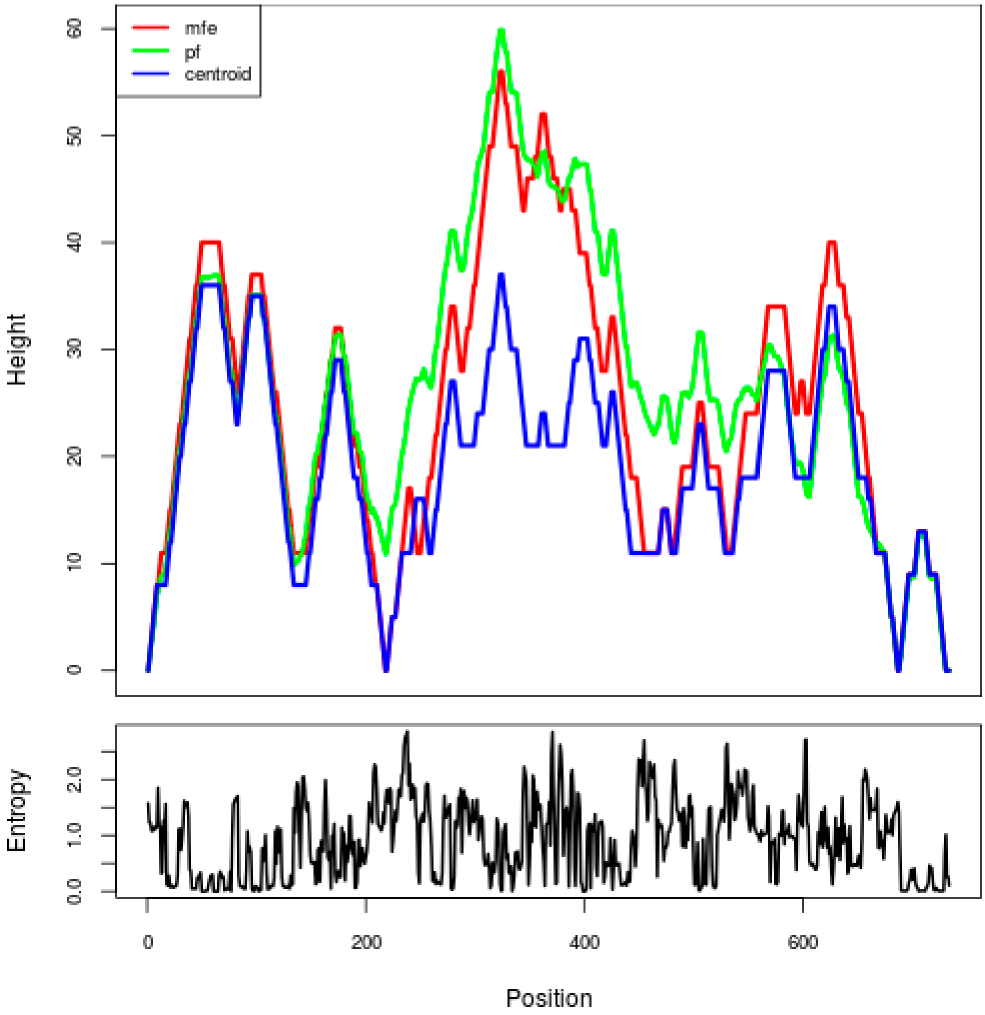

>ENST00000772301.1 lncRNA

```
GGGAGUUGUAGUUUCAGGAGGGAAGAGCUCGGCUGCACGUGGGCUCAGGUCGCC
CCCUCGUGGACCUCUGCACAGCCAAGCGUGGAAAGGCCGGUGGGCCGCUUCCCGC
CGCCAGCAAGAGGGGCAGCAGGGUCCGGCUCGCGCUCCAGGGCUGCGGGAGCUGG
CCCCUUGCCAGGCUACUGCAGGACUGGAAAUACCUGUCUGCAUUGGCGAGCUCCC
AAAUCUUGCCCAGGCGCCUCCCACCGUGUGCAGCUGAGGAAGGCUGUGCUCUGAG
CGUGGGGUUGCCAAGUCAGCUCCCAAGGCAGCUCUGGCACCAACGACCCAGAGAA
ACAGGCCCAGAGGGGAGAAACGACAGAUAUCCUGACACGGGAGAUUGAAAGGGA
GGAAUGAAAUACAAGAUCACUUACUGGACCCAUGCAGGGUGCCAGACCCUGGAC
UAGAUGCUUUAUAACUCUUGUGUAACCUUCACAGAAACCCUGCGAAGAGCAAUU
AAGCCAAUGAGAAAUCAGACCCACACCCCAAUUCUGAUGUAACAGCCUUGGGAA
AGAGGUUGCAGUGAAAAGCUGGUCCUGCUGUGGUGGAGAGAAUGGAGGAAAGAU
AAUAAAAGGCCAAACCUUUGCUCCAACUUUCUCCUUAGCUUCCCUUUGGAUCUG
GAAAGCUGGGGACCCACACGGCAGAGCCAUGGUACUGGAGGAGCCAUUAACAAG
UAAGUUCCAAAUAAAUCACCUCCCUCCCGA
```

The free energy of the thermodynamic ensemble is **–294.03** kcal/mol.

The frequency of the MFE structure in the ensemble is **0.00** %.

The ensemble diversity is **168.25**.

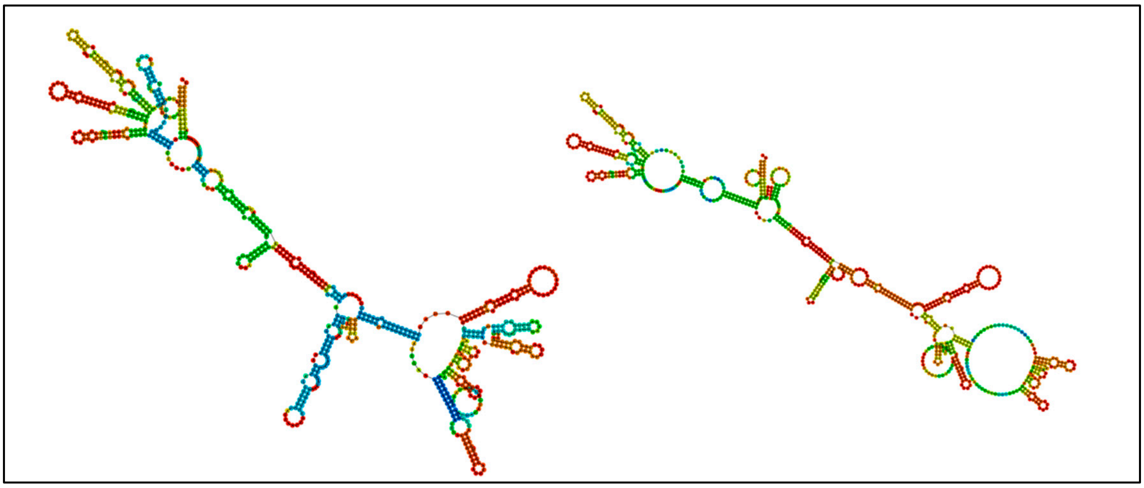

MFE secondary structure and Centroid secondary structure, respectively.

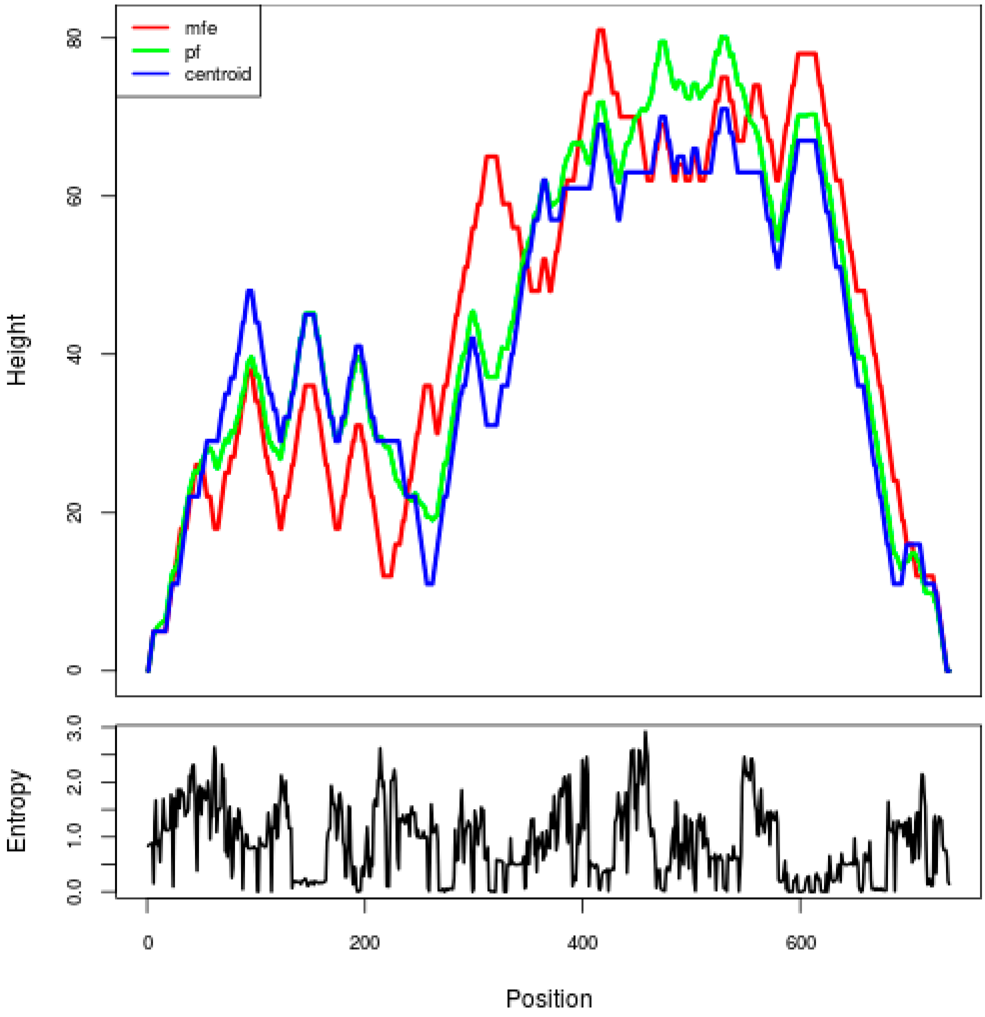

>ENST00000772294.1 lncRNA

AUGGAGGAAAGAUAAUAAAAGGCCAAACCUUUGCUCCAACUUUCUCCUUAGCUU<br>CCCUUUGGAUCUGGAAAGCUGGGGACCCACACGGCAGAGCCAUGGUACUGGAGG<br>AGCCAUUAACAAAGCUUUCAAUAAACCUCUCUUUCUUGAAGUUACCUGAGAAUG<br>GAUCCAUUCCCUGCAACUGAAGAUUCUAAGGAACUGGGUUUCUCAGUAUACAAU<br>GGGAAUGGUUGGGAGGAGGUAAAGAGUAGAAGACAGUAUCAAGAAUCCAGAGCC<br>CAGCACCUGUAGUCCUAACUAUUCAGAUUCCUUGAGCCCAGGAGUUUGAGUCCA<br>GCCUGGACAACAUAUUGAGACCCCCAUCUCUCUAAAAAAAAAGAGAAAGAAAGA<br>AGGAAAGAAAAAAAGAAAGAAAGAAAGAAAGAAAGAAAGAAAGAAAGAAAGAA<br>AGAAAGAGAAAGAAAGAAGGAAAGAAGGAAAGAAGGAAAGAAAGAAAGAAAGA<br>GAAAGAAAGAAAAGAAGAUUGUAGCUAGGGGGAGAGUAGGUGAAAAGAUGAAC<br>AACAUGACCGGGAAGAUUUCCUAAUCUCACCACAGCCUGGCUCUACCUUAAGUC<br>UUUAAUAAAAGCUUGACUGAAGGUACCAAGGUGUGCUGAAGUGGAAGCAAAGUU<br>CUCCAAAGUCCAGCAUGGUAGACAUCAGUGGUGGUAACCAAGGACAGACCCCAA<br>GGCAAGAUGGAGUUUCAAUCUUGUUGCCCAGGCUGGAGUGCA

The free energy of the thermodynamic ensemble is **–196.33** kcal/mol.

The frequency of the MFE structure in the ensemble is **0.00** %.

The ensemble diversity is **157.69**.

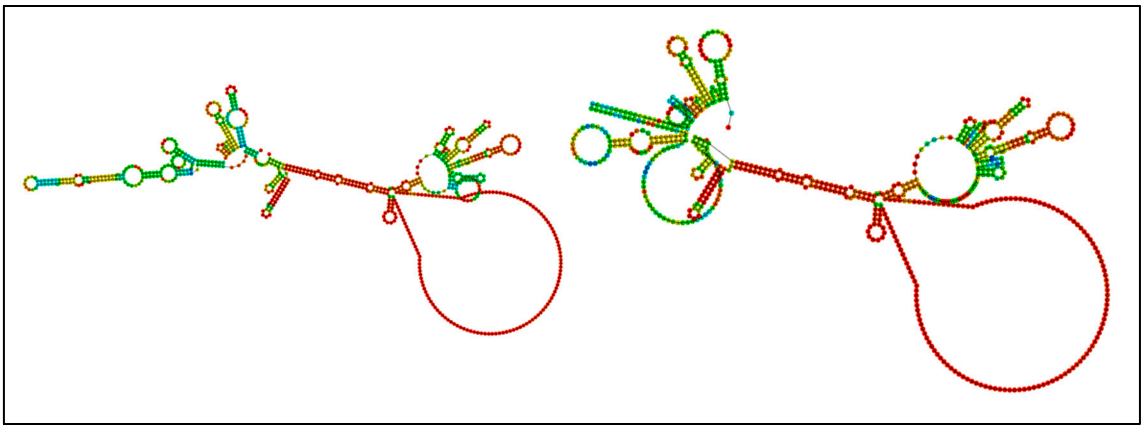

MFE secondary structure and Centroid secondary structure, respectively.

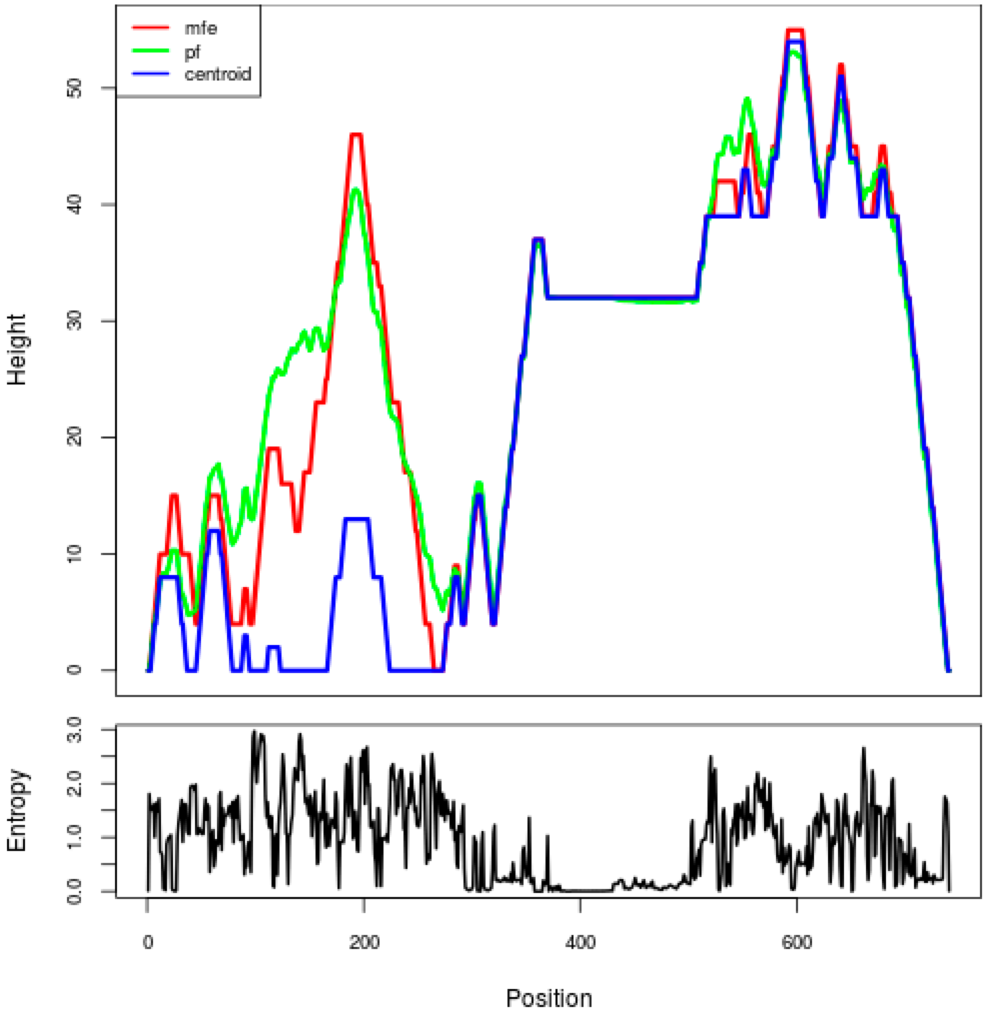

>ENST00000701616.2 lncRNA

```
UGAUGUAACAGCCUUGGGAAAGAGGUUGCAGUGAAAAGCUGGUCCUGCUGUGGU
GGAGAGAAUGGAGGAAAGAUAAUAAAAGGCCAAACCUUUGCUCCAACUUUCUCC
UUAGCUUCCCUUUGGAUCUGGAAAGCUGGGGACCCACACGGCAGAGCCAUGGUA
CUGGAGGAGCCAUUAACAACGAGGCUCCUUCUGUAAGGUUGCCUGGAUACUGCA
UCUGGCUGACAGAGUCCCCAGACAGAACUUGAAUAGGCACUGCCAGGGUGUGUA
CUUCUAUGACUCUCCUUUUGCAAUGGGCCCACACCACCCACCUCAUUUUUCUAAU
AAGUCUUUAAUAAAAGCUUGACUGAAGGUACCAAGGUGUGCUGAAGUGGAAGCA
AAGUUCUCCAAAGUCCAGCAUGGUAGACAUCAGUGGUGGUAACCAAGGACAGAC
CCCAAGGCAAGGUGAACCUCAAAAAUGGAACCUCAAGUCUAUGCAGUCCAGCUG
CCCUCCCCACCAGAAAGUCCUUGUUCCAGCCCAACAUCAGUGCCUCUGAGUUUGU
UUACUAGAAACAAAGGAAGAAUUUCCUUGUAAAAAUAUAGACAGAGUAGUCCCU
GGCUUUCUCCUCUUGCAGGAAGGAUGGAUUCUCCCAUUCCAUACCAUCUUUCCCC
CACACUGGCCCCAGAAAUACUUAAUUCAACUAUGUGAAAAUAAAGAUUGUUUUU
GGUUUGAGGGCAUAGGGAUCCAUUUAUCCUUAUUCUUUAUGAGGCACUAAAUUA
GCUUUGUAUGUUAUUAAAUGUGUCUCGUCAAUGCUG
```

The free energy of the thermodynamic ensemble is **–249.02** kcal/mol.

The frequency of the MFE structure in the ensemble is **0.00** %.

The ensemble diversity is **166.54**.

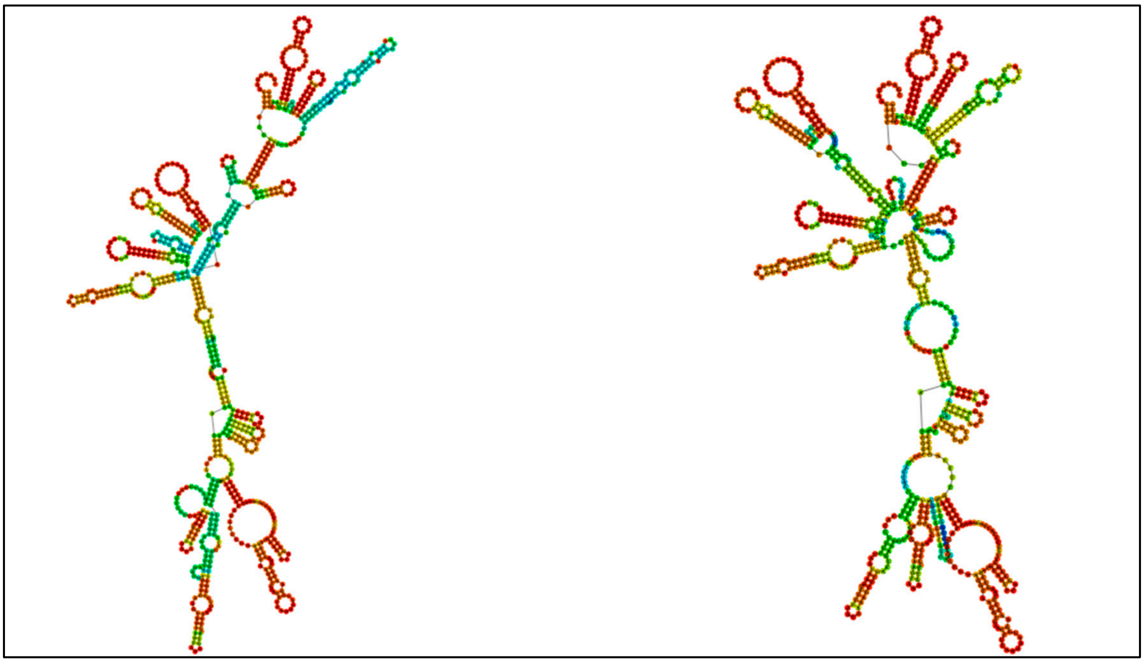

MFE secondary structure and Centroid secondary structure, respectively.

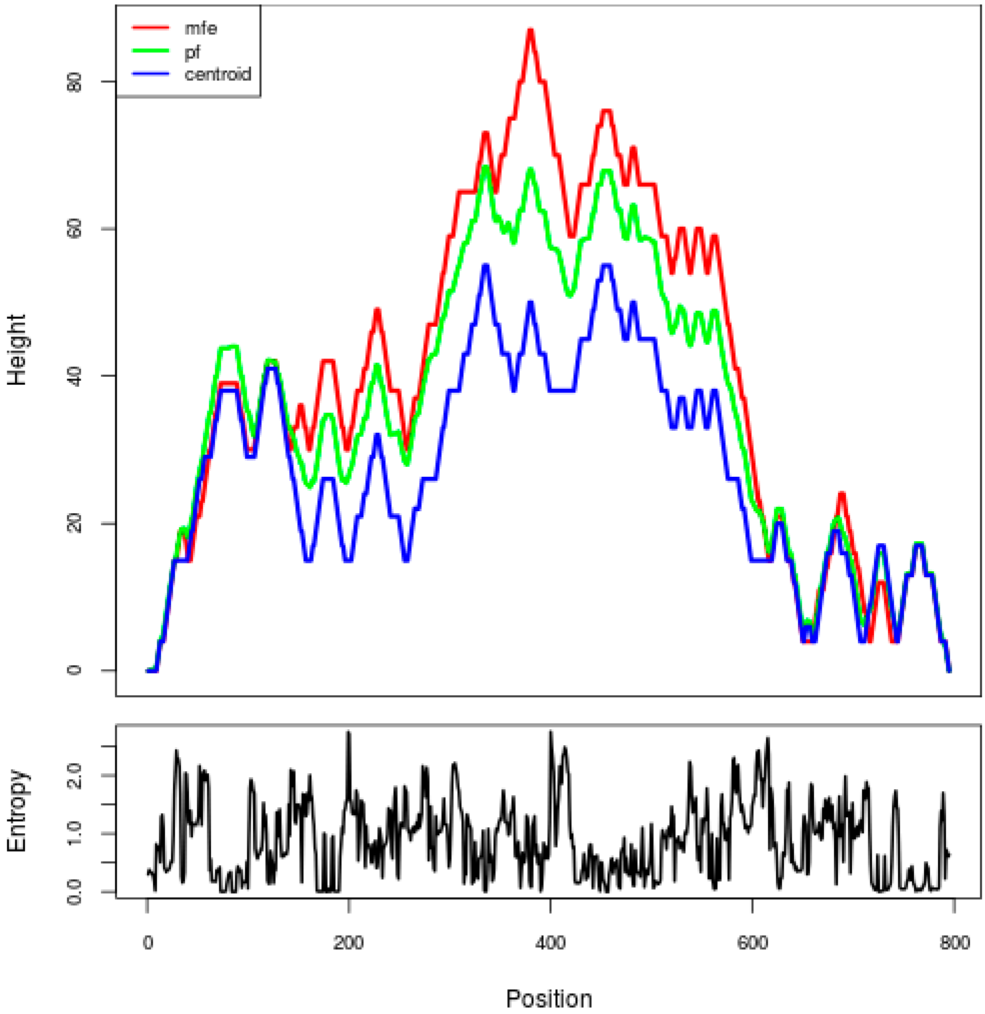

>ENST00000772283.1 lncRNA

```
GACCCACACCCCAAUUCUGAUGUAACAGCCUUGGGAAAGAGGUUGCAGUGAAAA
GCUGGUCCUGCUGUGGUGGAGAGAAUGGAGGAAAGAUAAUAAAAGGCCAAACCU
UUGCUCCAACUUUCUCCUUAGCUUCCCUUUGGAUCUGGAAAGCUGGGGACCCAC
ACGGCAGAGCCAUGGUACUGGAGGAGCCAUUAACAAGUCUCUGUGACAGAGAUG
AGGUGGGGCAUACACUAUAUUCCUUCUUUCCUGGCCUUGAAUGUGGAGUCAUUU
CAUUUAUGUGAAGAAAUGCUGACGUUGCAGACAGCCCCAUCUUCCCAGUGUUCC
CCCAAUCUUUCUGGGGAACUUCCAUUUGCUAGGGAGAGCUGCAUGCCUUCCUAU
CUGGGACACUGGACAUAGAUUUUCCUGGCUCUGGGACUCUCUGGGCCAAGGUCU
GAAUGAGGAUAAGGUGAAGGAGGCAUCACUUUUCAAAAUUUAAGGGAGUGCUAC
AGUUUACUCUCUCUUCUUGGGCUGGGACAUCUAUCUGCUGCUGCCUUUGGAAAU
UGGAGCUCCUGGUCCUCAGGCCUUUAGACUCUAGGACUUGACACCAGCAGUUCCC
CACCCCAUCCCCAACCCCAAGCUCCAGUUCUCAGACUUUUGGUCUUGGACCGUUA
CACCAUCGGCUCUCCUGGUUCUCAGGCCUCUGGACUUGAACUGAAUUACACCACU
GGCUUACCUGGUUCUCCAGCUUGCAAACAGCAUAUCAUGGAAUUUUUUGGCCUC
UAUAGUCACACGAGCUAAUUCUCAUAAUAAAUCUCCUCUCAUGCA
```

The free energy of the thermodynamic ensemble is **–269.07** kcal/mol.

The frequency of the MFE structure in the ensemble is **0.00** %.

The ensemble diversity is **189.43**.

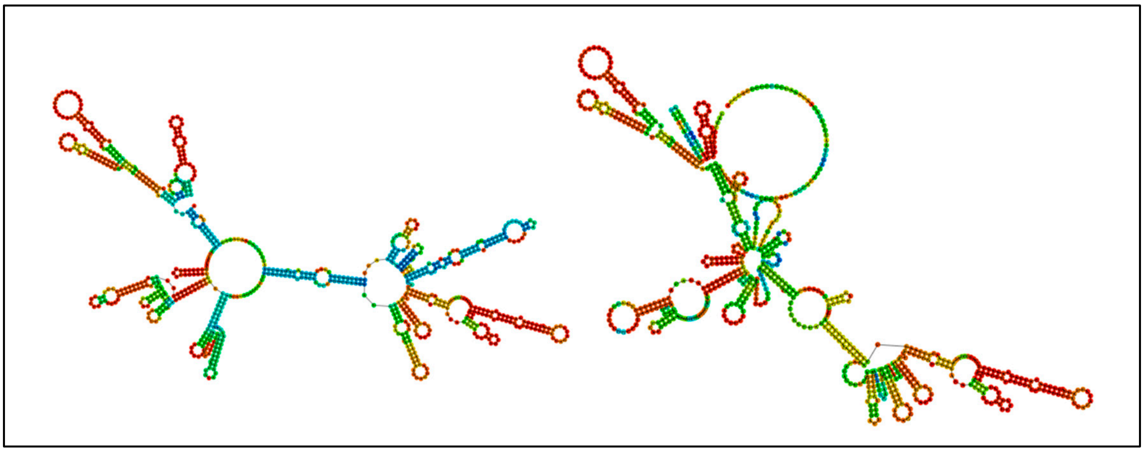

MFE secondary structure and Centroid secondary structure, respectively.

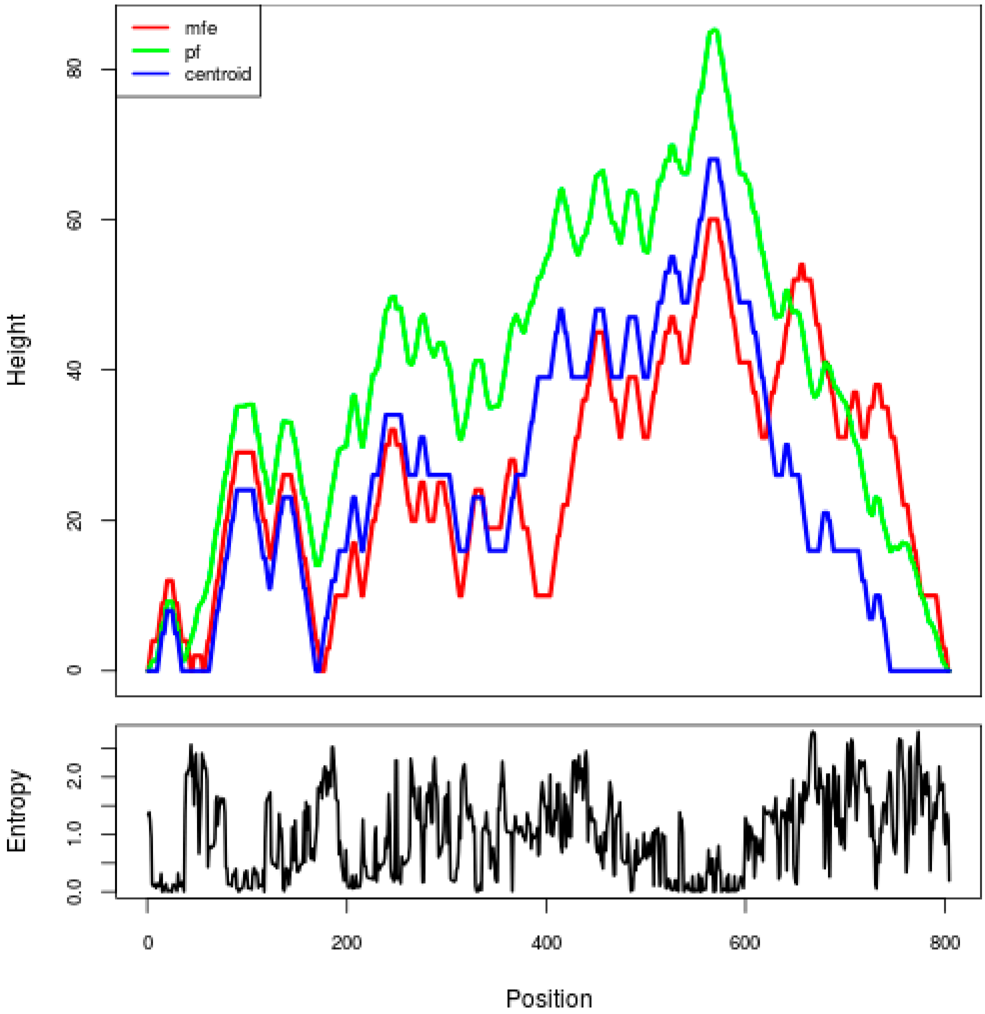

>ENST00000772285.1 lncRNA

```
AGAAGGGCCUCAGACCUUGAUGCGUCUCACAGUCUCAGCUGACUUAGCAGGGAG
CUCUGGCACCGACUGCCCGUUAGAGAAGGCCUGUGGUGGGCAGGCAUGUCCAGGC
ACUGGGACUCUAGCUGUGCAAGACUGUCCUGGUGCCAGGACUCUUGCUGUGCAC
AGCCAUGGGCUGGGGCUGCCGGGAACAGCGUAGAUCCCGAAGGUGCUGCAGCUA
GAGCCUGUCGGUUAACUGCACUUCUUGCAGCCAAAAAUGUGUUUACUCUUGAAG
GGGGAGCCGAGGCUGCUACACUUCCCUUUGGAUCUGGAAAGCUGGGGACCCACA
CGGCAGAGCCAUGGUACUGGAGGAGCCAUUAACAAUCUUUAAUAAAAGCUUGAC
UGAAGGUACCAAGGUGUGCUGAAGUGGAAGCAAAGUUCUCCAAAGUCCAGCAUG
GUAGACAUCAGUGGUGGUAACCAAGGACAGACCCCAAGGCAAGGUGAACCUCAA
AAAUGGAACCUCAAGUCUAUGCAGUCCAGCUGCCCUCCCCACCAGAAAGUCCUUG
UUCCAGCCCAACAUCAGUGCCUCUGAGUUUGUUUACUAGAAACAAAGGAAGAAU
UUCCUUGUAAAAAUAUAGACAGAGUAGUCCCUGGCUUUCUCCUCUUGCAGGAAG
GAUGGAUUCUCCCAUUCCAUACCAUCUUUCCCCCACACUGGCCCCAGAAAUACUU
AAUUCAACUAUGUGAAAAUAAAGAUUGUUUUUGGUUUGAGGGCAUAGGGAUCC
AUUUAUCCUUAUUCUUUAUGAGGCACUAAAUUAGCUUUGUAUGUUAUUAAAUG
UGUCUCGUCAA
```

The free energy of the thermodynamic ensemble is **–284.44** kcal/mol.

The frequency of the MFE structure in the ensemble is **0.00** %.

The ensemble diversity is **206.37**.

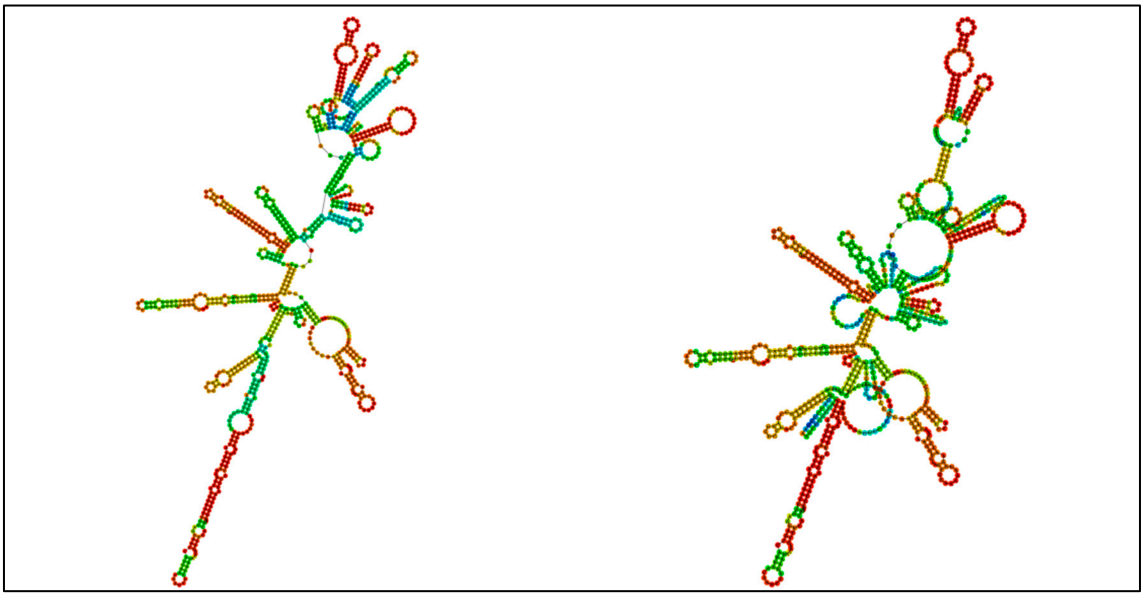

MFE secondary structure and Centroid secondary structure, respectively.

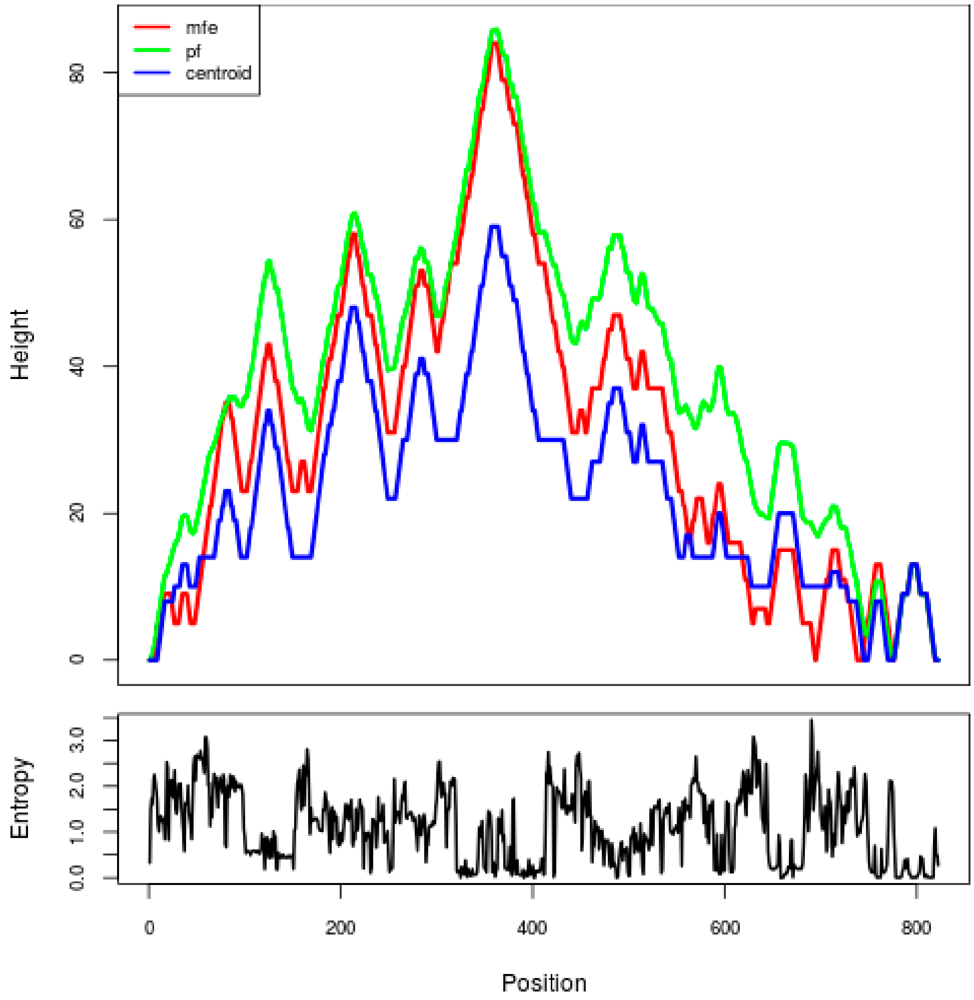

>Consensus structure lncRNA LINC01133

```
UAGUGACGGGGUCUCACACCUUGAUGCGUCUCACACUCUCAGCUGACUUAGCAG
GGUAGCUCUGGCACCGACUGCCCGUAUUAGAGAAGGUGCACCUGUGGUGGGCAG
GCAUGUCCAGGCACUGGGACUCUAGCUGUGCAAGACUGUCCUGGUGCCAGGACU
CUUGCUGUGCACAGCCAUGGGCUGGGGCUGCCGGGAACAGCGUAGAUCCCGAAG
GUGCUGCAGCUAGAGCCUGUCGGUUAACUGCACUUCUUGCAGCCAAAAAUGUGU
UUACUCUUGAAGGGGGAGCCGAGGCUGCUACAGAGCAAUGUCUUGCUCUAUCAC
CCAGGCUGGAGUGCAGUGGUGCAAUCAUGGCUUAAGCCAAUGAGAAAUUCAGAC
CCACACCCCAAUUCUGAUGUAACAGCCUUGGGAAAGAGCUCGGUUGCAAGUGAA
AAGCUGGUCCUGCAUGUGGUGGAGAGAAUGCGGAGGAAAGAAGAUAAUAAAAGG
CCAAACCUUUGCUCCAACUUUCUCCCUUAGCUUCCCUUUGGAUCUGGGAAAGCU
GGGUCCGACCCACACGGCAGAGCCGCAGGAGCUGGCCCCUUGCCAUGGUACUGGA
GGGGUGGACCCGGUGGAAAUACCUGUCUGCAUUCGGCGAGCUCCCAAAUCUUGC
CCAGGCGCCUCCCACCGUGUGCAGCUGAGGAAGGCUGUGCUCUGAGCGUGGGGUU
GCCAAGUCAGCUCCCAAGGCAGCUCUGGCACCAACGACCCAGAGAAACAGGCCCA
GAGGGGAGAAACGACAGAUAUCCUGACACGGGAGAUUGAAAGGGAGGAAUGAAA
UACAAGAUCACUUACUGGACCCAUGCAGGGUGCCAGACCCUGGACUAGAUGCUU
UAUAACUCUUGUGUAGCCAUUAACAGAAACCCUGCGAAGGGGGGAGUUUCAAUC
UUGUUGCCCAGGCUGGAGUGCAAUGGUGCGAUCUCCAUGGACUUCACUGUAACC
UCCGCCUCCCAGGAGAAUCGCUUGAACCCGGGAGGCAGAGGUUGCAACCACUUCC
GUGAGCUGGAUCCAAUACACUGCACCACUGUACUCCAUCUCCAGAGUCUCCUUU
GGCUGACAGAGUGAGACUCCGUCCCAGAAAAACAGAACUUGAAAAAUAGGCACA
UGUCAACACCAGGGUGUGUAGCUUAUAUCCCAAUAUCUUGGCCAUUCAUUAGAA
CCCUGGUUAGGCUCCUCUCUUAUCAACUUACCUCUAUGAACCAGCUUCUGCCUCC
UUUUGCAGGAAUCAGAGGAGGAAUGGGCCCAAUUAGCACCACGUGUCUUCCAUC
CACUGUUCCCCUCAUUUCUUCUAAUAUGGAGAGUCAUCACAGCCUCUUCAAUUA
AACCUCUCUUUCUUGAAGUUUACCUGAGGAAUGAGGGGCAUACACUAGAUCCAU
UCUGUGCCCCUGCAACUGAAGAUUCUAAGGCAGCACAUAUGAUAACUGGGUUCA
UUUCAUUUAUGUUAAGAAAAGGAGCCUCUCAGUAGACCUGGACUGGAGUUACAA
UCAAGGGGCCAGAAUGGCGUAUUGGGAGUCAGAGGGGGAGAGGUAAAGAGCCCC
UUUCCUAGGAAGAGGGUCCUCCAGUAU
```

The free energy of the thermodynamic ensemble is **–594.69** kcal/mol.

The frequency of the MFE structure in the ensemble is **0.00** %.

The ensemble diversity is **416.68**.

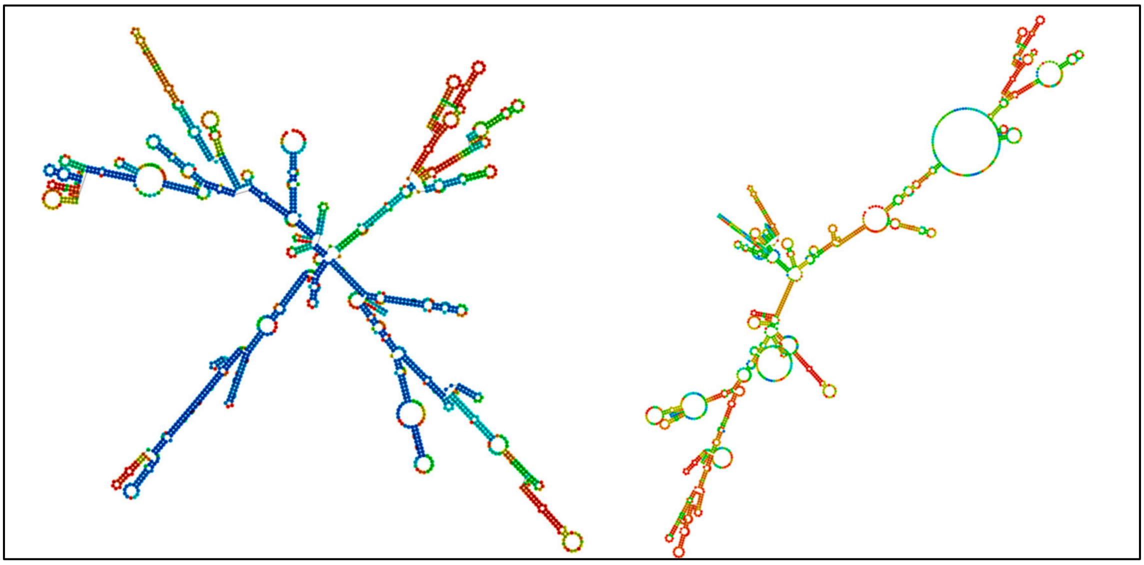

MFE secondary structure and Centroid secondary structure, respectively.

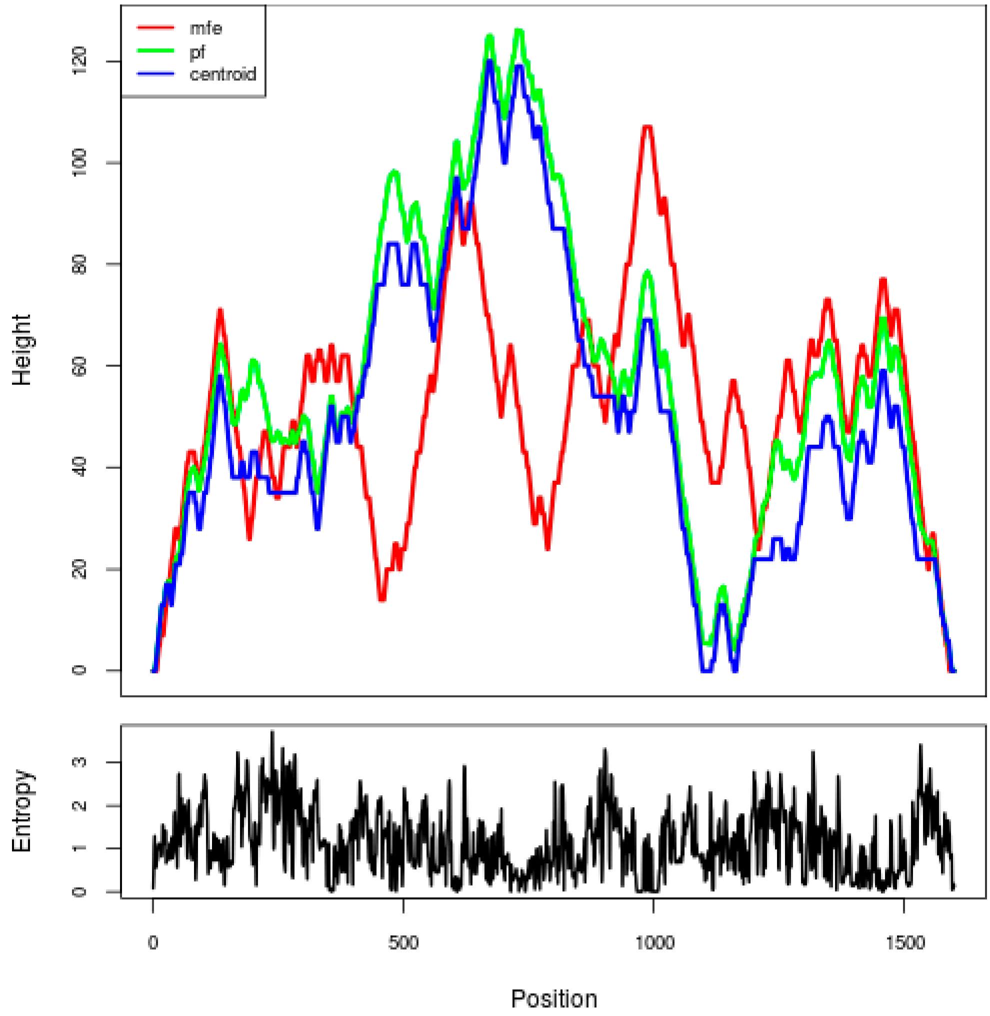

Supplement: Supplementary file 1 [file ncrna-11-00058-s001.zip › ncrna-3703719-supplementary.pdf]
